# Artificial Intelligence-Aided Massively Parallel Spectroscopy of Freely Diffusing Nanoscale Entities

**DOI:** 10.1021/acs.analchem.3c01043

**Published:** 2023-08-08

**Authors:** Antonín Hlaváček, Kateřina Uhrová, Julie Weisová, Jana Křivánková

**Affiliations:** Institute of Analytical Chemistry of the Czech Academy of Sciences, Veveří 97, 602 00 Brno, Czech Republic

## Abstract

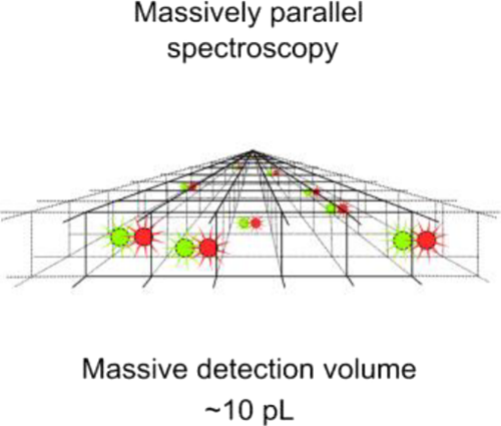

Massively parallel spectroscopy (MPS) of many single nanoparticles in an aqueous dispersion is reported. As a model system, bioconjugated photon-upconversion nanoparticles (UCNPs) with a near-infrared excitation are prepared. The UCNPs are doped either with Tm^3+^ (emission 450 and 802 nm) or Er^3+^ (emission 554 and 660 nm). These UCNPs are conjugated to biotinylated bovine serum albumin (Tm^3+^-doped) or streptavidin (Er^3+^-doped). MPS is correlated with an ensemble spectra measurement, and the limit of detection (1.6 fmol L^–1^) and the linearity range (4.8 fmol L^–1^ to 40 pmol L^–1^) for bioconjugated UCNPs are estimated. MPS is used for observing the bioaffinity clustering of bioconjugated UCNPs. This observation is correlated with a native electrophoresis and bioaffinity assay on a microtiter plate. A competitive MPS bioaffinity assay for biotin is developed and characterized with a limit of detection of 6.6 nmol L^–1^. MPS from complex biological matrices (cell cultivation medium) is performed without increasing background. The compatibility with polydimethylsiloxane microfluidics is proven by recording MPS from a 30 μm deep microfluidic channel.

Lanthanide-doped photon-upconversion nanoparticles (UCNPs) possess short-wavelength emission after long-wavelength excitation (such as 976 nm).^1,2^ The long-wavelength excitation practically avoids autofluorescence and limits scattering. Besides other nanoparticle labels,^3,4^ UCNPs drive the progress of numerous fields of research, including biological imaging, photonics, super-resolution microscopy, single-molecule assays, volumetric displays, security printing, etc.^5−7^ We recently described upconversion-linked immunosorbent assays (ULISAs) for detecting ultralow concentrations of protein markers in body fluids^8^ and environmental micropollutants.^9,10^ Besides conventional assays on solid surfaces, there is a strong interest in developing homogeneous assays, which are less laborious and can be better automated, miniaturized, and scaled to a high throughput. An exciting advancement in this direction is upconversion cross-correlation spectroscopy (UCCS).^11,12^ Similar to other cross-correlation spectroscopies, the UCCS instrumentation is a confocal microscope with a dual-wavelength detector.^13,14^ For UCCS sandwich immunoassays, two types of antibodies are labeled with UCNPs of unique emission wavelengths and mixed with a sample. Then, the increase of cross-correlation amplitude indicates the presence of the immunochemical complexes of the analyte molecules, and this signal can be used for quantification.^11−14^

However, the small detection volume (∼1 fL), slow diffusion, low brightness, and long luminescence decay times of UCNPs prevent the analysis of low concentrations.^11−15^ For instance, the concentration of 1 nmol L^–1^ is equivalent to 0.6 molecules or nanoparticles in 1 fL. The analysis of less concentrated samples becomes increasingly time-consuming—one is waiting for a long time to observe the emission from the detection volume.^15^

For the first time, we introduce massively parallel spectroscopy (MPS) with a significantly larger detection volume to circumvent the limitations of cross-correlation spectroscopy. MPS, also known as slitless spectroscopy, is commonly used in astronomy for the spectroscopy of stars and other cosmic objects.^16,17^ In its simplest setting, a dispersive element such as a prism or a diffraction grating is placed in front of the camera. Then, numerous spectra of stars are projected onto the camera sensor. A way more complex device of this type is on the board of the James Webb Space Telescope where high-throughput analysis of spectra is essential.^18^ Surprisingly, MPS is only rarely used for studies of emission or scattering from single molecules or nanoparticles on solid surfaces.^19−21^ Here, we pioneer MPS for freely diffusing nanoscale entities. MPS is realized by inserting an optical prism in front of the camera sensor in a wide-field epiphoton-upconversion microscope. A convolutional neural network is trained for automatic data processing. The bioaffinity clustering of UCNPs conjugated with biotinylated bovine serum albumin (Tm^3+^-doped, UCNP-Tm-Biotin) and streptavidin (Er^3+^-doped, UCNP-Er-Streptavidin) is studied with MPS as a model system, and a competitive assay for biotin is developed. Finally, the compatibility of MPS with polydimethylsiloxane microfluidics is demonstrated.

## Materials and Methods

Previously published methods were used for nanomaterial synthesis and characterization (Supporting Notes 1–9).^8,22^ A standard photolithography protocol was used for preparing a polydimethylsiloxane microfluidic chip (Supporting Note 10).^23,24^ For a detailed description of instrumentation and data processing, see Supporting Information (Supporting Notes 11, 12, Figures S1, S2). Acetate buffer contained 50 mmol L^–1^ acetic acid, 34 mmol L^–1^ tris(hydroxymethyl)aminomethane, 0.05% w/v NaN_3_, 0.01% w/v Tween 20, and 0.5% w/v bovine serum albumin, pH 5.0.

### Immobilization of Bioconjugated UCNPs

The dispersions of bioconjugated UCNPs were diluted with a dispersion of melted agarose in water tempered at 35 °C (1% w/v, low melting agarose, Carl Roth). The resulting dispersion was cast as a 42 μm layer between two glass slides. After 15 min in the refrigerator (4 °C), the cover glass was removed, and the agarose gel dried rapidly, forming a homogeneous submicron layer with bioconjugated UCNPs.^22^ Before MPS, the layer was dropped with immersion oil and covered with a 170 μm glass slide.

### MPS from an Aqueous Dispersion

For sampling the dispersion, we first cut approximately 1 cm squared “window” into a two-sided plastic tape. This tape was stuck to a standard microscope glass slide of 1 mm thickness. Then, 2 μL of the sample was dropped onto the glass slide in the plastic tape “window” and covered with a 170 μm thick glass slide, which was stuck to the other side of the plastic tape. This resulted in an approximately 80 μm thick layer of the sample dispersion. After dropping the cover glass with an immersion oil, the preparation was put in contact with the microscope objective. The focal plane was buried 10 μm into the investigated dispersion. For a standard MPS experiment, 1000 MPS images with a dimension of 1024 px × 1024 px (111 μm × 111 μm in the plane of the sample) were recorded with 10 ms exposition time and 100 ms intervals between images.

### Dilution Series of UCNP-Er-Streptavidin

The UCNP-Er-Streptavidin was diluted in an acetate buffer, and MPS spectra were recorded.

### Formation of Bioaffinity Clusters and a Competitive Assay of Biotin

The bioconjugated UCNPs were diluted in an acetate buffer. Optionally, a desired concentration of biotin was introduced into the dispersion of UCNP-Er-Streptavidin. After a suitable time of incubation (15 min, 24 °C), the dispersions of UCNP-Tm-Biotin and UCNP-Er-Streptavidin were mixed 1:1 (v/v) and incubated (70 min, 24 °C) to form the bioaffinity clusters. After the incubation, the dispersions were diluted to a suitable concentration, and MPS spectra were recorded.

### MPS from a Microfluidic Chip

UCNP-Er-Streptavidin was dispersed in the cell-cultivation medium (DMEM, Gibco, Thermo Fisher Scientific Inc.) and introduced into the microfluidic polydimethylsiloxane channel (width 100 μm, depth 30 μm). The focal plane of the microscope objective was scanned through the dispersion recording MPS at different depths of the channel.

## Results and Discussion

### Instrumentation

**Figure 1:**
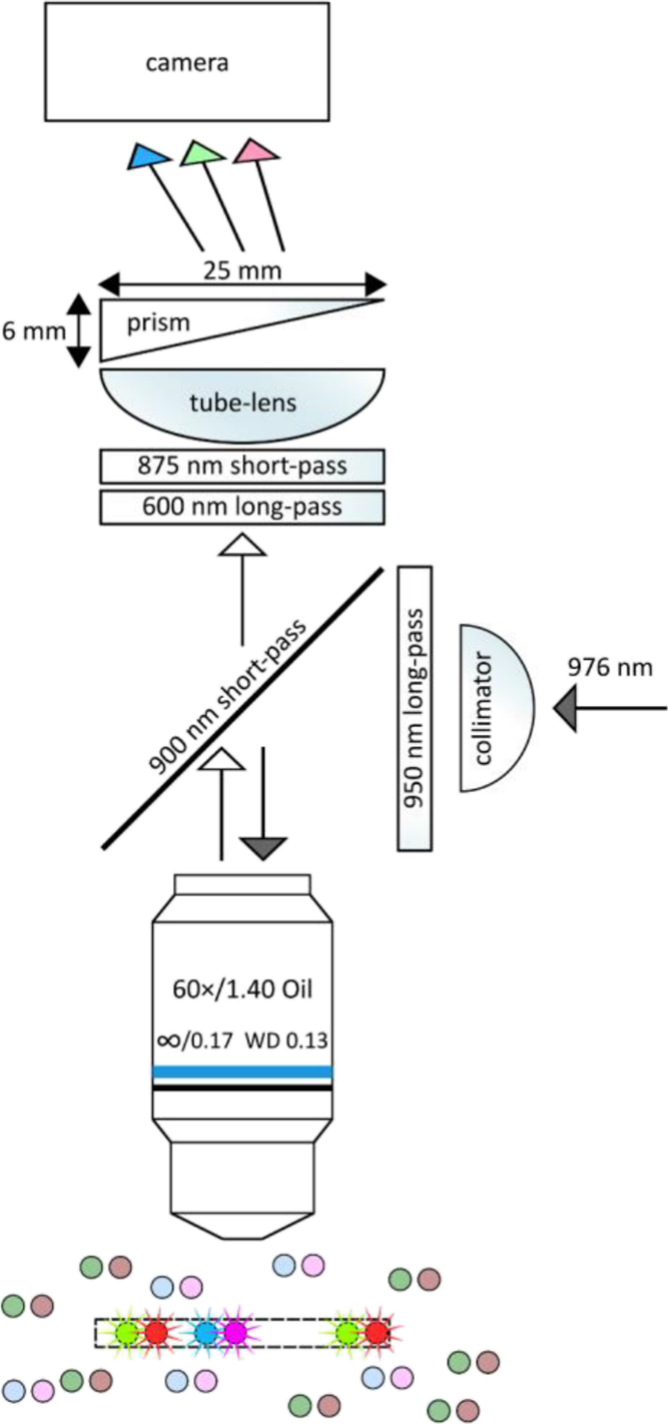
Optical setting of the MPS device. The excitation laser (976 nm, gray arrow) is introduced by a collimator (focal length 4.3 mm), which is connected to the tip of the optical fiber (diameter 105 μm). A long-pass filter removes short wavelengths from a laser beam. A short-pass dichroic mirror reflects the beam through the microscope objective (focal length 3.3 mm) into the dispersion of UCNPs. The focal plane can freely scan into the UCNP dispersion (blue/magenta for Tm^3+^-doped and green/red for Er^3+^-doped UCNPs). Only UCNP spectra in the focal plane (within the detection volume) are imaged brightly (colored stars). The objective collects UCNP emissions (white arrow). The 875 nm short-pass filter protects the camera from excitation wavelengths and can be complemented with other optical filters. A tube-lens (focal length 200 mm) projects the emission into the camera through an optical prism, which disperses the emission (colored arrows) and forms a spectral pattern for each UCNP on the camera sCMOS sensor (the prism is 60 mm in front of the camera sensor).

Figure 1 shows the scheme of the MPS device. The excitation laser beam (976 nm) was projected through an infinity-corrected microscope objective on the sample of UCNPs.

The diameter of the circular observation area where the UCNPs strongly emitted was 111 μm (1024 px on the camera sensor, Figure S1). The illumination within this area was not ideally homogeneous, but this was not a problem for digital reading of emission spectra as discussed later. The power of the excitation beam transmitted with the microscope objective was 1.3 W, resulting in an average excitation intensity in the observation area of 9.4 kW cm^–2^. In contrast to a common microscope instrumentation, a poly(methyl methacrylate) prism was placed between the tube lens and the camera. The prism dispersed the UCNP emission, projecting the emission spectra on the camera sensor for every single nanoparticle. A similar optical setting is quite common in astronomy when the spectra of stars are studied.^16^ The use of a prism as a dispersing element was proven more effective than using a diffraction grating.^16,17^ In contrast to light dispersion, the mask of optical filters, such as red-green-blue pixels, can also provide rudimental spectra information. However, the mask reduces the attainable signal by absorbing the photons and cannot be easily replaced for the wavelengths of interest. Another alternative is using a set of dichroic mirrors projecting different wavelengths separately onto the different parts of the camera sensor. However, a precise alignment of more optical components complicates the instrumentation, and the setup is less flexible for changing the wavelengths of detection. Additionally, the area of the camera sensor is not optimally utilized when using different parts of the sensor for different wavelengths.

### Bioconjugated UCNPs

**Figure 2:**
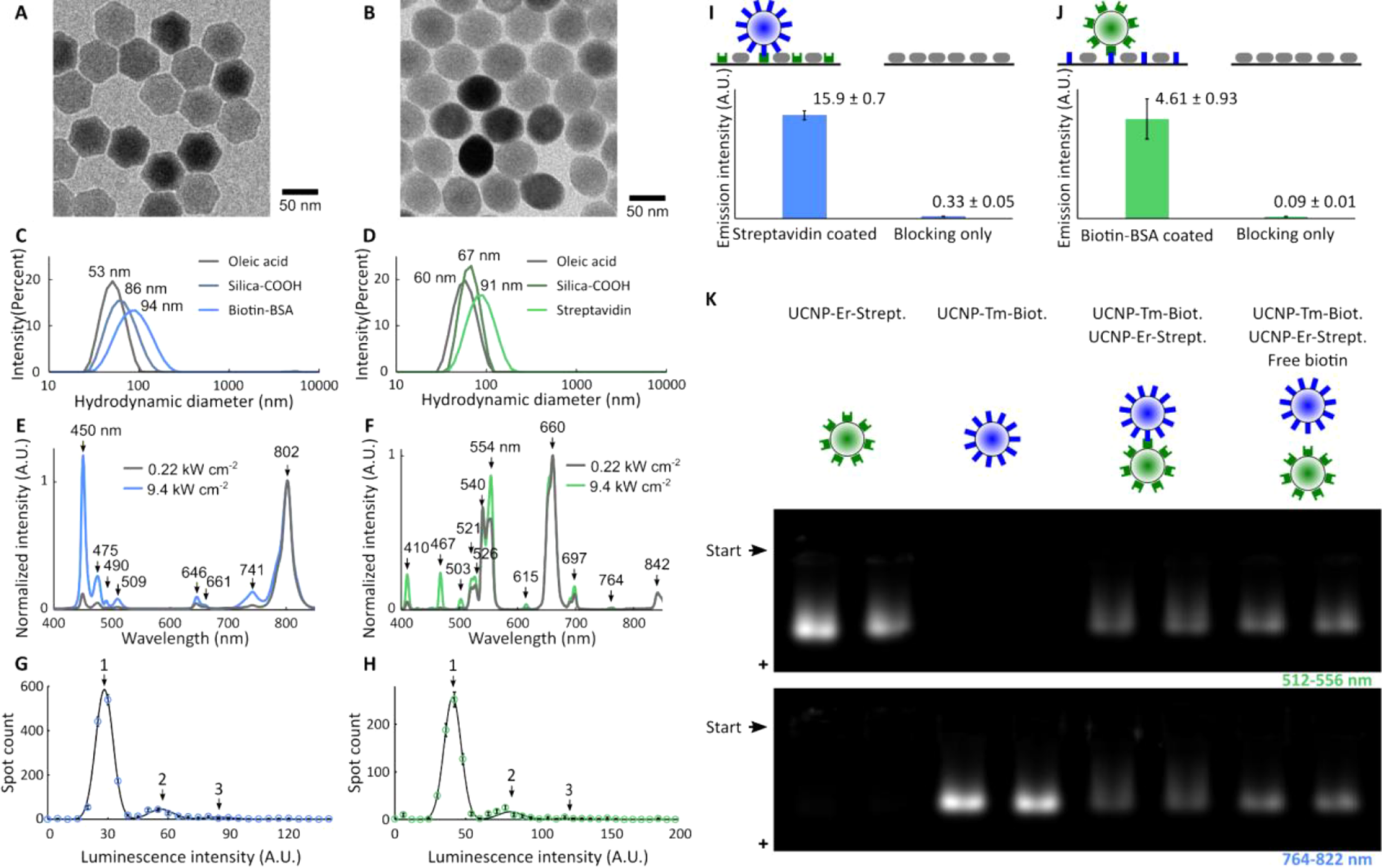
Nanomaterial characterization. (A, B) TEM micrographs of (A) Tm^3+^-doped and (B) Er^3+^-doped oleic acid-capped UCNPs (see Figures S4 and S5 for full images); (C,D) hydrodynamic diameters of UCNPs with different surface modifications (C) Tm^3+^-doped, (D) Er^3+^-doped; (E, F) emission spectra of (E) UCNP-Tm-Biotin and (F) UCNP-Er-Streptavidin (excitation wavelength 976 nm, intensity as indicated in the panels, normalized at 802 and 660 nm, respectively); (G, H) histograms of spot intensities of agarose-immobilized bioconjugated UCNPs with a marked number of UCNPs per spot (G, UCNP-Tm-Biotin, H, UCNP-Er-Streptavidin); (I, J) specificity assay of bioconjugated UCNPs; (I) binding UCNP-Tm-Biotin on a microtiter plate coated with streptavidin (green) and biotin-free BSA (gray); (J) binding UCNP-Er-Streptavidin on a microtiter plate coated with biotin-BSA (blue) and biotin-free BSA (gray), and emission from the surface is plotted as averages ± standard deviations from three replica wells; (K) native gel electrophoresis of bioconjugated UCNPs, with samples loaded in duplicates. The formation of bioaffinity clusters between UCNP-Tm-Biotin and UCNP-Er-Streptavidin broadens the electrophoresis zone. The broadening did not occur in the presence of free biotin. (I–K) Excitation at 976 nm, emission at 764–822 nm (UCNP-Tm-Biotin), and 512–556 nm (UCNP-Er-Streptavidin).

Oleic acid-capped UCNPs with the composition of NaY_0.80_Yb_0.18_Tm_0.02_F_4_ core-only and NaY_0.80_Yb_0.18_Er_0.02_F_4_/NaYF_4_ core/shell were prepared via a seed-mediated growth.^8,25,26^ Transmission electron microscopy (TEM, Figure 2A,B) revealed a hexagonal shape of Tm^3+^-doped particles with a diameter of 61.4 ± 2.2 nm. The hydrodynamic diameter was 53 nm (from dynamic light scattering with intensity-weighed distribution), suggesting that the shape of nanoparticles was rather a nanoplate (not visible on the TEM micrographs). The Er^3+^-doped particles were oval with a longer dimension of 63.2 ± 2.3 nm and a shorter dimension of 53.4 ± 2.7 nm; the hydrodynamic diameter was 60 nm. The UCNPs were coated with a layer of carboxylated silica to prepare water-dispersible nanoparticles.^8^ Carbodiimide chemistry was used for attaching biotinylated bovine serum albumin (biotin-BSA) to Tm^3+^-doped nanoparticles (UCNP-Tm-Biotin) and streptavidin to Er^3+^-doped nanoparticles (UCNP-Er-Streptavidin).^8^ The process of silica coating and bioconjugation indicated the increasing hydrodynamic diameters: after silica coating 86 or 67 nm and after bioconjugation 94 or 91 nm for Tm^3+^- or Er^3+^-doped particles, respectively (Figure 2C,D).

To measure the bulk emission spectra of bioconjugated UCNPs under a high excitation intensity (9.4 kW cm^–2^), the nanoparticles were immobilized in a submicron layer of agarose.^22^

These samples were inserted into the MPS device, but the optical prism was replaced with a collimator to connect a CCD spectroscope (QE65Pro from Ocean Optics). The emission spectra were quite different from the spectra recorded at low excitation intensities, which is a result of populating higher energy levels of Tm^3+^ and Er^3+^ (Figure 2E,F).^6,27,28^ For instance, the blue emission of UCNP-Tm-Biotin at 450 nm was of comparable intensity with near-infrared emission at 802 nm, and UCNP-Er-Streptavidin revealed an unusual emission at 467 nm.

An absolute counting method^22^ was used for estimating the molar concentrations of bioconjugated UCNPs (i.e., the number of particles in a given volume divided with Avogadro’s number). The nanoparticles were immobilized in a submicron agarose layer, imaged as bright spots by an epiphoton-upconversion microscope, and counted. The content of not aggregated nanoparticles was 77 and 81% for UCNP-Tm-Biotin and UCNP-Er-Streptavidin, respectively (estimated from the histograms of spot intensities, Figure 2G,H).^22^

The bioaffinity of bioconjugated UCNPs was tested with a microtiter plate assay and native gel electrophoresis. The microtiter plates were coated with either biotinylated-BSA or streptavidin. The non-specific binding sites were blocked with biotin-free BSA. Only biotin-free BSA blocking was used for negative controls. Compared to the negative controls, positive wells had 48× or 51× higher luminescence from UCNP-Tm-Biotin or UCNP-Er-Streptavidin, respectively (Figure 2I,J). Native agarose electrophoresis proved the formation of bioaffinity clusters in dispersion. Because of the larger size, the electrophoretic mobility of clustered nanoparticles in the agarose gel is lower than the electrophoretic mobility of not clustered UCNPs, as we reported previously.^8,29,30^ This led to zone broadening (Figure 2K) when a part of the sample migrates with an unchanged rate (not clustered UCNPs) and a part of the sample has lower electrophoretic mobility (UCNP clusters). The size of pores in the utilized agarose gel was ∼500 nm,^31^ which means that the size of bioaffinity clusters counts maximally several UCNPs (hydrodynamic diameter ∼ 90 nm).

### MPS on a Solid Support

**Figure 3:**
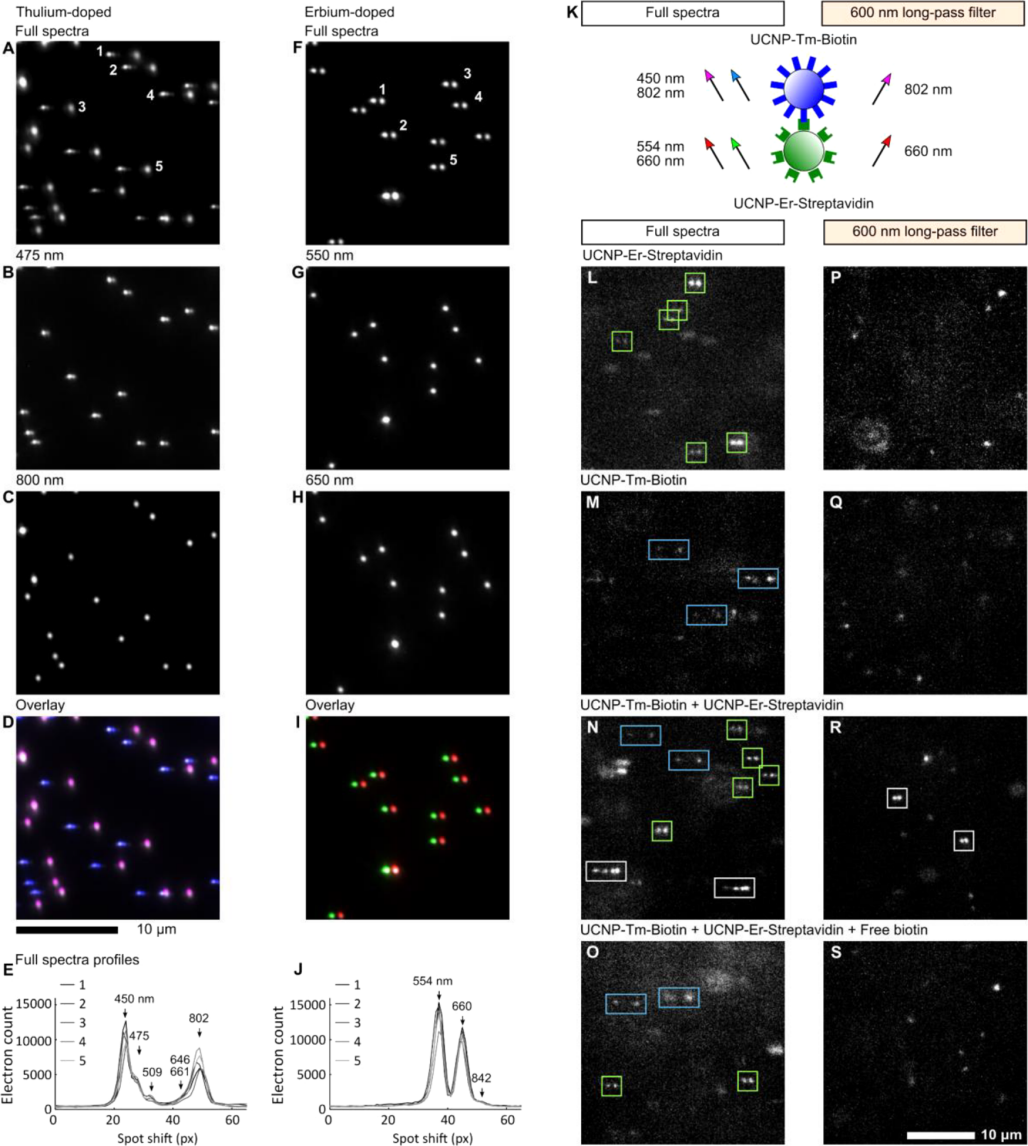
MPS. (A–J) Bioconjugated UCNPs in the agarose submicron layer (2000 ms exposition). (A–E) UCNP-Tm-Biotin; (F–J) UCNP-Er-Streptavidin; (A, F) full emission spectra (430–850 nm); (B) spectra with a 475 ± 25 nm band-pass filter; (C) 800 ± 25 nm; (G) 550 ± 25 nm; (H) 650 ± 25 nm; (D) overlay of panels A–C: blue 475 ± 25 nm filter, magenta 800 ± 25 nm filter, gray full spectra; (I) overlay of panels F–H: green 550 ± 25 nm filter, red 650 ± 25 nm filter, gray full spectra; (E, J) spectra profiles from (A) and (F), respectively; (K) emission wavelengths from bioaffinity clusters with or without a 600 nm long-pass filter; (L–S) bioconjugated UCNPs in an aqueous buffer (10 ms exposition); (L–O) full emission spectra, (P–S) 600 nm long-pass spectra; green rectangles for UCNP-Er-Streptavidin, blue for UCNP-Tm-Biotin, white for bioaffinity clusters. See Figures S6–S21 for full MPS images.

For testing the MPS device, we immobilized the bioconjugated UCNPs in a submicron layer of agarose on a glass substrate.^22^ After dropping with an immersion oil and covering with a 170 μm glass slide, the sample was inserted into the MPS device. Without additional optical filters, we recorded the emission spectra of UCNPs from 430 to 875 nm with 2000 ms exposition time (Figure 3A,F). For both samples, the spectra appeared as a series of diffraction- and dispersion-limited spots. In the case of UCNP-Tm-Biotin, double spots with a separation of 30.2 ± 0.8 px were observed (pixel size 6.5 μm, magnification 60×, Figure 3A). The “left” spots were narrower than the “right” spots, which suggested shorter wavelengths on the “left” side. For confirming, we inserted additional blue (475 ± 25 nm band-pass) or near-infrared (800 ± 25 nm band-pass) filters in front of the prism (Figure 3B,C). The narrower “left” peaks were visible through the blue filter and the wider “right” ones through the near-infrared filter. Then, we confirmed the spectra pattern by overlying the full spectra images with images recorded through band-pass filters (Figure 3D). When compared with the ensemble emission spectra (Figure 2E), it was possible to assign the spot separation of 30.2 ± 0.8 px to a wavelength separation of 352 nm (emission maxima at 450 and 802 nm). Very similarly, spectra patterns from UCNP-Er-Streptavidin revealed double spots separated with a distance of 9.4 ± 0.5 px (Figure 3F). By applying a 550 ± 25 or a 650 ± 25 nm band-pass filter, it was possible to assign these spots to their wavelengths (554 and 660 nm, Figure 3G–I). The spot separation of 9.4 ± 0.5 px was equivalent to the wavelength separation of 106 nm in the ensemble emission spectra (Figure 2F). The profiles of full spectra were plotted for several single UCNPs, and finer spectra features were recognized (Figure 3E,J). The “blue” spot of UCNP-Tm-Biotin was composed of three maxima (450, 475, and 509 nm). The emission at 646 and 661 nm appeared as a shoulder of the “near-infrared” peak. Similarly, the “red” peak of UCNP-Er-Streptavidin had a shoulder of 842 nm emission.

### MPS of Bioconjugated UCNPs in a Dispersion

The optimal exposition time for recording MPS from aqueous dispersion was 10 ms, which provided a high enough signal and a negligible diffusion move. After diluting the bioconjugates to 10 pmol L^–1^, we observed the same spectra patterns as those from immobilized UCNPs. However, finer features were lost as a result of the shorter exposition time; only the patterns of double spots were observable (Figure 3K–M).

Streptavidin and biotin are well known for their strong bioaffinity interactions forming stable bioaffinity complexes (when considering streptavidin- and biotin-BSA-conjugated nanoparticles, the term bioaffinity cluster is better fitting the resulting structure, Figure 3K). Encouraged with measuring MPS from single diffusing nanoparticles, we were just eager to observe the bioaffinity clusters of UCNP-Tm-Biotin and UCNP-Er-Streptavidin. We prepared a mixed dispersion containing UCNP-Tm-Biotin (0.5 nmol L^–1^) and UCNP-Er-Streptavidin (0.5 nmol L^–1^). This mixture was incubated for 70 min at laboratory temperature and then 100× diluted for MPS (Figure 3N). Again, we observed the spectra patterns of UCNPs-Tm-Biotin and UCNP-Er-Streptavidin. However, these were accompanied by a pattern of four spots. Most likely, these were spectra from bioaffinity clusters of UCNPs-Tm-Biotin and UCNP-Er-Streptavidin. We tested this hypothesis in a negative control experiment. A dispersion was prepared containing UCNP-Tm-Biotin (0.5 nmol L^–1^), UCNP-Er-Streptavidin (0.5 nmol L^–1^), and biotin (50 μmol L^–1^). After 70 min incubation at laboratory temperature, the sample was diluted 100× to measure the MPS spectra (Figure 3O). As expected, no four-spot pattern was observed—50 μmol L^–1^ biotin saturated the binding sites of UCNP-Er-Streptavidin.

Thus, MPS established itself as a new method for investigating bioaffinity interactions. However, the whole spectra covered a large area in the MPS images, which was not optimal for analysis. To pursue a practical use, we inserted an additional 600 nm long-pass filter before the optical prism (Figure 1), and we repeated the experiment (Figure 3P–S). For samples containing only UCNP-Tm-Biotin or only UCNP-Er-Streptavidin, the MPS images revealed only isolated spots of either 802 or 660 nm emission belonging to Tm^3+^ or Er^3+^, respectively (Figure 3P,Q). Practically, the same result was received from mixed bioconjugated UCNPs with free biotin (Figure 3S). However, mixing only bioconjugated UCNPs resulted in a bioaffinity clustering, which was observed as double spots separated with a distance of 6.5 ± 0.6 pixels. Indeed, this double spot pattern was more convenient for machine processing (Figure 3R).

### Double Spot Counting

**Figure 4:**
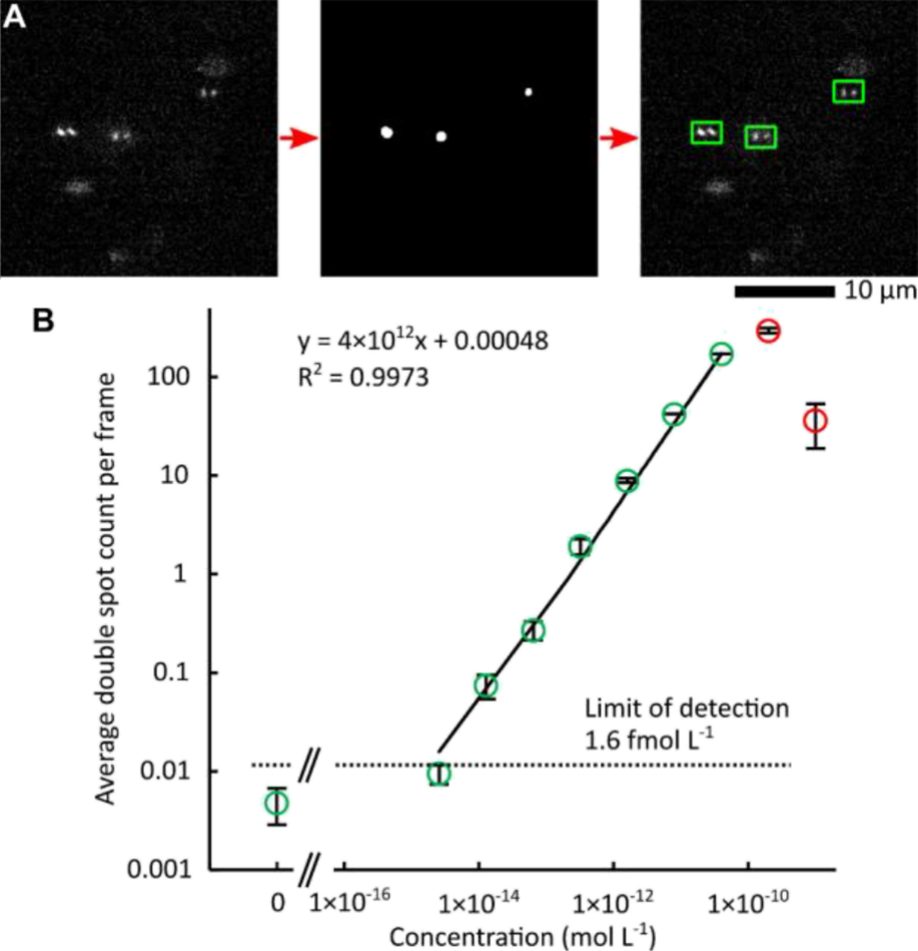
Double spot counting. (A) In the original MPS image (left), the double spots are localized by U-net and subsequently presented as a binary mask (in the middle) and then counted. For a manual revision, the double spots are marked with green rectangles (right). (B) Average number of double spots per frame is plotted for a dilution series of UCNP-Er-Streptavidin; red circles are out of the linear range (averages ± standard deviations from two repeated experiments are plotted for each dilution).

For localization and counting of double spots (Figure 4A), a convolutional network^32,33^ with a U-net architecture^34^ was trained (Figure S2). The linearity of double spot counting was tested on the dilution series of UCNP-Er-Streptavidin in acetate buffer. For each dilution, two glass slides were prepared, MPS images were recorded, and the average number of double spots per image was calculated (Figure 4B). The limit of UCNP-Er-Streptavidin detection was 1.6 fmol L^–1^ (calculated as 3.3 standard deviations of the blank divided by the slope of the calibration curve), and the linear range spanned 4 orders of magnitude from 4.8 fmol L^–1^ up to 40 pmol L^–1^ with a coefficient of determination *R*^2^ = 0.9973. At higher concentrations, the overlaps of double spot patterns caused a decline from linearity. See Figures S22–S31 for representative MPS images with a localization of double spots for each dilution.

### MPS Competitive Assay of Biotin

Double spot counting was used for a competitive assay of biotin. The dispersions of UCNP-Er-Streptavidin in acetate buffer (1 nmol L^–1^) were supplemented with a variable concentration of biotin and incubated for 15 min at laboratory temperature. These dispersions were 1:1 (v/v) mixed with 1.0 nmol L^–1^ UCNP-Tm-Biotin. After 70 min incubation at laboratory temperature, the dispersions were 100× diluted. For each dilution, two glass slides were prepared, MPS was recorded, and the average number of double spots per image was estimated. See Figures S32–S39 for representative MPS images with localization of double spots for each biotin concentration.

**Figure 5:**
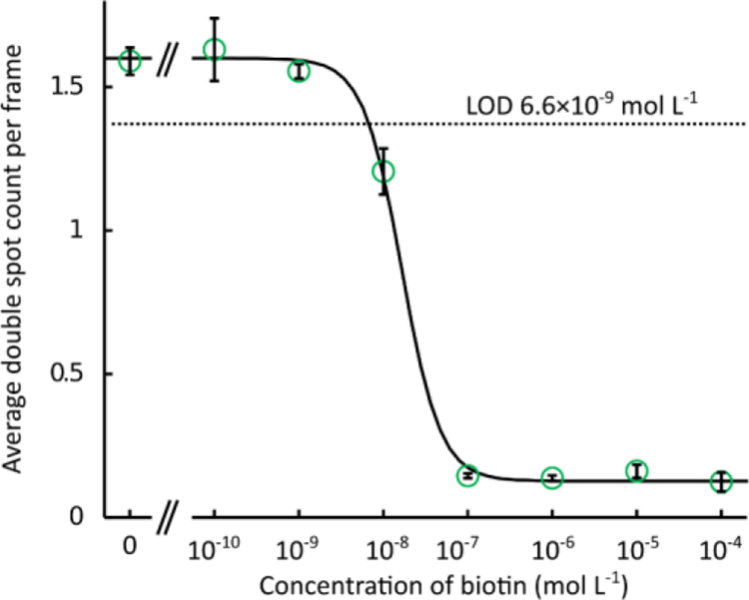
MPS competitive assay of biotin. The average double spot count per frame decreases with increasing concentration of biotin (averages ± standard deviations from two repeated experiments are plotted for each biotin concentration).

As a result of saturating the biotin binding sites, the number of observed double spots per frame decreased with the increasing concentration of biotin (Figure 5). A four-parameter logistic function (eq 1) was used as a regression model, where [biotin] is the molar concentration of biotin, and DSC is the average double spot count per frame. The parameters are the maximum DSC (DSC_max_), the background DSC (DSC_bg_), the biotin concentration that reduces DSC_max_ – DSC_bg_ by 50% (IC_50_), and the slope (*S*) at the inflection point.
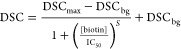
1

The DSC corresponding to the limit of detection (DSC_LOD_) was estimated as follows (eq 2):
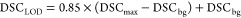
2

The limit of detection (LOD) for biotin was calculated from inverted eq 1 for DSC_LOD_. The LOD for biotin was 6.6 nmol L^–1^, and the value of IC_50_ was 17 nmol L^–1^. This LOD can be compared with the LOD of previously published competitive ULISA for similarly small molecules, although the detection mechanism is slightly different (not involving antibodies). For instance, we have reported two assays for diclofenac with LODs 170 and 70 pmol L^–1^.^9,10^ A comparison is also made to a homogeneous luminescence resonance energy-transfer-based competitive assay of biotin with a reported IC_50_ of 700 pmol L^–1^. Additionally, a heterogeneous competitive assay of biotin on a microtiter plate was tested by applying the UCNP-Tm-Biotin and UCNP-Er-Streptavidin (Supporting Note 13, Figure S3). The LOD for biotin was 17 pmol L^–1^, and IC_50_ was 58 pmol L^–1^. These findings are consistent with other literature reports where heterogeneous bioaffinity assays tend to be more sensitive.^35^ However, the development of homogeneous bioaffinity assays is of high interest for their easier use.^35^

### Comparing MPS to Related Methods

Fluorescence cross-correlation spectroscopy (FCCS) utilizes a confocal microscope with a dual-wavelength detector for observing the correlation of fluorescence from interacting molecules, which are labeled with two different fluorophores.^13,14,36^ FCCS is, to some extent, limited by autofluorescence and by relatively long acquisition times.^11,12,36,37^ For instance, the LOD of a sandwich immunochemical assay for human chorionic gonadotropin in phosphate-buffered saline was 100 pmol L^–1^ by using 30 min FCCS data series.^37^ The autofluorescence problem was recently resolved by introducing photon-upconversion labels in UCCS.^11,12^ In this context, we speculate that MPS can be seen as an advancement to the UCCS. Counting the number of double spots in the MPS images is an alternative to a cross-correlation amplitude^11−14^ in the cross-correlation spectroscopy—both quantities are directly proportional to the concentration of emitting particles. However, the discrete nature of counting provides a benefit of a noise-free processing. Another advancement is the size of the detection volume. While approximately 1 fL detection volumes are typical for cross-correlation spectroscopy,^13,14,36^ a much larger detection volume is provided with MPS. The concentration of 10 pmol L^–1^ equals to ∼6 particles per 1000 fL. At this concentration, we observed on average 40 double spots per frame (Figure 4B), which implies a detection volume of ∼6700 fL. This detection volume had a disc shape with a diameter of 111 μm and a thickness of 0.7 μm. Another important parameter when considering the throughput is exposition time (MPS) or sampling time (cross-correlation spectroscopy). In our setting, we used 10 ms exposition time, which is a bit longer than 1 or 2 ms sampling in UCCS.^11,12^ Putting together, MPS can scan approximately 10^3^× larger volume of dispersion in the same unit of time, which supports the analysis of more diluted samples and lower LODs.^15^ On the other hand, we should note that very sensitive counting of double spots in MPS does not directly ensure ultrasensitive bioaffinity assays. Similar to other methods, the assays can be limited by the equilibrium dissociation constant of the bioaffinity pair. For instance, antibody–antigen complexes have dissociation constants typically from 10 pmol L^–1^ to 10 nmol L^–1^.^35,38^

A wide-field fluorescence microscope with a dual-channel image-splitting system and a digital camera was used to also increase the detection volume in FCCS.^39^ The limitation of this imaging FCCS is, however, a need for optical sectioning by single-plane illumination or total internal reflection, which requires specialized sample preparation and precise optical alignment.^39^

When discussing homogeneous assays, we should also note assays utilizing resonance energy transfer.^40^ This is most commonly realized by fluorescence labeling of interacting molecules with energy donors and energy acceptors.^40^ To avoid autofluorescence, photon-upconversion labels were also utilized in this assay format.^41^ Compared to MPS, a general limitation is a need for proximity (∼1–20 nm) between the donor and the acceptor, otherwise the resonance transfer is not efficient.^40,41^ This can be difficult with nanoparticle labels for their larger size (up to ∼100 nm), surface coating (thickness ∼ 1–10 nm), and competing non-radiative surface deactivation.^42,43^

### Applicability

**Figure 6:**
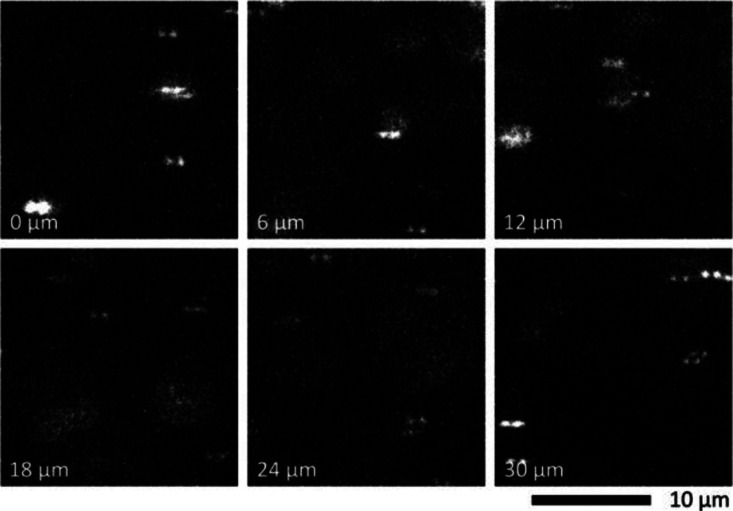
MPS from the microfluidic channel. The channel contained UCNP-Er-Streptavidin (10 pmol L^–1^). The focal plane was scanned in six steps from the glass substrate (0 μm) to the polydimethylsiloxane ceiling of the channel (30 μm depth). See Figures S40–S45 for full MPS images.

Recently, there has been an increasing need for microfluidic bioaffinity assays.^35^ Therefore, we tested the compatibility of polydimethylsiloxane microfluidics with MPS (Figure 6). After filling the microfluidic channel with a dispersion of UCNP-Er-Streptavidin (10 pmol L^–1^) in a cell cultivating medium, we were able to record the MPS spectra through an entire 30 μm depth of the channel. Such results suggest applicability for single-cell bioaffinity assays in droplet microfluidics.^44^ One may enclose single cells in droplets and perform a sandwich immunoassay with two types of antibodies conjugated with two types of UCNPs. When expecting the droplet diameter of 100 μm and thickness of 30 μm, the entire droplet volume can be scanned in 43 steps of 0.7 μm length (the thickness of the detection volume). With 10 ms exposition time and 2 ms for transiting the focal plane, one may count virtually all bioaffinity clusters in the droplet within 516 ms. MPS is principally not limited to photon-upconversion. Microscope modalities such as fluorescence, dark-field, and bright-field are available for other nanoparticle types; quantum dots, polymeric fluorescent nanoparticles, and plasmonic nanoparticles are of high interest.^4,45,46^ For instance, a process of clustering in real-time and spectral properties such as brightness can be measured on a large, yet single-particle, scale. Another field is ratiometric nanosensors where MPS can perform massively parallel nanosensing.^47^

## Conclusions

MPS of freely diffusing nanoparticles in an aqueous dispersion is described for the first time. Particles emitting two emission wavelengths appear as double spots in the MPS images. The counting of double spots per MPS image is principally comparable to the cross-correlation amplitude in cross-correlation spectroscopy—both quantities can be used for quantification. However, MPS possesses much larger detection volumes and operates digitally; MPS can scan approximately 10^3^× larger volume of dispersion in the same unit of time. MPS was proven suitable for observing the bioaffinity clustering and bioaffinity assays freely in an aqueous dispersion. Because MPS is a new technique, the applications are only limited by the imagination and the persistence of the experimenter. Besides characterizing freely diffusing molecules and nanoparticles of diverse types, we speculate on homogeneous immunochemical assays and ratiometric nanosensors for high-throughput microfluidics. Additional imaging modalities like fluorescence, dark-field, and bright-field are of high interest.

## Supporting Information Available

Nanomaterial synthesis and characterization, microfluidic chip fabrication, data processing, detailed description of the instrumentation, and supporting micrographs (PDF)
