# Supplementary material for: Artificial Intelligence-Aided Massively Parallel Spectroscopy of Freely Diffusing Nanoscale Entities
Source: Anal Chem. 2023 Aug 8;95(33):12256–63. doi: 10.1021/acs.analchem.3c01043 (PMC10448498; doi:10.1021/acs.analchem.3c01043)
Supplement: Supplementary file 1 — ac3c01043_si_001.pdf [file ac3c01043_si_001.pdf]

Supporting Information

Artificial Intelligence-Aided Massively Parallel Spectroscopy of Freely Diffusing Nanoscale Entities

Antonín Hlaváček*, Kateřina Uhrová, Julie Weisová, Jana Křivánková

Institute of Analytical Chemistry of the Czech Academy of Sciences, 602 00 Brno, Czech Republic

Contents

Supporting Note 1. Chemicals 2
Supporting Note 2. Buffer solutions 2
Supporting Note 3. Synthesis of $NaY_{0.80}Yb_{0.18}Er_{0.02}F_4/NaYF_4$ UCNPs capped with oleic acid 2
Seed nanoparticle synthesis 2
Core growth 3
Shell growth 3
Supporting Note 4. Synthesis of $NaY_{0.80}Yb_{0.18}Tm_{0.02}F_4$ UCNPs capped with oleic acid 4
Seed nanoparticle synthesis 4
Core growth 4
Supporting Note 5. Coating the nanoparticles with carboxylated silica 5
Supporting Note 6. Preparation of biotinylated bovine serum albumin 5
Supporting Note 7. Preparation of biotinylated nanoparticles (UCNP-Tm-Biotin) 5
Supporting Note 8. Preparation of streptavidin-conjugated nanoparticles (UCNP-Er-Streptavidin) 6
Supporting Note 9. Nanomaterial characterization 6
Estimating nanoparticle hydrodynamic diameters by dynamic light scattering 6
Transmission electron microscopy 6
Estimating the mass concentration of nanoparticle dispersion 6
Estimating molar concentration of bioconjugated UCNPs 6
Specificity assay of UCNP-Tm-Biotin 6
Specificity assay of UCNP-Er-Streptavidin 7
Native gel electrophoresis of bioconjugated UCNPs 7
Supporting Note 10. Manufacturing the polydimethylsiloxane microfluidic chip 8
Supporting Note 11. Instrumentation 8
Instrument configaration 8
Characterization of the excitation laser and luminescence saturation of UCNPs 8
Supporting Note 12. Processing MPS images 12
Supporting Note 13. Competitive assay of biotin on microtiter plates 13
Assay of biotin with UCNP-Tm-Biotin 13
Assay of biotin with UCNP-Er-Streptavidin 13
Micrographs 14
References 55

Supporting Note 1. Chemicals

From Merck / Sigma-Aldrich (St. Louis, MO, USA) were biotin (>99%), biotin N-hydroxysuccinimide ester (biotin-NHS, >98%), Igepal CO-520, tetraethyl orthosilicate (99%), N-(3-dimethylaminopropyl)-N′-ethylcarbodiimide hydrochloride (EDC, 98%), N-hydroxysulfosuccinimide sodium salt (sulfo-NHS, 98%), tween 20, $YCl_3 \times 6\,H_2O$ (99.99%), Y_2O_3 (99.99%), Tm_2O_3 (99.99%), $YbCl_3 \times 6\,H_2O$ (99.998%), $TmCl_3 \times 6\,H_2O$ (99.998%), Yb_2O_3 (99.99%), $ErCl_3 \times 6\,H_2O$ (99.99%), Er_2O_3 (99.99%), NH_4F (>98%), octadec-1-ene (technical grade, 90%), oleic acid (technical grade, 90%), sodium oleate (≥82% fatty acids), NaN_3 (>99.5%).

From P-lab (Prague, Czech Republic) were 2-(N-morpholino)ethanesulfonic acid monohydrate (MES, >99%), 3-(N-morpholino)propanesulfonic acid (MOPS, 99.5%), tris(hydroxymethyl)aminomethane (Tris, 99.9%), trifluoroacetic acid (99%), bovine serum albumin (BSA, 98%), agarose (LOW MELT Roti®garose).

Streptavidin (affinity purified) was from Thermo Fisher Scientific (Waltham, Massachusetts, USA).

Carboxyethylsilanetriol sodium salt (25 % w/v in water) was from ABCR (Karlsruhe, Germany).

From Penta (Prague, Czech Republic) were ammonium hydroxide 25% (w/v, p.a.), cyclohexane (p.a.), methanol (p.a.), acetone (p.a.), $NaHCO_3$ (p.a.), Na_2CO_3 (p.a.), NaOH (p.a.), dimethylformamide (DMF, p.a.), propan-2-ol (p.a.), glycerol (p.a.).

Supporting Note 2. Buffer solutions

The MOPST buffer contained 25 mmol L^{-1} MOPS and 0.05% Tween 20. The pH was set to 7.5 by adding NaOH 10 mol L^{-1}.

The acetate buffer contained 50 mM acetic acid, 34 mM Tris, 0.05% (w/v) NaN_3, 0.01% (w/v) Tween 20, 0.5% (w/v) BSA.

The MES buffer contained 100 mmol L^{-1} MES. The pH was set to 6.0 by adding NaOH 10 mol L^{-1}.

The assay buffer contained 50 mM Tris, 0.01% (w/v) Tween 20, 0.05% (w/v) NaN_3, and 0.5% (w/v) BSA. The pH was set to 7.0 by adding HCl 1 mol L^{-1}.

The coating buffer contained 100 mM H_3BO_3, 45 mM Na_2CO_3, and 0.05% NaN_3. The pH of the buffer was 9.0 after dissolving the chemicals.

Supporting Note 3. Synthesis of $NaY_{0.80}Yb_{0.18}Er_{0.02}F_4/NaYF_4$ UCNPs capped with oleic acid

Seed nanoparticle synthesis

$YCl_3 \times 6\,H_2O$ (874 mg, 2.88 mmol), $YbCl_3 \times 6\,H_2O$ (251 mg, 0.648 mmol), and $ErCl_3 \times 6\,H_2O$ (27.5 mg, 0.072 mmol) were dissolved in 30 mL of methanol and added into a 250-mL three-neck round-bottom flask containing 27 mL (24.2 g) of oleic acid and 63 mL (49.7 g) of 1-octadecene. The solution was heated to 170 °C under a protective nitrogen atmosphere until all volatile liquids were evaporated (~60 min). After the temperature had decreased to 50 °C, the nitrogen atmosphere was disconnected, and a solution of 533 mg (14.4 mmol) of NH_4F and 360 mg (9 mmol) of NaOH in 30 mL of methanol was added under intense stirring. The nitrogen atmosphere was reconnected, and the solution was stirred for 30 min. The temperature was carefully increased up to 150 °C, avoiding extensive boiling to ensure the evaporation of methanol. After that, the solution was rapidly heated at the rate of ~10 °C min^{-1}. At 290 °C, the heating was carefully adjusted to 300 °C within one or two minutes. The flask was kept under nitrogen flow at 300 °C (± 4 °C) for 90 min and then let to cool down to room temperature. The UCNPs were precipitated by adding 180 mL of propan-2-ol and collected by centrifugation (1,000*g*, 10 min). The precipitate was dispersed in 20 mL of cyclohexane. After adding 100 mL of methanol, the UCNPs precipitated rapidly without centrifugation. The precipitate was dispersed in 30 mL of cyclohexane and slowly centrifuged (50*g*, 20 min) to separate solid compounds from the final UCNPs.

Core growth

Under reflux, Y_2O_3 (1355 mg, 6.00 mmol), Yb_2O_3 (532 mg, 1.35 mmol), and Er_2O_3 (57.9 mg, 0.15 mmol) were dissolved in trifluoroacetic acid (12 mL) and water (12 mL) in a 250-mL three-necked flask. When dissolved, $NaHCO_3$ (1260 mg, 15.00 mmol) was added, releasing CO_2 bubbles and dissolving rapidly. After removing the condenser, excessive trifluoroacetic acid and water were evaporated by heating at 110 °C in a fume hood (overnight). Together with sodium oleate (1644 mg, 5.4 mmol), the resulting white powder of trifluoroacetates was dissolved in oleic acid (45 mL, 40.3 g), and octadec-1-ene (45 mL, 35.5 g). This solution was diluted with 30 mL of methanol. The methanol together with oxygen and water were removed by heating at 110 °C under the N_2 atmosphere for 20 min, resulting in a precursor solution. The precursor solution was enclosed in the flask by silicon septa and kept under an inert atmosphere. To decrease the viscosity, the precursor solution was kept at an elevated temperature (~50 °C), which facilitated its injection into the reaction mixture. The concentration of $Re(CF_3CO_2)_3$ in the precursor solution was 0.17 mmol mL^{-1} (Re is for Y, Yb, Er in molar percentages 80%, 18%, and 2%, respectively).

The nanoparticles were grown by gradually adding the precursor solution to the dispersion of seed nanoparticles. The dispersion of seed nanoparticles (205 mg) in cyclohexane was mixed with oleic acid (5.5 mL, 4.9 g), octadec-1-ene (17 mL, 13.4 g), sodium oleate (365 mg, 1.2 mmol) and 20 mL of methanol in a 100-mL three-necked flask. The mixture was heated at 150 °C for ~30 min under the nitrogen atmosphere to remove oxygen and water. Then, the temperature was rapidly increased to 300 °C. Keeping this temperature, a syringe with a long needle was used to inject the precursor solution seven times (respective volumes: 3.5, 4.0, 4.6, 5.6, 6.2, 7.1, 8.3), each with a delay of 10 min. After this, the 100-mL flask was quite full, and the reaction mixture was transferred to a 250-mL three-necked flask together with 30 mL of methanol. The solution was heated to 150 °C for ~30 min under an inert nitrogen atmosphere to remove oxygen, methanol, and water. The temperature was rapidly increased to 300 °C, and the injection of precursor solution was repeated twice (respective precursor volumes 9.6 and 10.1 mL); the temperature was kept at 300 °C for 10 min, eventually preparing nanoparticles of the desired size (reaction mixture volume ~82 mL), and the synthesis continued without decreasing the temperature by growing the inert shell of $NaYF_4$.

Shell growth

Under reflux, Y_2O_3 (1694 mg, 7.50 mmol) was dissolved in trifluoroacetic acid (12 mL) and water (12 mL) in a 250-mL three-necked flask. When dissolved, $NaHCO_3$ (1260 mg, 15.00 mmol) was added releasing CO_2 bubbles and dissolving rapidly. After removing the condenser, excessive trifluoroacetic acid and water were evaporated by heating at 110 °C in a fume hood (overnight). Together with sodium oleate (1644 mg, 5.4 mmol), the resulting white powder of trifluoroacetates was dissolved in oleic acid (45 mL, 40.3 g) and octadec-1-ene (45 mL, 35.5 g). This solution was diluted with 30 mL of methanol. The methanol together with oxygen and water were removed by heating at 110 °C under an inert nitrogen atmosphere for 20 min, resulting in a precursor solution. The precursor solution was enclosed in the flask by a silicon septum and kept under an inert atmosphere. To decrease the viscosity, the precursor solution was held at ~50 °C, which facilitated its injection into the reaction mixture. The precursor solution contained 0.17 mmol mL^{-1} of $Y(CF_3CO_2)_3$.

The shell was grown by gradually adding the precursor solution to the hot solution of grown nanoparticles from the previous step. A calculated amount of precursor solution was repeatedly injected by a syringe with a long needle (120 mm) without decreasing the temperature. The three additions were 8.0, 9.0, and 10.0 mL; the interval between the injections was 10 min. After the last injection, the temperature was kept at 300 °C for an additional 10 min, eventually preparing the desired nanoparticles (the volume of the reaction mixture was ~109 mL). Finally, the flask was cooled to room temperature. The resulting nanoparticles were precipitated by adding propan-2-ol (220 mL) and collected by centrifugation (1,000*g*, 10 min). The pellet was dispersed in cyclohexane (40 mL). After the

precipitation by methanol, the nanoparticles were dispersed in cyclohexane (40 mL) and slowly centrifuged (50*g*, 20 min) to separate coarse particles from the final product.

Supporting Note 4. Synthesis of $NaY_{0.80}Yb_{0.18}Tm_{0.02}F_4$ UCNPs capped with oleic acid

Seed nanoparticle synthesis

$YCl_3 \times 6\ H_2O$ (874 mg, 2.88 mmol), $YbCl_3 \times 6\ H_2O$ (251 mg, 0.648 mmol), and $TmCl_3 \times 6\ H_2O$ (27.6 mg, 0.072 mmol) were dissolved in 30 mL of methanol and added into a 250-mL three-neck round-bottom flask containing 27 mL (24.2 g) of oleic acid and 63 mL (49.7 g) of octadec-1-ene. The solution was heated to 170 °C under a protective nitrogen atmosphere until all volatile liquids were evaporated (~60 min). After the temperature had decreased to 50 °C, the nitrogen atmosphere was disconnected, and a solution of 533 mg (14.4 mmol) of NH_4F and 360 mg (9 mmol) of NaOH in 30 mL of methanol was added under intense stirring. The nitrogen atmosphere was reconnected, and the solution was stirred for 30 min. The temperature was carefully increased up to 150 °C, avoiding extensive boiling to ensure the evaporation of methanol. After that, the solution was rapidly heated at the rate of ~10 °C min^{-1}. At 290 °C, the heating was carefully adjusted to 300 °C within one or two minutes. The flask was kept under nitrogen flow at 300 °C (± 4 °C) for 90 min and then let to cool down to room temperature. The UCNPs were precipitated by adding 180 mL of propan-2-ol and collected by centrifugation (1,000*g*, 10 min). The precipitate was dispersed in 20 mL of cyclohexane. After adding 100 mL of methanol, the UCNPs precipitated rapidly without centrifugation. The precipitate was dispersed in 30 mL of cyclohexane and slowly centrifuged (50*g*, 20 min) to separate solid compounds from the final UCNPs.

Core growth

Under reflux, Y_2O_3 (1355 mg, 6.00 mmol), Yb_2O_3 (532 mg, 1.35 mmol), and Tm_2O_3 (57.9 mg, 0.15 mmol) were dissolved in trifluoroacetic acid (12 mL) and water (12 mL) in a 250-mL three-necked flask. When dissolved, $NaHCO_3$ (1260 mg, 15.00 mmol) was added, releasing CO_2 bubbles and dissolving rapidly. After removing the condenser, excessive trifluoroacetic acid and water were evaporated by heating at 110 °C in a fume hood (overnight). The resulting white powder of trifluoroacetates was dissolved in oleic acid (45 mL, 40.3 g) and octadec-1-ene (45 mL, 35.5 g). This solution was diluted with 30 mL of methanol. The methanol together with oxygen and water were removed by heating at 110 °C under the N_2 atmosphere for 20 min, resulting in a precursor solution. The precursor solution was enclosed in the flask by silicon septum and kept under an inert atmosphere. To decrease the viscosity, the precursor solution was kept at an elevated temperature (~50 °C), which facilitated its injection into the hot reaction mixture. The concentration of $Re(CF_3CO_2)_3$ in the precursor solution was 0.17 mmol mL^{-1} (Re is for Y, Yb, and Tm in molar percentages 80%, 18%, and 2%, respectively).

The dispersion of seed nanoparticles (205 mg) in cyclohexane was mixed with oleic acid (5.5 mL, 4.9 g), octadec-1-ene (17 mL, 13.4 g), and 20 mL of methanol in a 250-mL three-necked flask. The nanoparticles were grown by gradually adding the precursor solution to the solution of seed nanoparticles. The mixture was heated at 150 °C for ~30 min under the nitrogen atmosphere to remove oxygen and water. Then, the temperature was rapidly increased to 300 °C. Keeping this temperature, a syringe with a 120 mm long needle was used to inject the precursor solution ten times (respective volumes: 3.5, 4.0, 4.6, 5.6, 6.2, 7.1, 8.3, 9.6, 11.1, and 12.8 mL), each with a delay of 10 min. After the last addition, the temperature was kept at 300 °C for 10 min eventually preparing nanoparticles of the desired size (reaction mixture volume ~95 mL). The resulting nanoparticles were precipitated by adding propan-2-ol (190 mL) and collected by centrifugation (1,000*g*, 10 min). The pellet was dispersed in cyclohexane (40 mL). After the precipitation by methanol, the nanoparticles were dispersed in cyclohexane (40 mL) and slowly centrifuged (50*g*, 20 min) to separate coarse particles from the final product.

Supporting Note 5. Coating the nanoparticles with carboxylated silica

Photon-upconversion nanoparticles (UCNPs) were silica-coated and carboxylated by a reverse microemulsion method. UCNPs were diluted in cyclohexane with Igepal CO-520, tetraethyl orthosilicate, and stirred with high intensity for 10 min. The microemulsion was created after adding an aqueous ammonium hydroxide (12% w/v). The resulting mixture was slowly stirred overnight. Another volume of tetraethyl orthosilicate was added and the microemulsion was slowly stirred for 4 hours. Carboxyethylsilanetriol sodium salt (25 % w/v in water) was added and the turbid emulsion was sonicated for 15 min and further stirred for 60 min. Carboxylated UCNPs were extracted by adding 500 µL of dimethylformamide and washed four times with 4.5 mL of acetone and three times with 4.5 mL of water. Carboxylated UCNPs were stored in a refrigerator (4 °C) as an aqueous dispersion in a concentration of 10 mg mL^{-1}.

Table S1: Preparation of carboxylated silica shell ($NaY_{0.80}Yb_{0.18}Tm_{0.02}F_4$@carboxylated silica, and $NaY_{0.80}Yb_{0.18}Er_{0.02}F_4/NaYF_4$@carboxylated silica).

Coated nanoparticles	$NaY_{0.80}Yb_{0.18}Tm_{0.02}F_4$	$NaY_{0.80}Yb_{0.18}Er_{0.02}F_4/NaYF_4$
UCNP (mg)	30	30
Cyclohexane (mL)	15	7.5
Igepal CO-520 (mg)	900	600
Tetraethyl orthosilicate I (µL)	51	34
NH_4OH (12% w/v in water, µL)	113	75
Tetraethyl orthosilicate II (µL)	13	8.5
Carboxyethylsilanetriol sodium salt (25% w/v in water, µL)	26	17

Supporting Note 6. Preparation of biotinylated bovine serum albumin

BSA (1000 mg, 15 µmol) was dissolved in 10 mL of $NaHCO_3$ solution (1 mol L^{-1}). Biotin-NHS (10 mg, 30 µmol) was dissolved in 250 µL of DMF and mixed with the dispersion of BSA. After reacting for four hours at 24 °C, the dispersion was dialyzed (14 kDa membrane) against 1000 mL of water. The first and the second dialysis were for 24 h. The third was over a weekend. The biotinylated BSA was analyzed by matrix-assisted laser desorption ionization and the average number of 1.4 biotin residues per single BSA molecule was estimated. The biotinylated BSA was aliquoted and stored in the freezer.[1]

Supporting Note 7. Preparation of biotinylated nanoparticles (UCNP-Tm-Biotin)

The carboxyl groups of nanoparticles were first activated by EDC and sulfo-NHS by mixing 2 mg of $NaY_{0.80}Yb_{0.18}Tm_{0.02}F_4$@carboxylated silica and 333 µL of MES buffer containing EDC (4 mg mL^{-1}), and sulfo-NHS (2 mg mL^{-1}).[2] After 15 min at laboratory temperature, the nanoparticles were rapidly centrifuged (1 min, 7000*g*). The pellet was dispersed in 333 µL of MES buffer containing bovine serum albumin (16 mg mL^{-1}) and biotinylated bovine serum albumin (4 mg mL^{-1}). This mixture was reacted for 120 min at laboratory temperature in a rotator. The biotinylated nanoparticles were five times washed with 500 µL of MOPST buffer with centrifugation (30 min, 7000*g*) to collect the bioconjugated nanoparticles after each wash. Finally, the pellet after the last washing was dispersed into 500 µL of MOPST buffer, which was supplemented with NaN_3 to a concentration of 0.05% (w/v).

Supporting Note 8. Preparation of streptavidin-conjugated nanoparticles (UCNP-Er-Streptavidin)

The carboxyl groups of nanoparticles were first activated by EDC and sulfo-NHS by mixing 2 mg of $NaY_{0.80}Yb_{0.18}Er_{0.02}F_4/NaYF_4$@carboxylated silica and 333 µL of MES buffer containing EDC (4 mg mL^{-1}), and sulfo-NHS (2 mg mL^{-1}).[2] After 15 min at laboratory temperature, the nanoparticles were rapidly centrifuged (1 min, 7000*g*). The pellet was dispersed in 333 µL of MES buffer containing bovine serum albumin (16 mg mL^{-1}) and streptavidin (4 mg mL^{-1}). This mixture was reacted for 120 min at laboratory temperature. The protein-coated nanoparticles were five times washed with 500 µL of MOPST buffer with centrifugation (30 min, 7000*g*) to collect the bioconjugated nanoparticles after each wash. Finally, the pellet after the last washing was dispersed into 500 µL of MOPST buffer, which was supplemented with NaN_3 to a concentration of 0.05% (w/v).

Supporting Note 9. Nanomaterial characterization

Estimating nanoparticle hydrodynamic diameters by dynamic light scattering

Dynamic light scattering was used for measuring the nanoparticle hydrodynamic diameters. The diluted sample was dispensed into a glass cuvette with a 10 mm optical path and inserted into the Zetasizer Nano ZS (Malvern Panalytical) and data were collected with a standard measurement setting.

Transmission electron microscopy

The oleic acid-capped nanoparticles were diluted to 1 mg mL^{-1} in cyclohexane to prepare samples for transmission electron microscopy (TEM). The volume of 6 µL was dropped on a TEM grid (Quantifoil Micro Tools, copper R 2/1), and the excess liquid was absorbed by a paper tissue, and dried freely on air.

Estimating the mass concentration of nanoparticle dispersion

For estimating nanoparticle mass concentrations, the volume of 250 µL of oleic acid-capped UCNPs dispersed in cyclohexane was filled into a glass vial. The vial was placed firstly on a heater to evaporate cyclohexane and secondly for 90 min into a furnace at 550 °C. The mass of nanoparticles was then used for calculating the mass concentration. The same procedure was also utilized for estimating the concentration of the silica-coated UCNPs.

Estimating molar concentration of bioconjugated UCNPs

An absolute counting method in an anisotropically collapsed agarose gel was used for estimating the number concentration of bioconjugated UCNPs.[3] The low melting point agarose (100 mg) was melted in 10 mL of boiling water. Dissolved agarose was tempered at 35 °C before mixing with bioconjugated nanoparticles diluted with water. For casting the microlayer of agarose gel, the microscope slide was attached with two pieces of plastic tape (thickness ~40 µm) with approximately 5 mm space between them. The agarose-dispersed sample of nanoparticles (10 µL) was applied to the space between plastic tapes and covered with a glass (thickness 1 mm). The glass slides with the plastic tape between them were pressed together by two binder clips. The glass assembly was placed in the fridge for 15 min to solidify the gel. Then, the cover glass was slipped away, and the thin layer of agarose gel was dried on air (~5 min). The dried (collapsed) gel was dropped with immersion oil and covered with a cover glass (thickness 170 µm). The nanoparticles were imaged as diffraction-limited spots with an epiphoton-upconversion microscope, counted, and the concentration was calculated.[3] The histograms of spot intensities were used for evaluating the content of non-aggregated nanoparticles.

Specificity assay of UCNP-Tm-Biotin

The wells were coated overnight at 4 °C with streptavidin (5 µg mL^{-1} in coating buffer, 100 µL per well, three replica wells). For negative control, the coating buffer without streptavidin was used (three replica wells). Wells were twice washed with MOPST buffer and blocked with assay buffer for 60 min at 24

°C. The blocked wells were twice washed with MOPST buffer and then loaded with diluted UCNP-Tm-Biotin (100 pmol L^{-1}) in the assay buffer for 60 min at 24 °C. After five times washing with MOPST buffer, and once with water, the plate was scanned for photon-upconversion luminescence (764-822 nm for Tm^{3+} emission).

Specificity assay of UCNP-Er-Streptavidin

The wells were coated overnight at 4 °C with biotinylated bovine serum albumin (5 µg mL^{-1} in coating buffer, 100 µL per well, three replica wells). For negative control, the coating buffer without streptavidin was used (three replica wells). Wells were twice washed with MOPST buffer and blocked with assay buffer for 60 min at 24 °C. The blocked wells were twice washed with MOPST buffer and then loaded with diluted UCNP-Er-Streptavidin (100 pmol L^{-1}) in the assay buffer for 60 min at 24 °C. After five times washing with MOPST buffer, and once with water, the plate was scanned for photon-upconversion luminescence (512-556 nm for Er^{3+} emission).

Native gel electrophoresis of bioconjugated UCNPs

Before native gel electrophoresis, the following dispersions were prepared:

Dispersion	Composition
A1	UCNP-Er-Streptavidin (1 nmol L^{-1}), biotin (50 µmol L^{-1}), DMF (0.1% v/v); in acetate buffer
A2	UCNP-Tm-Biotin (1 nmol L^{-1}), biotin (50 µmol L^{-1}), DMF (0.1% v/v); in acetate buffer
A3	UCNP-Er-Streptavidin (1 nmol L^{-1}), DMF (0.1% v/v); in acetate buffer
A4	UCNP-Tm-Biotin (1 nmol L^{-1}), DMF (0.1% v/v); in acetate buffer

After 15 min incubation at 24 °C, the following dispersions were prepared:

Dispersion	Composition
B1	Dispersions A1 (20 µL) and A2 (20 µL) were mixed.
B2	Dispersions A3 (20 µL) and A4 (20 µL) were mixed 1:1 (v/v)

After another 70 min of incubation of dispersions A3, A4, B1, and B2 at 24 °C, the following dispersions were prepared:

Dispersion	Composition
C1	Dispersion A3 (10 µL) was mixed with 10% (w/v) glycerol in MOPST buffer (90 µL).
C2	Dispersion A4 (10 µL) was mixed with 10% (w/v) glycerol in MOPST buffer (90 µL).
C3	Dispersion B2 (10 µL) was mixed with 10% (w/v) glycerol in MOPST buffer (90 µL).
C4	Dispersion B1 (10 µL) was mixed with 10% (w/v) glycerol in MOPST buffer (90 µL).

Dispersions C1, C2, C3, and C4 were loaded on 1% agarose gel in two replicated pockets for each dispersion (18 µL per pocket). The MOPST buffer served as an electrophoresis buffer. The electrophoresis ran at a constant current of 10 mA for 240 min (Owl™ C2-S Micro Electrophoresis System). The developed gel was scanned for photon-upconversion at two wavelengths: 512-556 nm (Er^{3+} emission) and 764-822 nm (Tm^{3+} emission). The resolution of the scan was 120 µm.

Supporting Note 10. Manufacturing the polydimethylsiloxane microfluidic chip

Simple microchannel architecture with a depth of 30 µm and a width of 100 µm was designed in AutoCAD® software (Autodesk 2015) and photolithographically transferred on negative photoresist SU-8 3050 (MicroChem Corp., USA) using a high-resolution laser printer MicroWriter ML3 (Durham Magneto Optics Ltd, UK). SYLGARD 184 kit (Dow Corning, USA) was used for polydimethylsiloxane (PDMS) chip fabrication with a 1/10 (w/w) ratio of curing agent and carried out using standard procedure. The mold was finally bonded upon oxygen plasma treatment to a glass slide with a thickness of 170 µm. To prevent cracks, the thin glass substrate was glued to a protective plastic frame by using an epoxy resin. The set of silica capillaries with a length of 50 cm and 100 µm inner diameters (Molex, USA) were used for fluid connecting. The ends of capillaries (~1.5 cm length) were tightly fitted with polytetrafluoroethylene tubes, which served as robust connectors for the PDMS chip (1/16" outer diameter and 0.01 inner diameter; Alltech).

Supporting Note 11. Instrumentation

Instrument configaration

A laboratory-built epiphoton-upconversion microscope was accommodated for massively parallel spectroscopy (MPS) of photon-upconversion nanoparticles. Oil immersion microscope objective CFI Plan Apo Lambda was utilized (magnification 60×, NA 1.40; Nikon). For detecting photon-upconversion luminescence, an optical filter (875 nm short-pass; Edmund Optics), and a dichroic mirror (900 nm short-pass; Thorlabs) were used. To select only long-wavelength emission, a 600 nm long-pass filter was added (Edmund Optics). The spectra of single emission bands were recorded by replacing the 600 nm long-pass filter with band-pass filters (475 ± 25 nm, 550 ± 25 nm, 650 ± 25 nm, or 800 ± 25 nm, Edmund Optics). An optical fiber (105 µm diameter) connected a laser diode for UCNP excitation (976 nm, 10 W fiber-couplet laser diode module, Roithner Lasertechnik). The end of the optical fiber was equipped with a collimator (4.3 mm focal length; Thorlabs) projecting the laser beam into the microscope objective. Before reflecting on the dichroic mirror, the laser beam was passed through a long-pass optical filter (925 nm cut-off wavelength; Thorlabs). The excitation power of the laser was measured with a microscope slide thermal sensor from Thorlabs dropped with immersion oil. For creating the MPS images on the camera image sensor (sCMOS camera Zyla 5.5, Andor Technology - Oxford Instruments), a tube-lens with 200 mm focal length (Thorlabs) was used resulting in 60× magnification. To provide spectra dispersion, a laboratory-polished prism made of poly(methyl methacrylate) was inserted between the tube lens and the camera (60 mm in front of the camera sensor, see the Figure 1 in the main text for prism dimensions). The camera was shifted slightly from the optical axis to compensate for the prism.

Characterization of the excitation laser and luminescence saturation of UCNPs

The illumination of the focal plane of the microscope by excitation laser was observed as a backscattering from a microlayer of silica submicron particles (size ~500 nm) embedded in a dried agarose gel (Figure S1A), which was inserted into the focal plane of the microscope objective. The intensity of the 976 nm laser was reduced to only 0.5 mW and the backscattering was observed (Figure S1B) when all optical filters between the dichroic mirror and the camera were removed revealing the Gaussian profile of the illumination (Figure S1C). By integrating the backscattering intensity values in Figure S1B, it was estimated that 70% of the laser power is distributed within the MPS observation area (diameter111 µm, area 9.69×10^{-5} cm^2). For all MPS experiments, the power of the excitation beam transmitted through the microscope objective was 1.3 W. Therefore, the average intensity of the excitation laser within the observation area was: $70 / 100 \times 1.3 / 9.69 \times 10^{-5} = 9.4$ kW cm^{-2}.

For characterizing the saturation of photon-upconversion emission within the observation area (Figure S1D-O), submicron layers containing a high density of UCNPs ($NaY_{0.80}Yb_{0.18}Tm_{0.02}F_4$@carboxylated

silica, or $NaY_{0.80}Yb_{0.18}Er_{0.02}F_4/NaYF_4$@carboxylated silica) were inserted into the focal plane of the microscope objective. The photon-upconversion emission was imaged with a variable power of the excitation laser (0.28–1.9W) and variable band-pass filters in front of the camera (550 ± 25 nm, or 650 ± 25 nm for Er^{3+} emission, and 475 ± 25 nm, or 800 ± 25 nm for Tm^{3+} emission). The profiles of emission intensities were constructed by using these images. These profiles show high enough emission from nanoparticles located by the edge of the detection area.

Additionally, the average intensities from four squared areas (11 µm × 11 µm) were plotted as a function of the excitation power (0.28–1.9W). These results show that the emission at bands 550 ± 25 nm, 650 ± 25 nm, or 800 ± 25 nm at 1.3 W excitation power was partially saturated (the increment of emission intensity was decreasing with increasing laser power). The saturation was the most apparent for the near-infrared emission (800 ± 25 nm). The blue emission (475 ± 25 nm) was less saturated and increased approximately linearly with the power of the excitation laser.

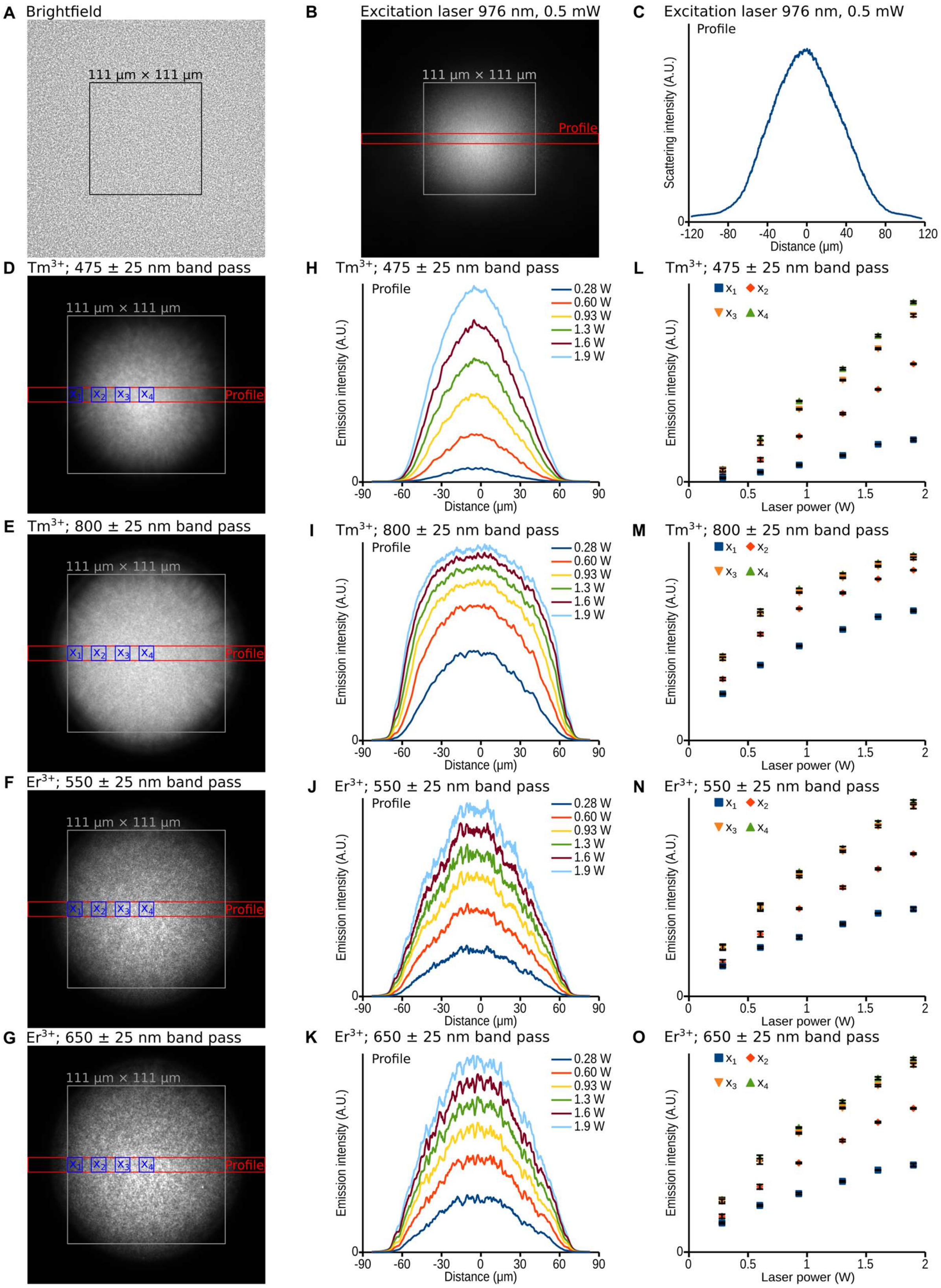

Figure S1. The characteristics of illumination and saturation of photon-upconversion emission. **A-C**, A microlayer of silica submicron particles. **A**, In the bright-field mode, a homogeneous layer of silica particles was observed (particle size ~500 nm, illuminated with white light-emitting diode). **B**, After removing all optical filters between the dichroic mirror and the camera, the backscattering of the excitation laser (wavelength 976 nm, power 0.5 mW) from silica particles was observed. **C**, A profile of the backscattering, as indicated in panel B, was plotted and revealed approximately Gaussian shape.

D-G, The micrographs of photon-upconversion emission from the dense submicron layers of UCNPs (excitation laser power 1.3 W, the same was used for all MPS experiments). Layers of either, **D,E**, $NaY_{0.80}Yb_{0.18}Tm_{0.02}F_4$@carboxylated silica, or, **F,G**, $NaY_{0.80}Yb_{0.18}Er_{0.02}F_4/NaYF_4$@carboxylated silica UCNPs were imaged by using 875 nm short-pass filter with additional band-pass filters, as indicated in the panels. **H-K**, The profiles of emission intensity at variable power of the excitation laser. Emission profiles were constructed from micrographs of dense submicron layers of UCNPs, which were recorded at a gradually increasing power of the excitation laser (0.28–1.9 W). The H-K profiles were constructed over rectangular areas as indicated in panels D-G, respectively. **L-O**, Emission intensity from selected regions of dense submicron layers of UCNPs as a function of the excitation power. Micrographs were recorded at a variable power of the excitation laser (0.28, 0.60, 0.93, 1.3, 1.6, and 1.9 W). Four measuring regions were selected (size 11 μm × 11 μm), as indicated in panels D-G, respectively. Average emission intensities from these regions were plotted against the power of the excitation laser. The square area with the edge length of 111 μm in panels A,B,D-G indicates the size of MPS images.

Supporting Note 12. Processing MPS images

FIJI-ImageJ was used for visualizing and manual image evaluation. A laboratory-developed software utilizing a convolutional neural network was used for automatic MPS processing (implemented in the Python programming language using the Keras deep learning interface). The MPS images were recorded in 16-bit pixel depth providing intensity values from ~100 to 65535. Before neural network analysis, these micrographs were logarithmized with the base of two and divided by a factor of 16 resulting in values from ~0.4 to 1.0. To perform the localization of spectra, we have used a convolutional neural network with a U-net architecture (Figure S2). The network was trained on simulated data. The spots were simulated as two-dimensional Gaussian peaks with a Poisson noise. To introduce the Poisson noise, the simulated intensity was replaced with a random sample from a Poisson distribution having the mean value equivalent to that simulated intensity. The capability for detecting the double spots was trained by simulating this pattern in the training data set (the Gaussian peaks were grouped into pairs). The simulated peaks were superimposed to real MPS images to introduce realistic camera noise, background, and the patterns of nanoparticles, which are out of the focal plane. These "background" images were similar to Figure 3P,Q in the main text without dual spot patterns. A mask indicating the positions of simulated double spots was generated for each image. The size of simulated images was 256 px × 256 px, 1 to 50 double spots were simulated per image, and the training set contained 6000 image-mask pairs with a 25% validation split. The trained network was tested manually by evaluating real MPS images. Once trained, U-net processed the logarithmized MPS images and returned maps of spectra localizations, which were converted to binary masks by thresholding. The binary masks of double spot localizations were used for counting. The U-net processed micrographs of size 256 px × 256 px. Larger images were processed as fragments of this size and the counting was made after concatenating the localization masks. To ease manual inspection, green rectangles marked double spots in the overlay with original images.

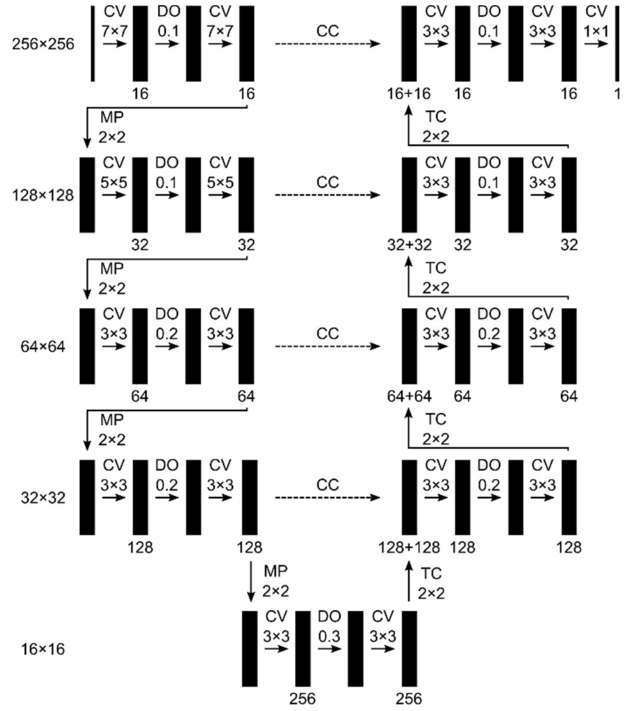

Figure S2. The scheme of utilized U-net. The U-net is composed of convolution (CV), drop out (DO), max pooling (MP), transposed convolution (TC), and concatenation (CC) layers. In the first part, the network successively reduces the size of the input image (256 px × 256 px) to a set of 256 feature maps of reduced size (16 px × 16 px). In the second part, the map of double spot localizations is built (256 px × 256 px).

Supporting Note 13. Competitive assay of biotin on microtiter plates

Assay of biotin with UCNP-Tm-Biotin

The plates were coated overnight at 4 °C with streptavidin (0.1 µg mL^{-1} in coating buffer, 100 µL per well). Wells were twice washed with MOPST buffer and blocked with assay buffer for 120 min at 24 °C (300 µL per well). A dilution series of biotin in the assay buffer containing UCNP-Tm-Biotin (10 pmol L^{-1}) was prepared. The blocked wells were four times washed with MOPST buffer, loaded with biotin dilution series (200 µL per well), and incubated for 45 min at 24 °C. After four times washing with MOPST buffer, and once with water, the plate was scanned for the intensity of photon-upconversion luminescence (764-822 nm for Tm^{3+} emission). The limit of detection was 68 pmol L^{-1} and IC_{50} was 140 pmol L^{-1} (Figure S3A, the limit of detection and IC_{50} as described in the main text for the biotin assay with the luminescence intensity as an analytical signal; eq. 1, eq. 2 in the main text).

Assay of biotin with UCNP-Er-Streptavidin

The plates were coated overnight at 4 °C with biotinylated bovine serum albumin (2.5 µg mL^{-1} in coating buffer, 100 µL per well). Wells were twice washed with MOPST buffer and blocked with assay buffer for 120 min at 24 °C (300 µL per well). A dilution series of biotin in the assay buffer containing UCNP-Er-Streptavidin (10 pmol L^{-1}) was prepared and incubated for 20 min at 24 °C. The blocked wells were four times washed with MOPST buffer, loaded with biotin dilution series (200 µL per well), and incubated for 45 min at 24 °C. After four times washing with MOPST buffer, and once with water, the plate was scanned for the intensity of photon-upconversion luminescence (512-556 nm for Er^{3+} emission). The limit of detection was 17 pmol L^{-1} and IC_{50} was 58 pmol L^{-1} (Figure S3B, the limit of detection and IC_{50} as described in the main text for the biotin assay with the luminescence intensity as an analytical signal; eq. 1, eq. 2 in the main text).

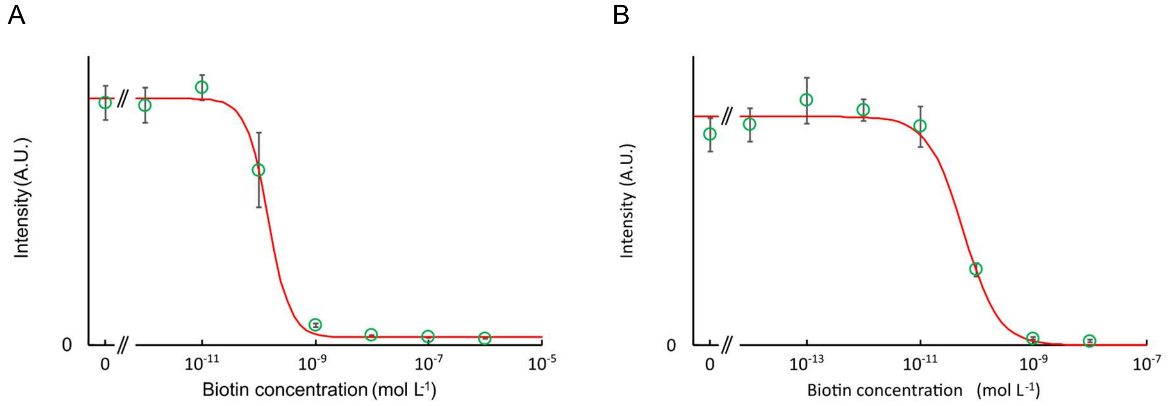

Figure S3. Competitive assay of biotin on a microtiter plate by using either, **A**, UCNP-Tm-Biotin, or, **B**, UCNP-Er-Streptavidin.

Micrographs

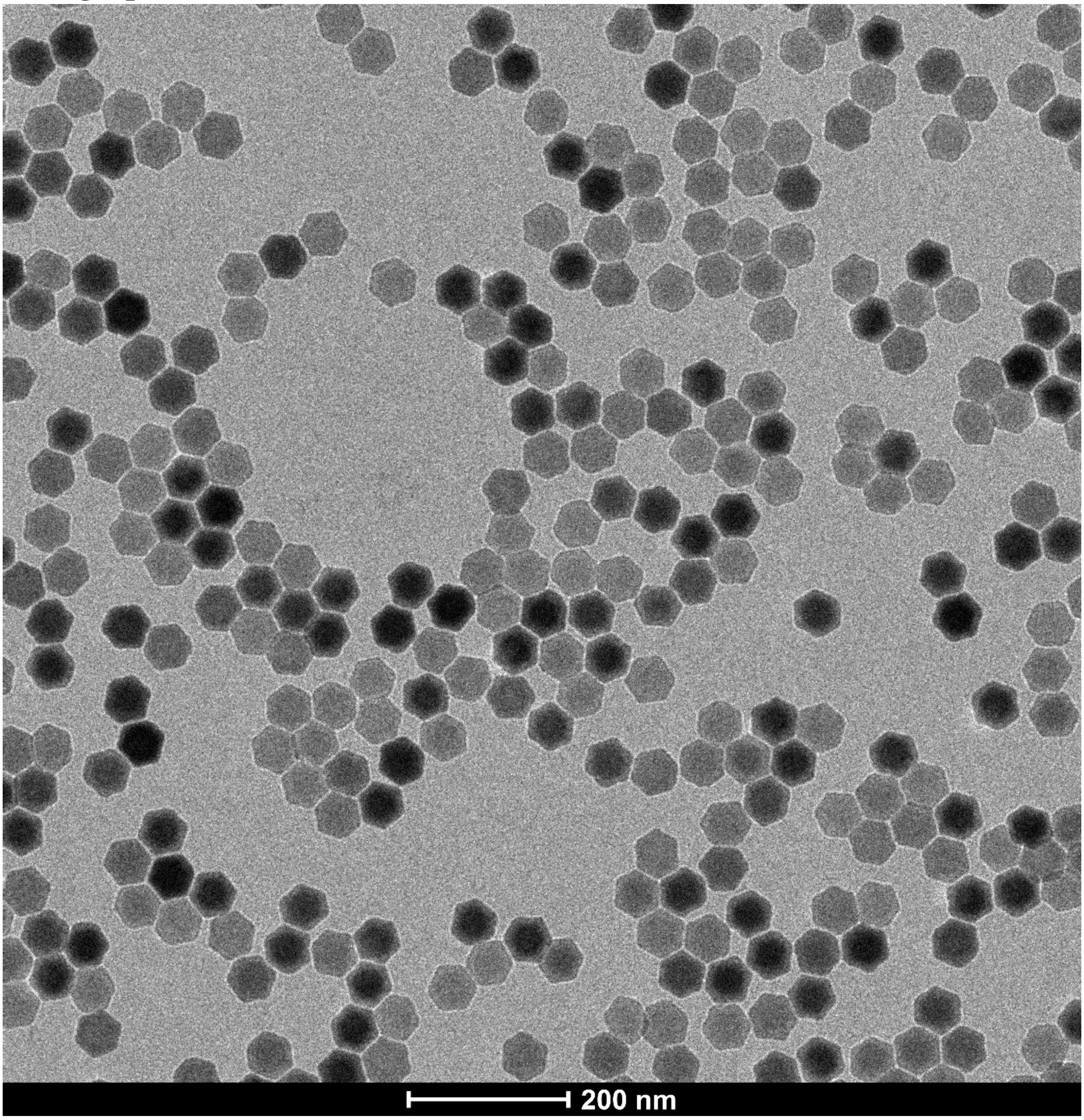

Figure S4. Full frame of Figure 2, panel A.

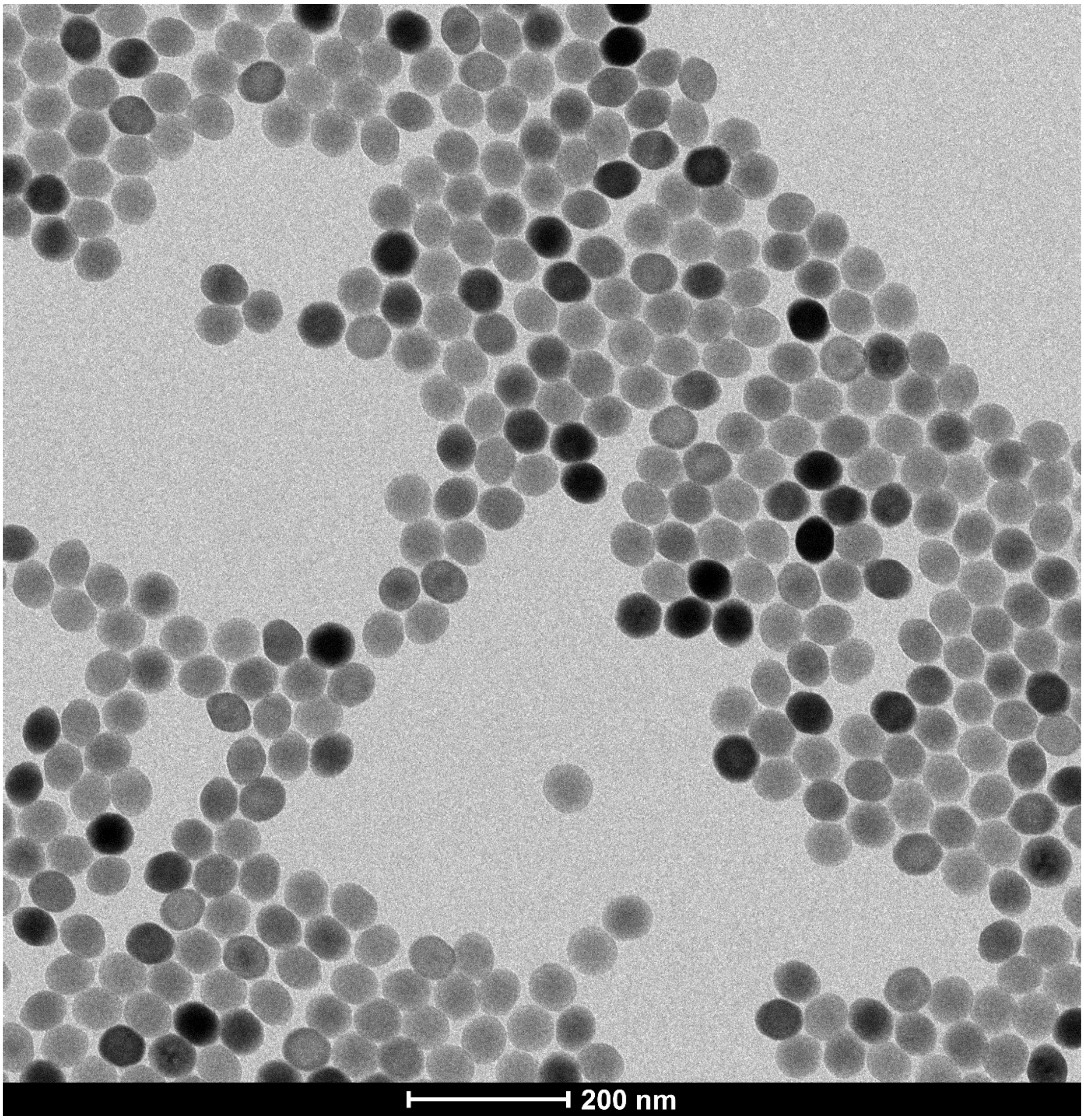

Figure S5. Full frame of Figure 2, panel B.

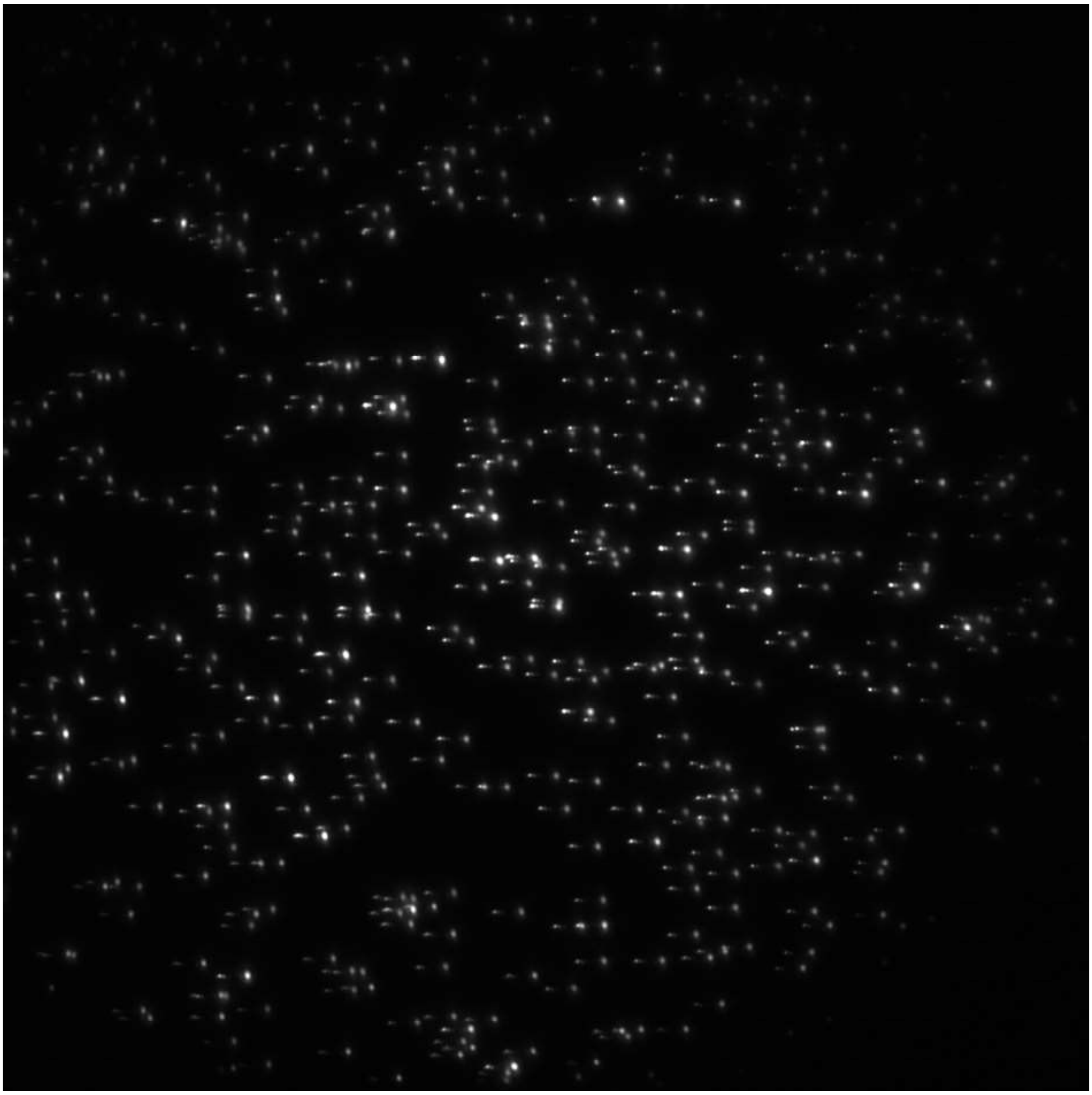

Figure S6. Full frame of Figure 3, panel a (image size 1024 px × 1024 px; 111 µm × 111 µm).

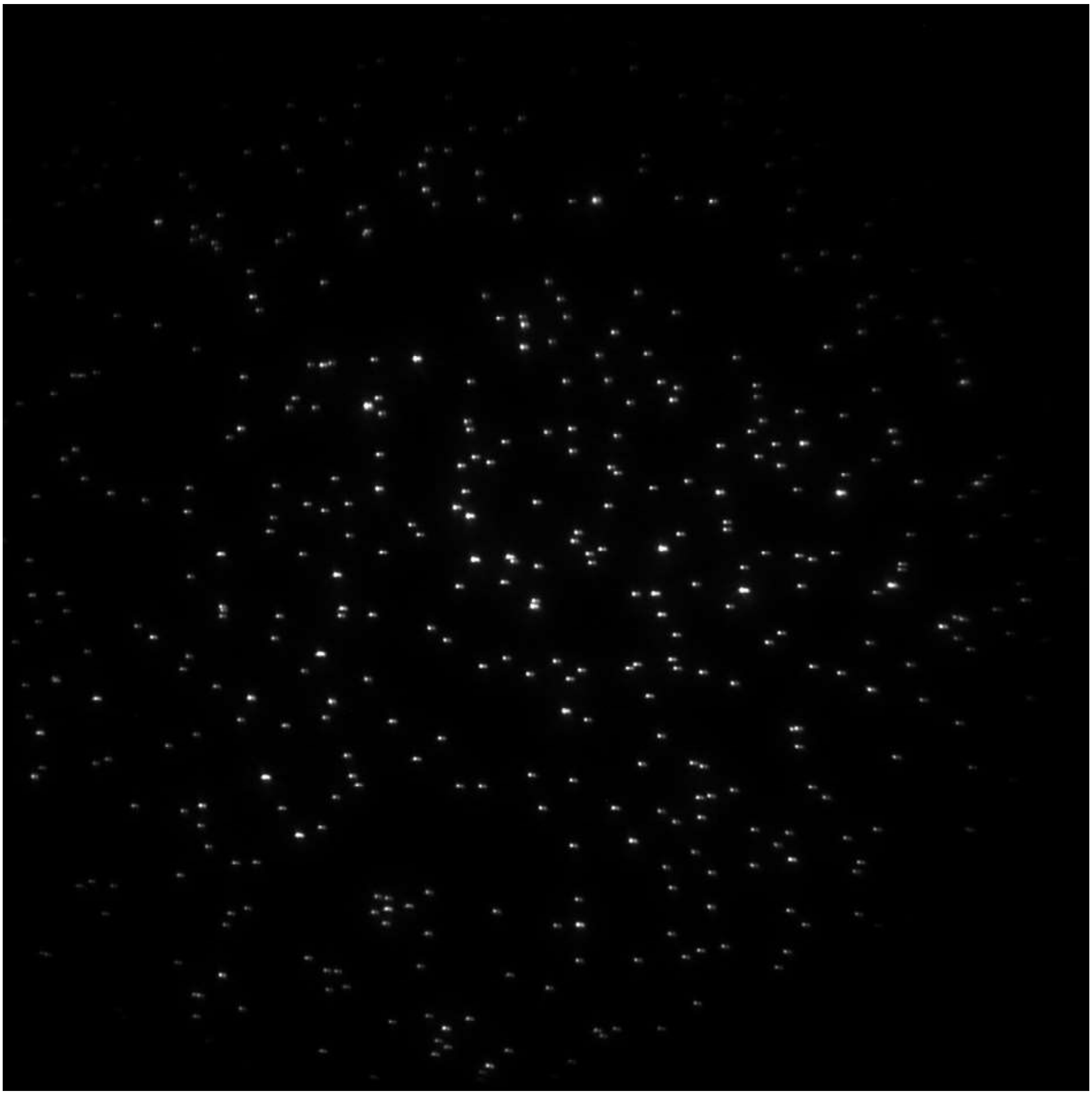

Figure S7. Full frame of Figure 3, panel b (image size 1024 px × 1024 px; 111 µm × 111 µm).

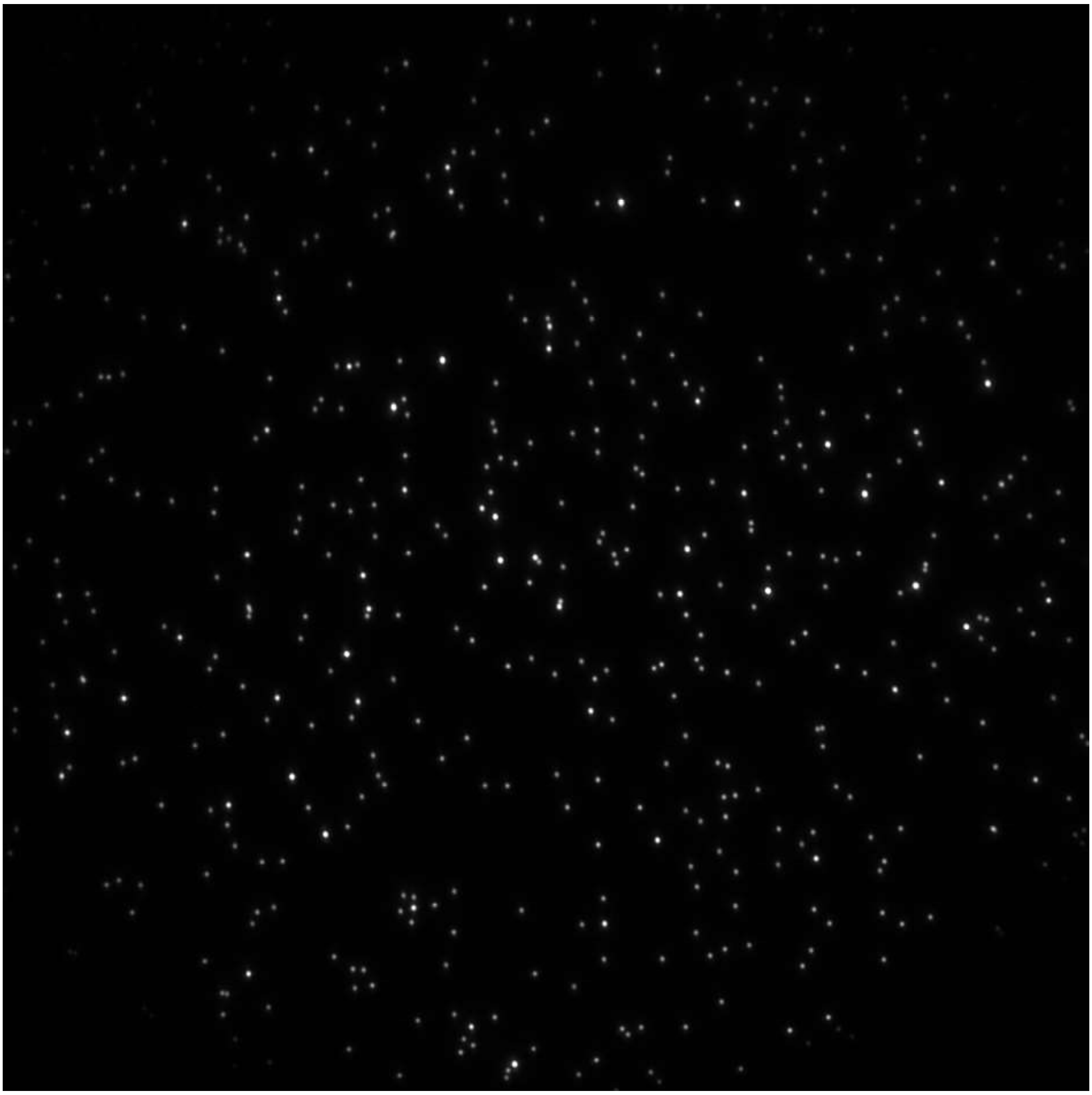

Figure S8. Full frame of Figure 3, panel c (image size 1024 px × 1024 px; 111 µm × 111 µm).

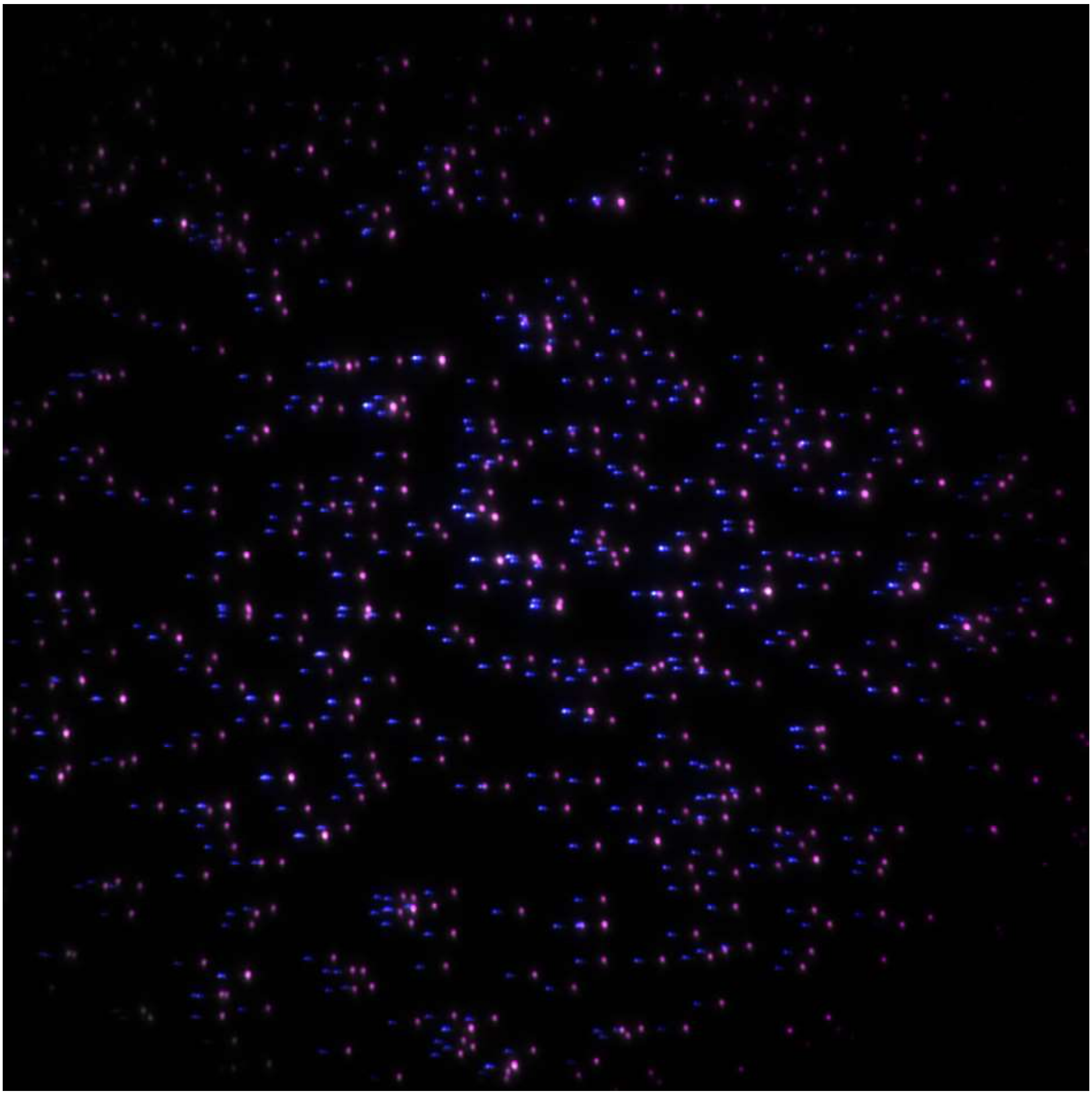

Figure S9. Full frame of Figure 3, panel d (image size 1024 px × 1024 px; 111 µm × 111 µm).

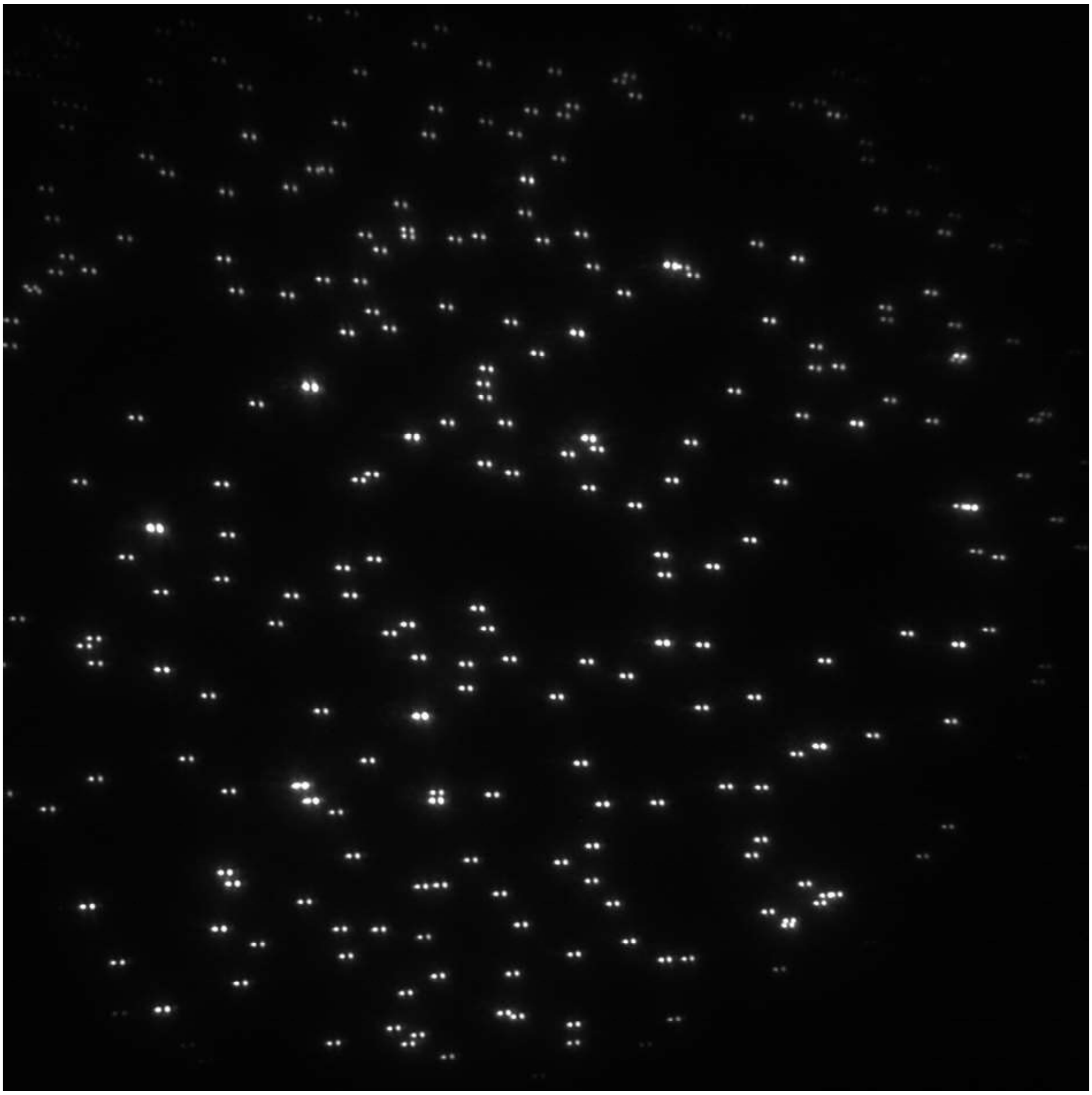

Figure S10. Full frame of Figure 3, panel f (image size 1024 px × 1024 px; 111 μm × 111 μm).

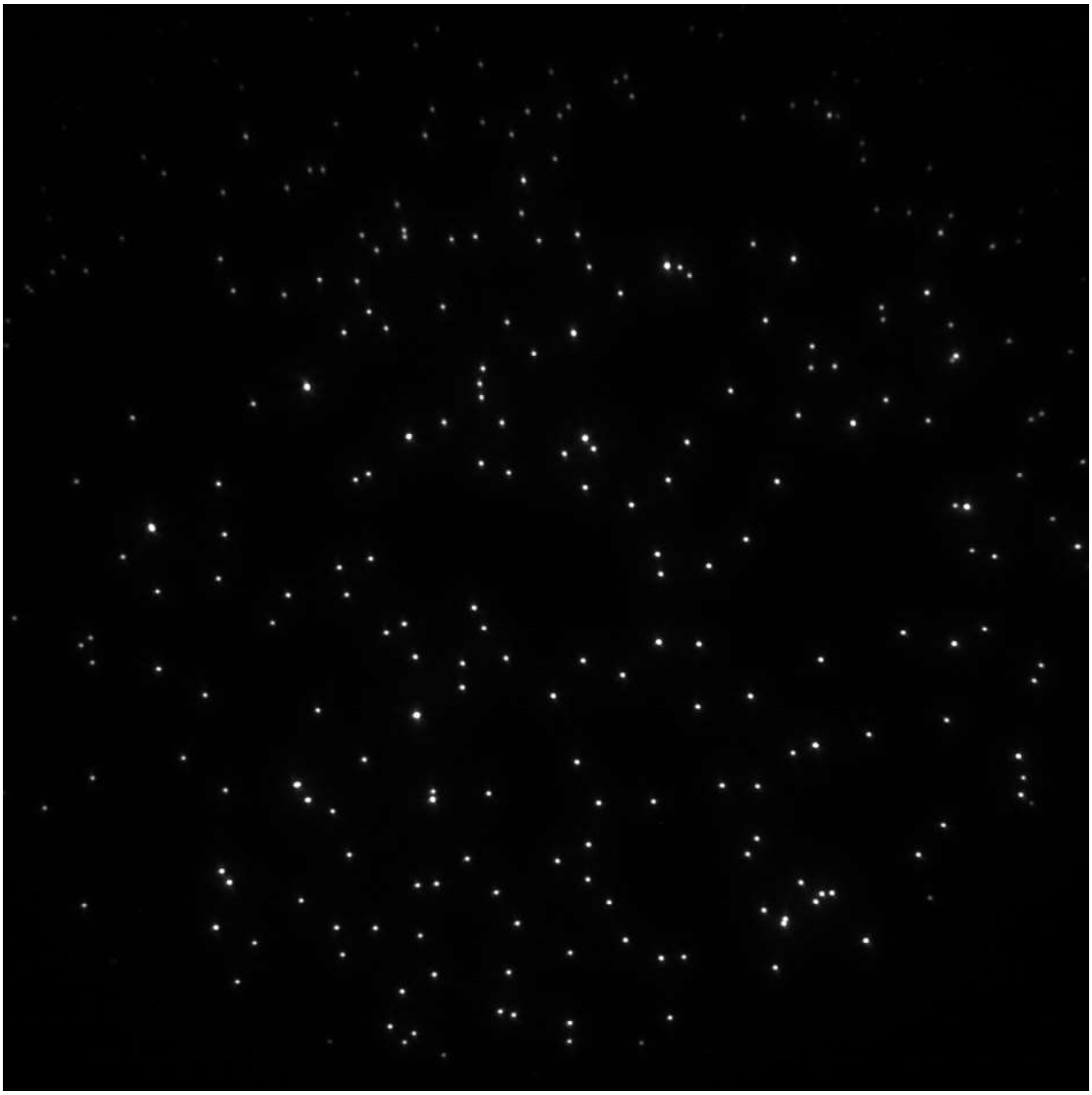

Figure S11. Full frame of Figure 3, panel g (image size 1024 px × 1024 px; 111 µm × 111 µm).

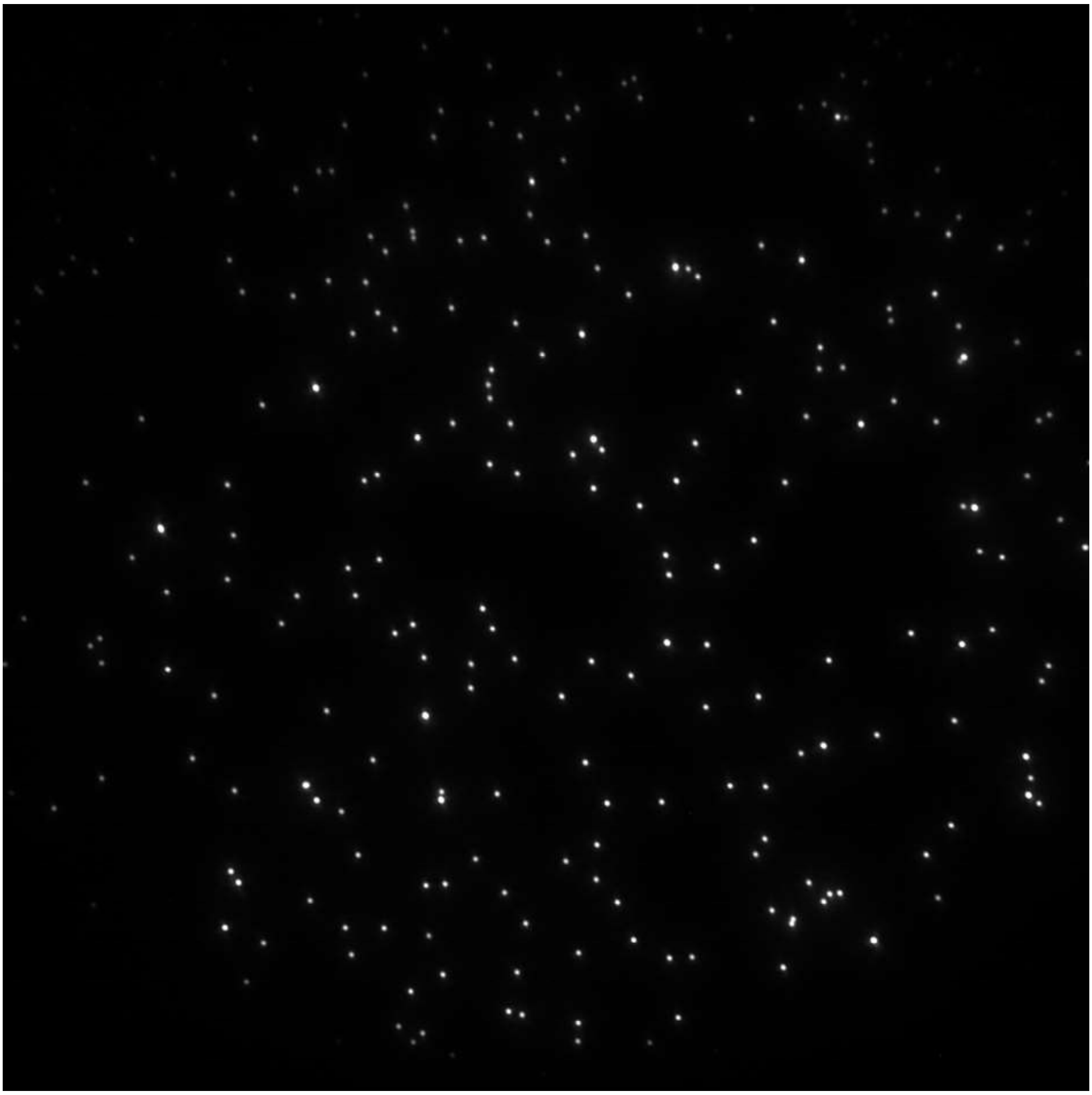

Figure S12. Full frame of Figure 3, panel h (image size 1024 px × 1024 px; 111 µm × 111 µm).

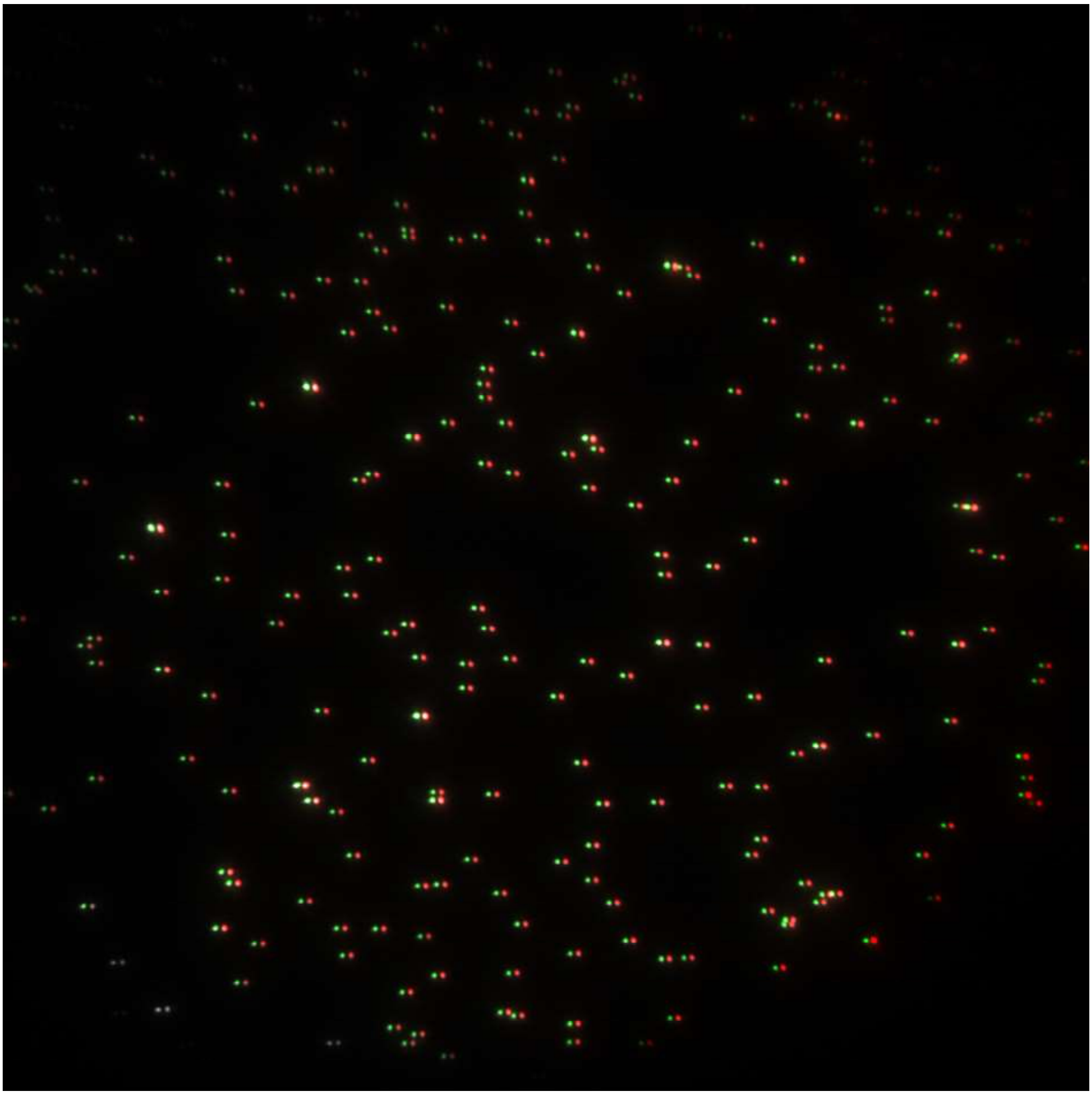

Figure S13. Full frame of Figure 3, panel i (image size 1024 px × 1024 px; 111 µm × 111 µm).

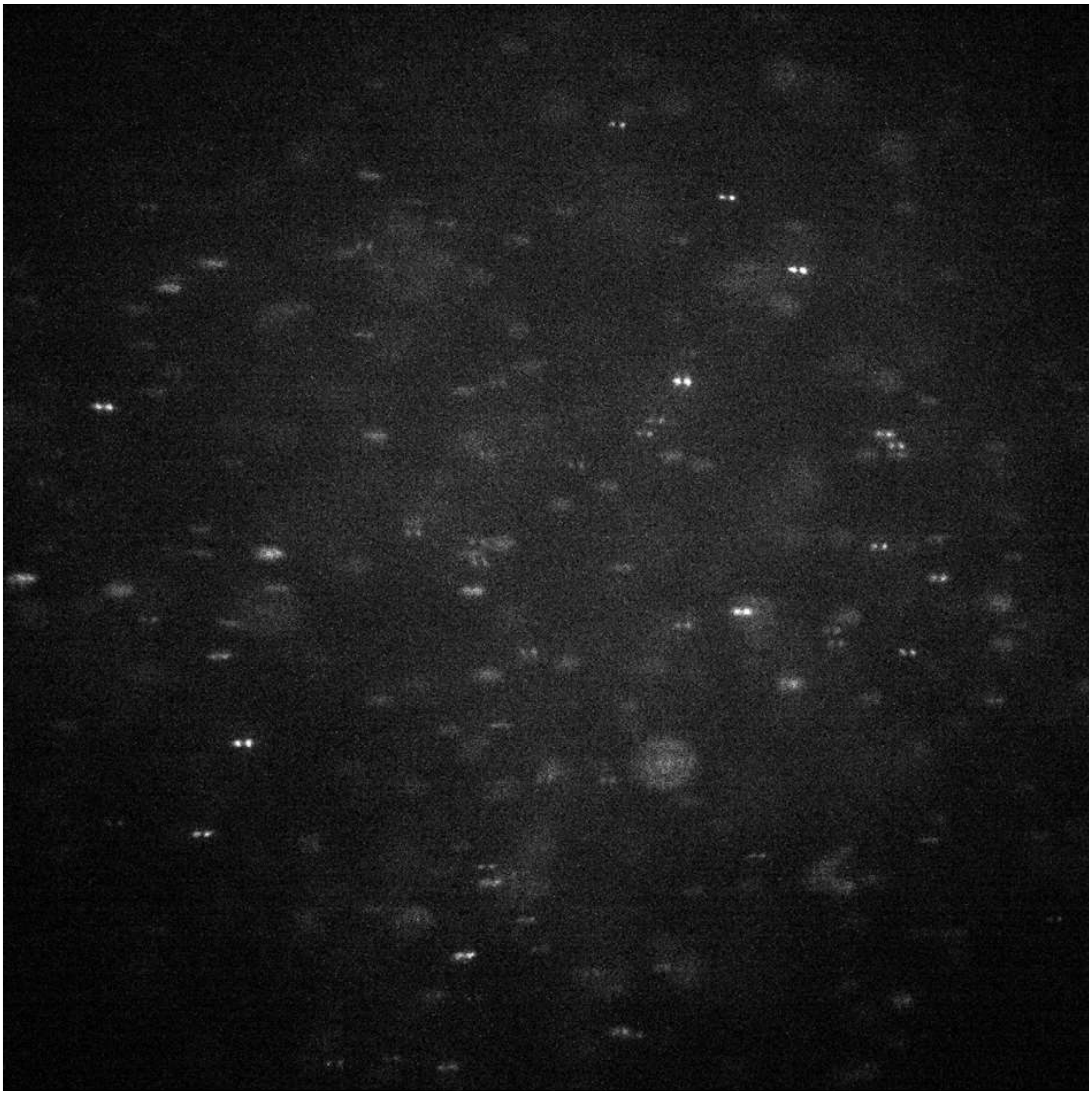

Figure S14. Full frame of Figure 3, panel l (image size 1024 px × 1024 px; 111 µm × 111 µm).

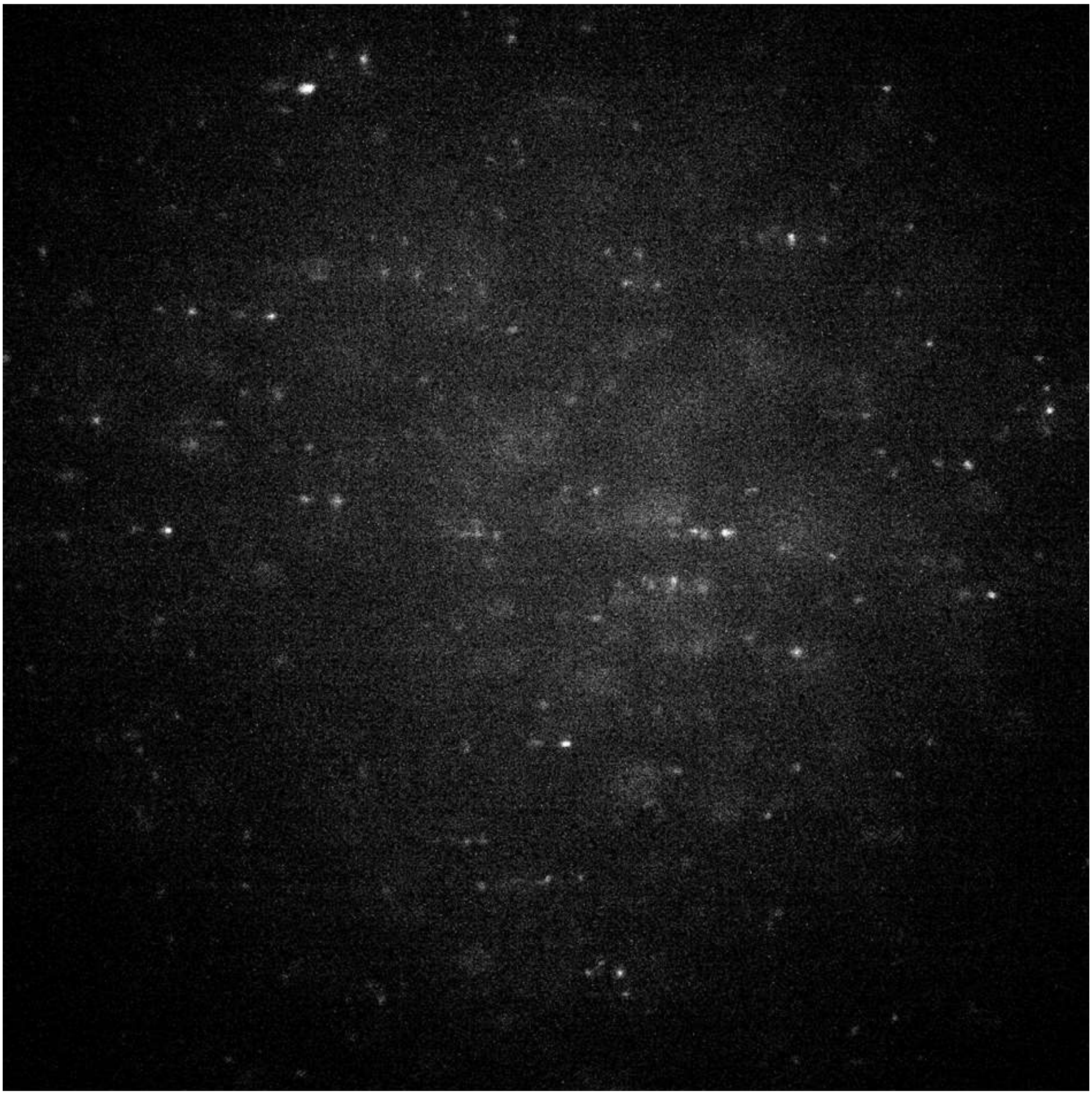

Figure S15. Full frame of Figure 3, panel m (image size 1024 px × 1024 px; 111 µm × 111 µm).

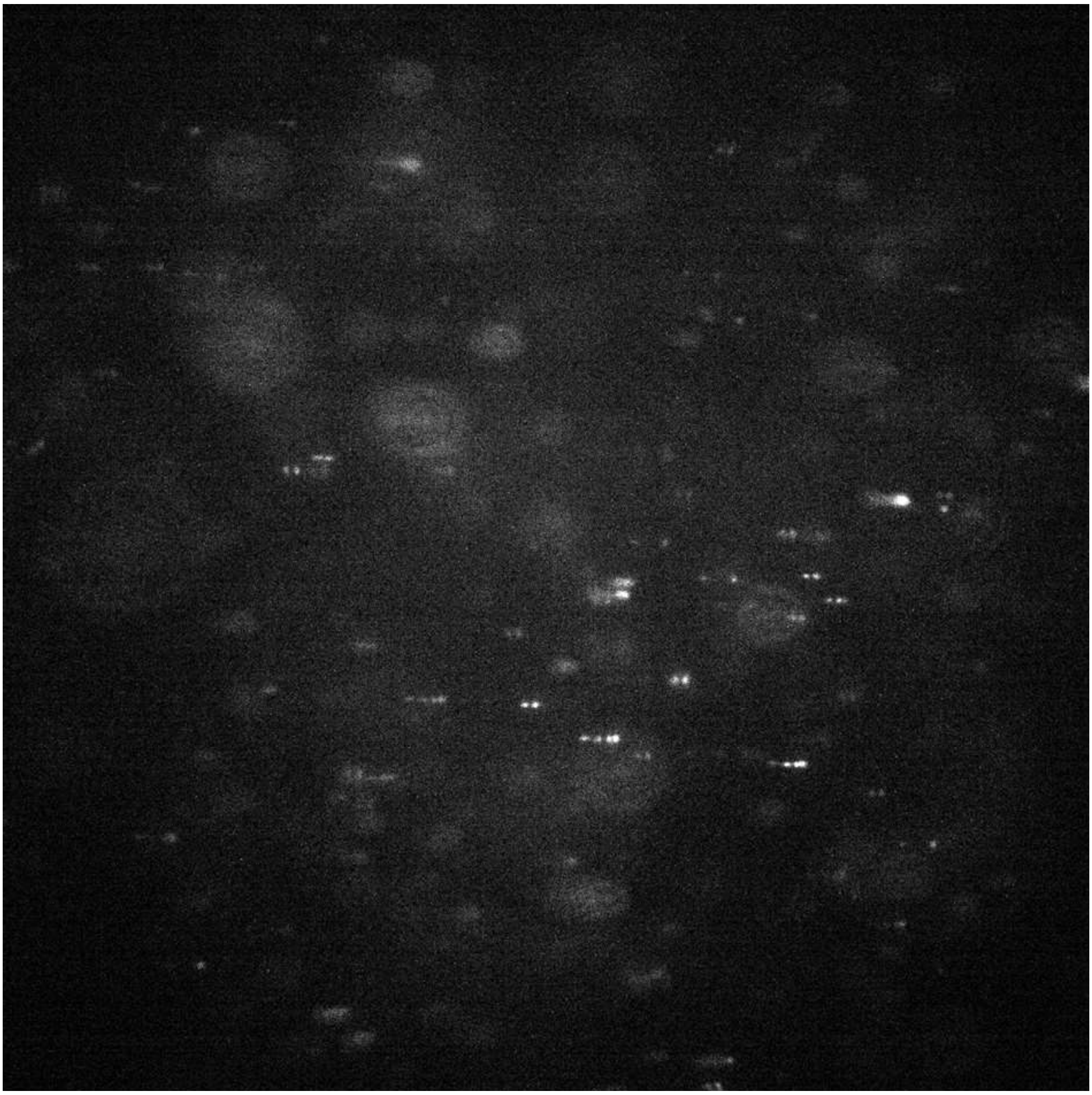

Figure S16. Full frame of Figure 3, panel n (image size 1024 px × 1024 px; 111 µm × 111 µm).

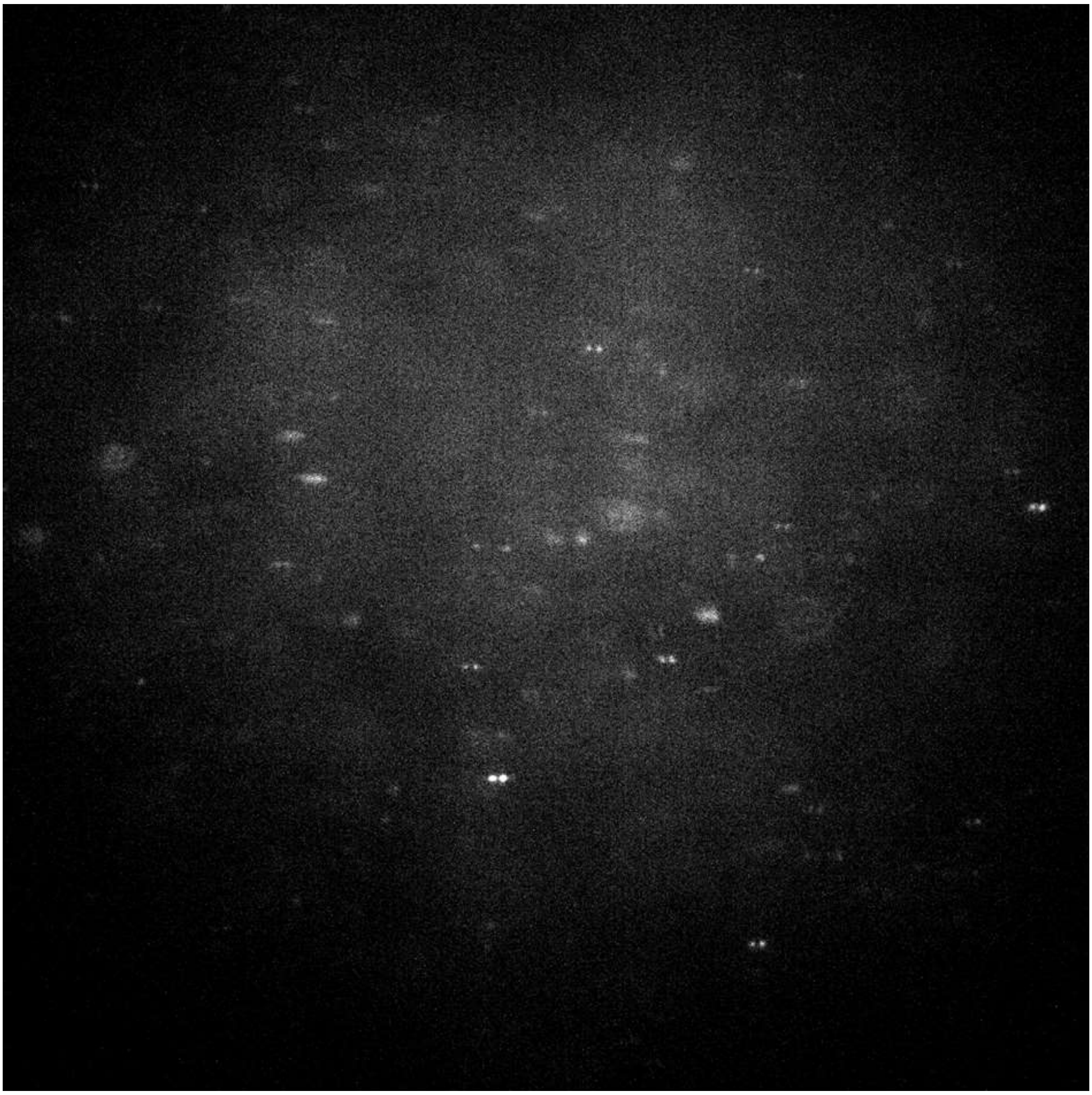

Figure S17. Full frame of Figure 3, panel o (image size 1024 px × 1024 px; 111 µm × 111 µm).

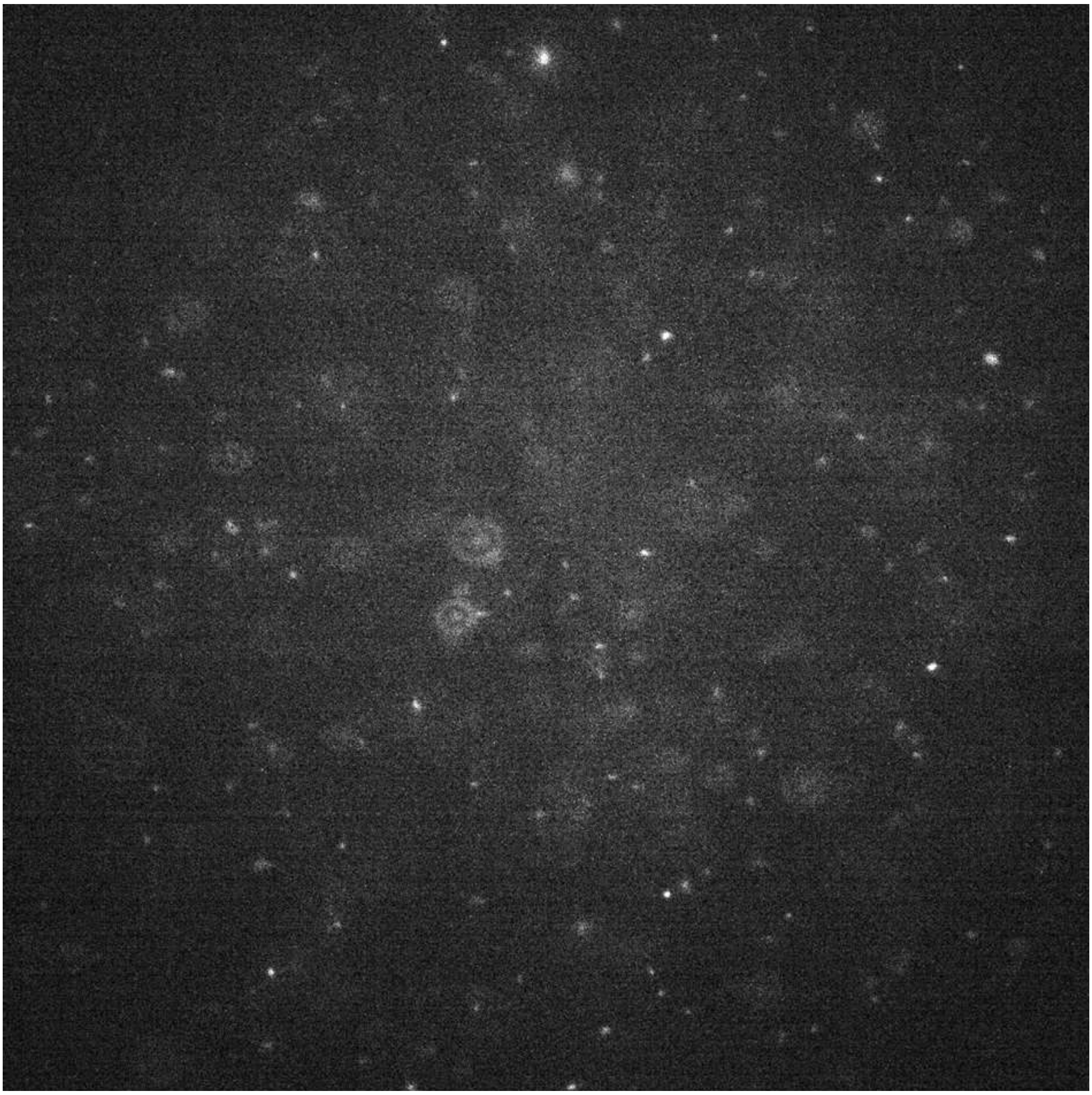

Figure S18. Full frame of Figure 3, panel p (image size 1024 px × 1024 px; 111 µm × 111 µm).

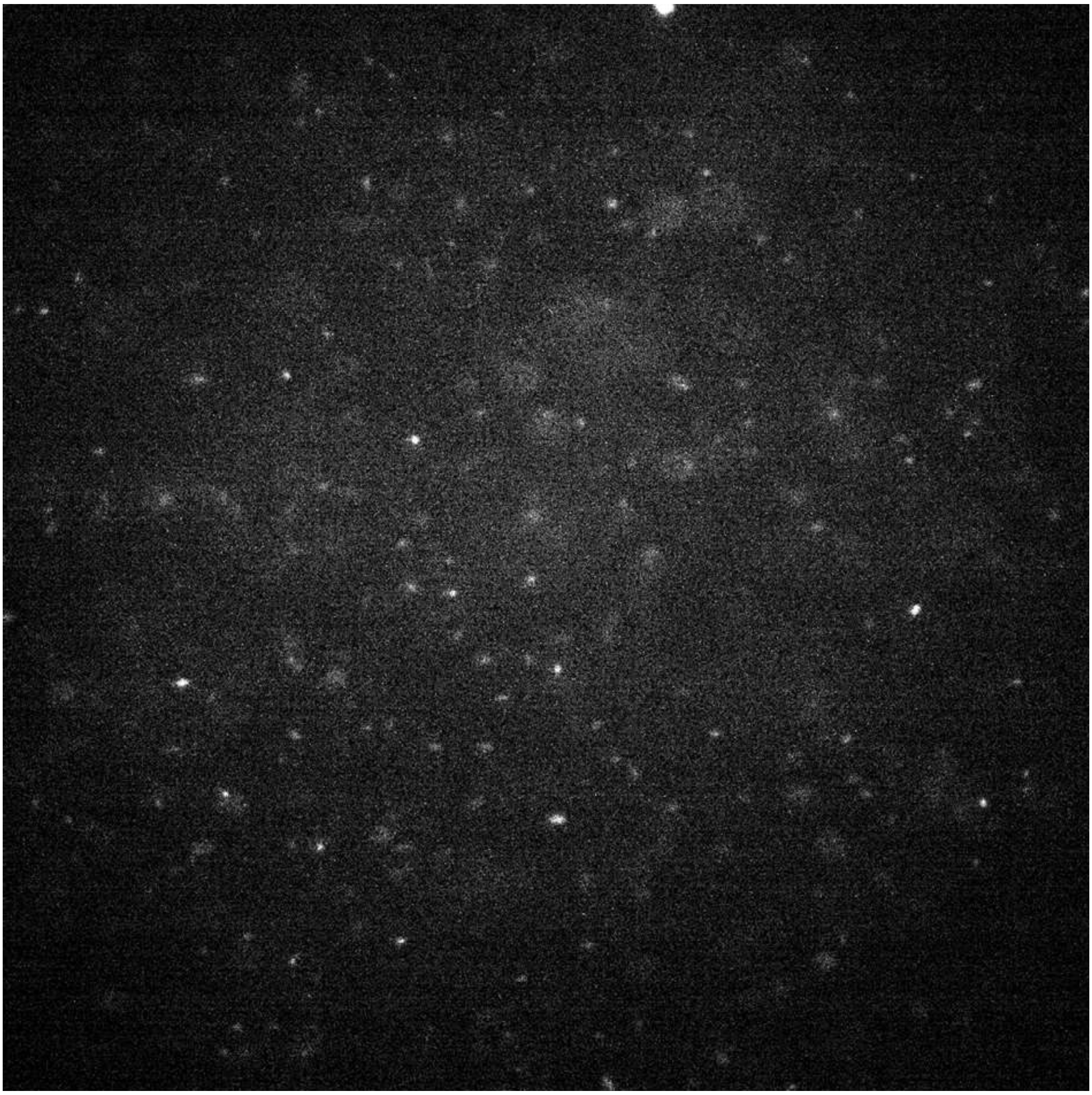

Figure S19. Full frame of Figure 3, panel q (image size 1024 px × 1024 px; 111 µm × 111 µm).

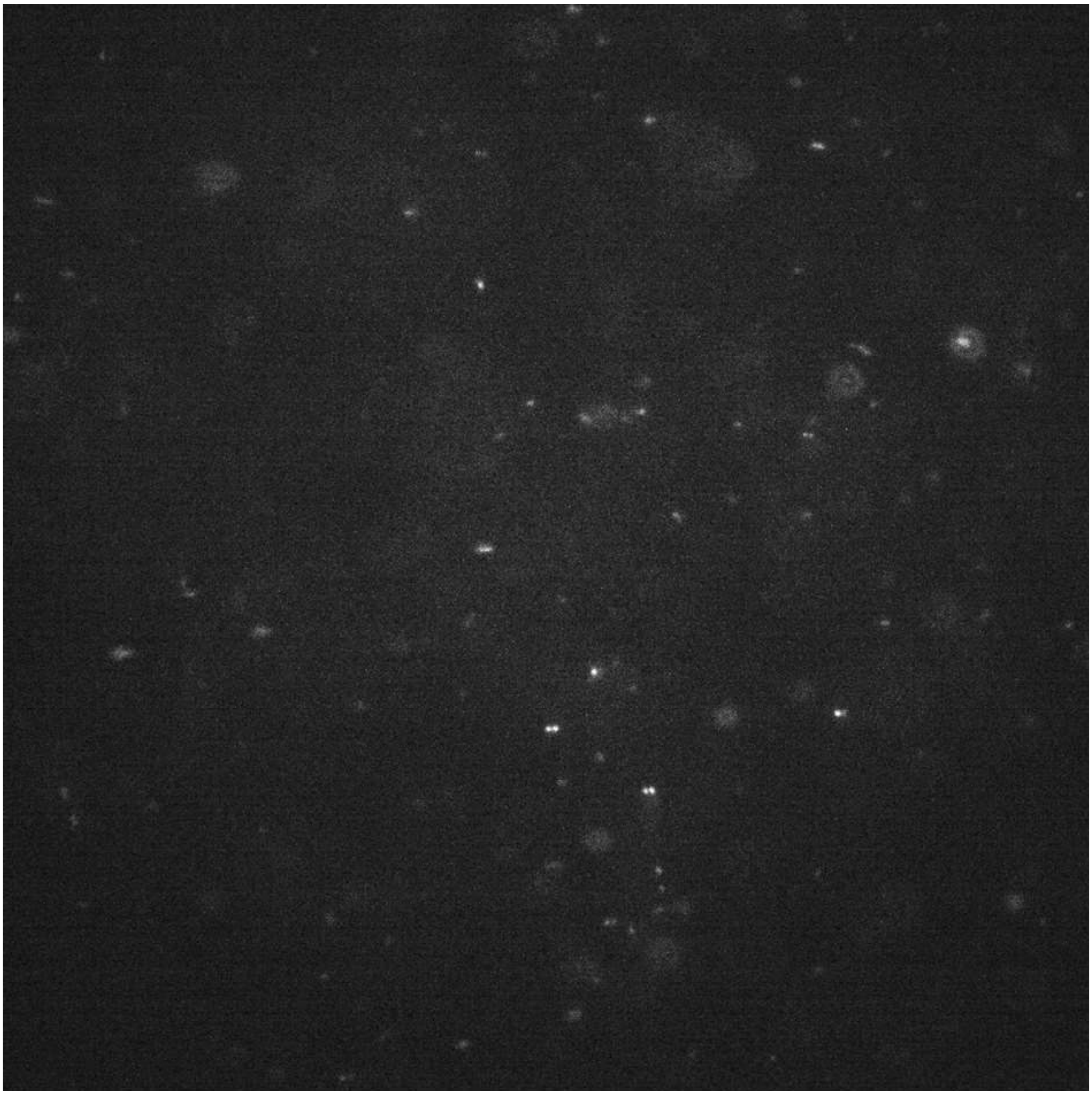

Figure S20. Full frame of Figure 3, panel r (image size 1024 px × 1024 px; 111 µm × 111 µm).

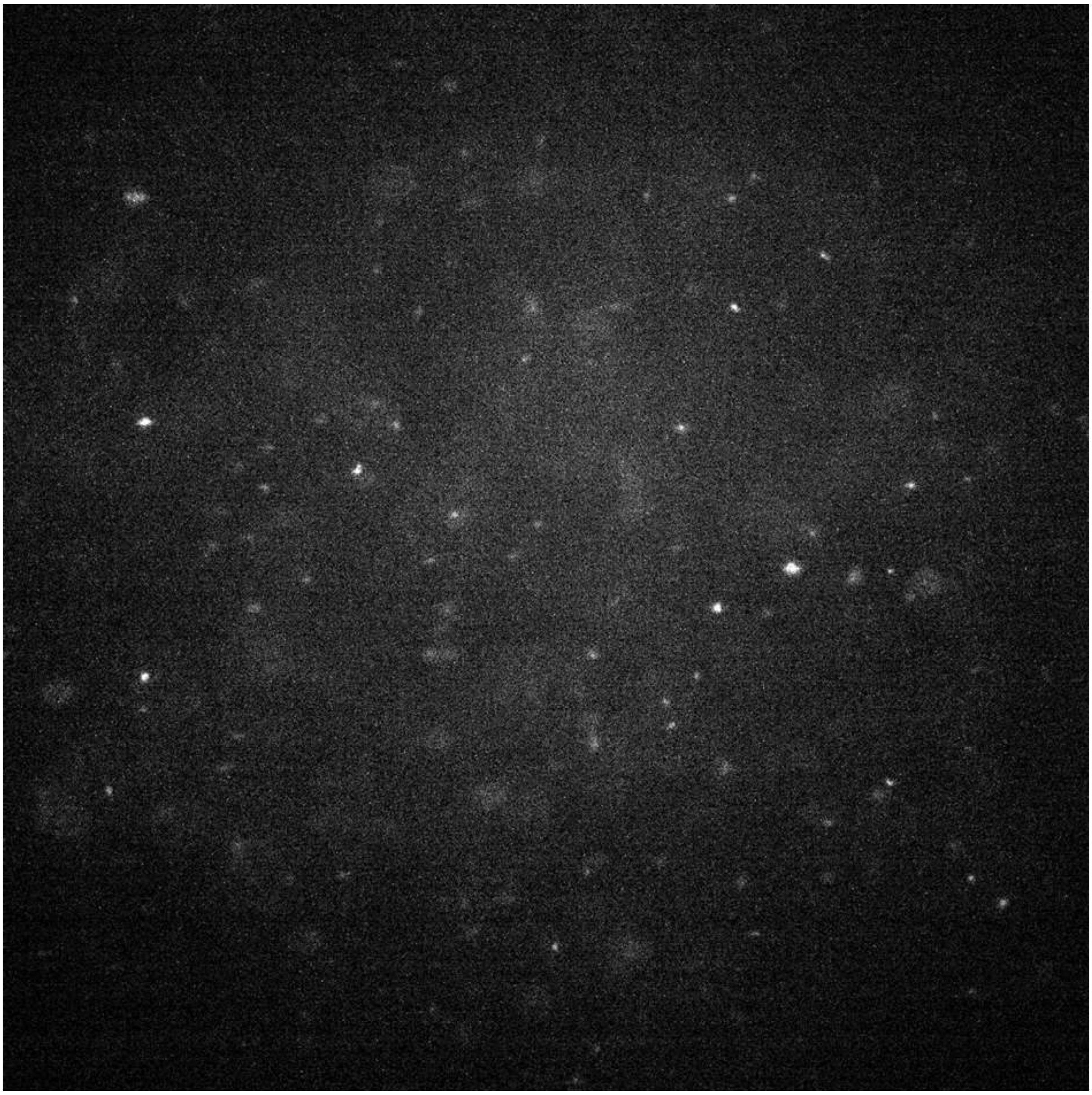

Figure S21. Full frame of Figure 3, panel s (image size 1024 px × 1024 px; 111 µm × 111 µm).

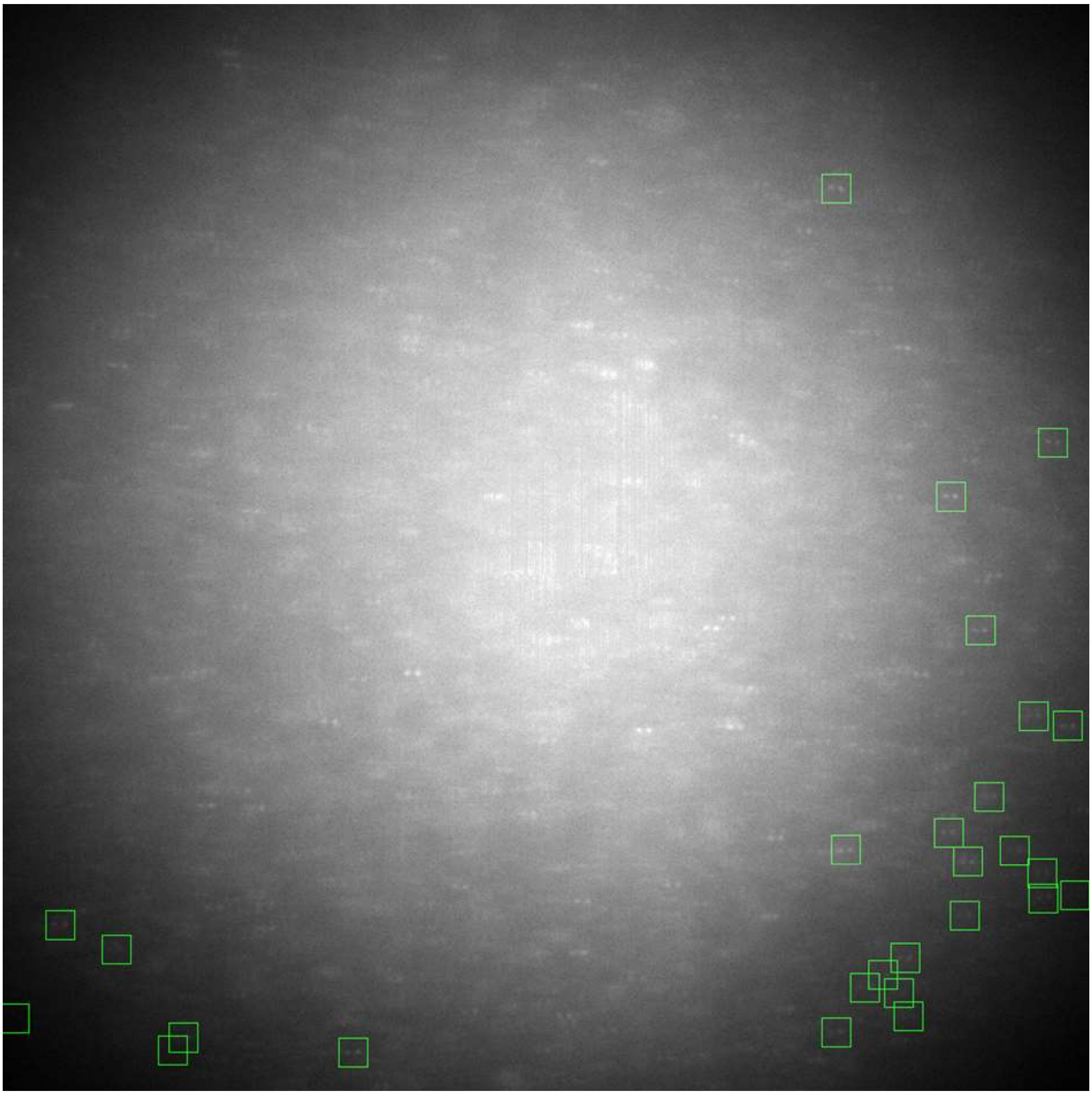

Figure S22. A representative MPS image from a set of 2 × 1000 MPS images, which were recorded for UCNP-Er-Streptavidin in acetate buffer diluted to 1 × 10^{-9} mol L^{-1}. The green rectangles mark the localization of double spots of Er^{3+} emission spectra (full spectra 430 nm – 875 nm, image size 1024 px × 1024 px; 111 µm × 111 µm). At this concentration, the localization failed because of overlapping spectra and high emission from nanoparticles, which were out of objective focal plane.

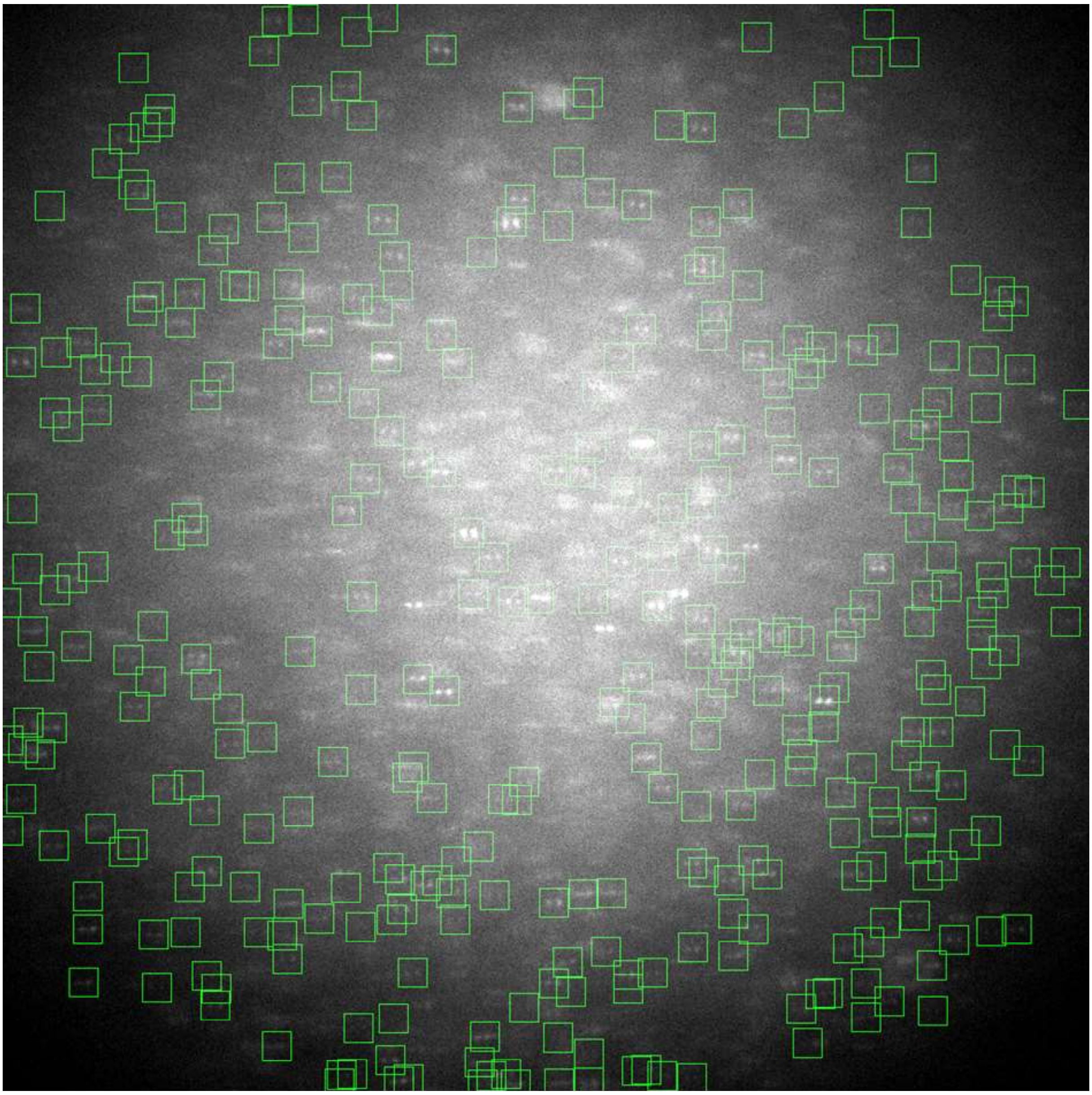

Figure S23. A representative MPS image from a set of 2 × 1000 MPS images, which were recorded for UCNP-Er-Streptavidin in acetate buffer diluted to 2 × 10^{-10} mol L^{-1}. The green rectangles mark the localization of double spots of Er^{3+} emission spectra (full spectra 430 nm – 875 nm, image size 1024 px × 1024 px; 111 µm × 111 µm). At this concentration, the localization failed because of overlapping spectra and high emission from nanoparticles, which were out of objective focal plane.

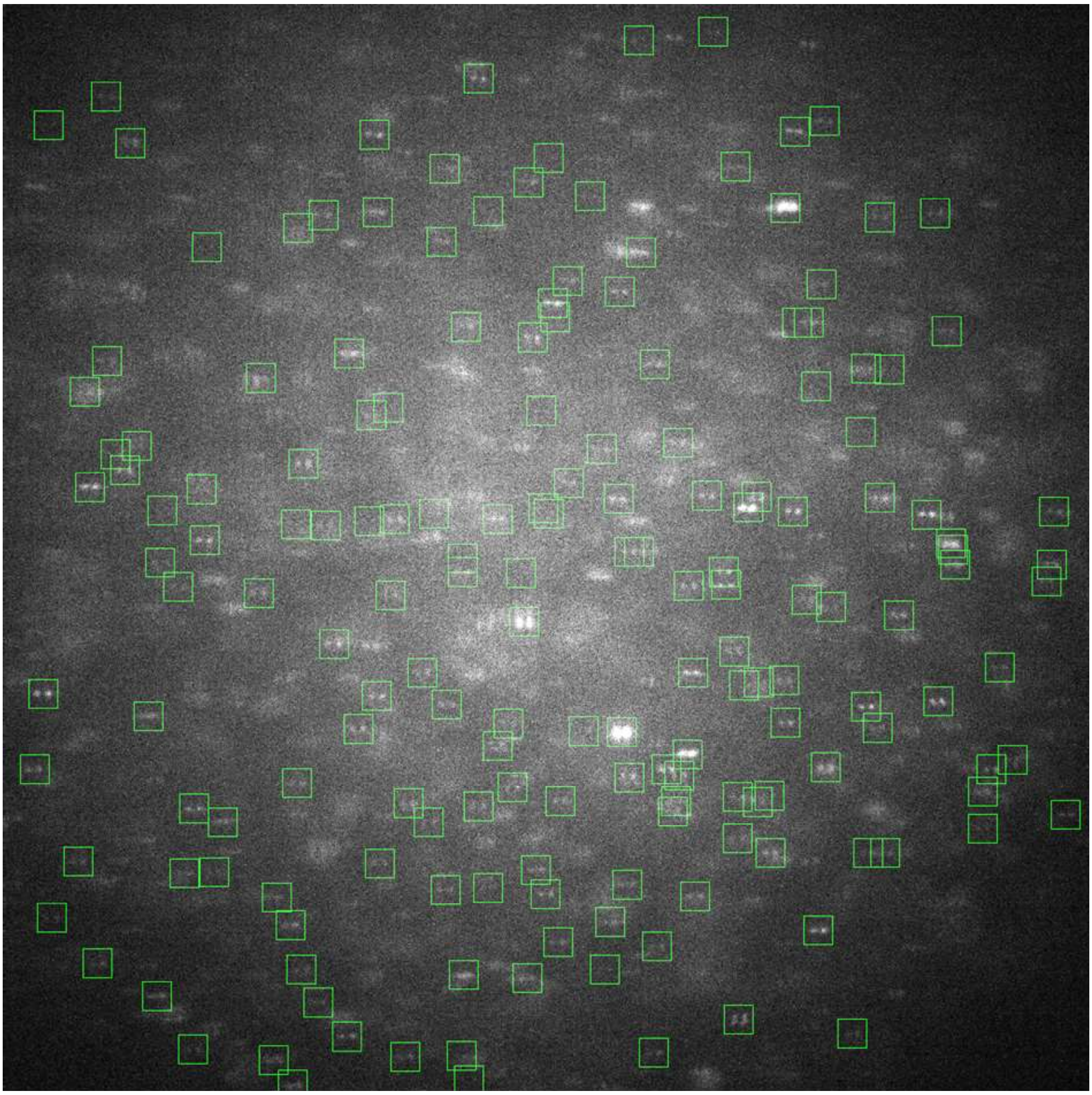

Figure S24. A representative MPS image from a set of 2 × 1000 MPS images, which were recorded for UCNP-Er-Streptavidin in acetate buffer diluted to 4 × 10^{-11} mol L^{-1}. The green rectangles mark the localization of double spots of Er^{3+} emission spectra (full spectra 430 nm – 875 nm, image size 1024 px × 1024 px; 111 µm × 111 µm).

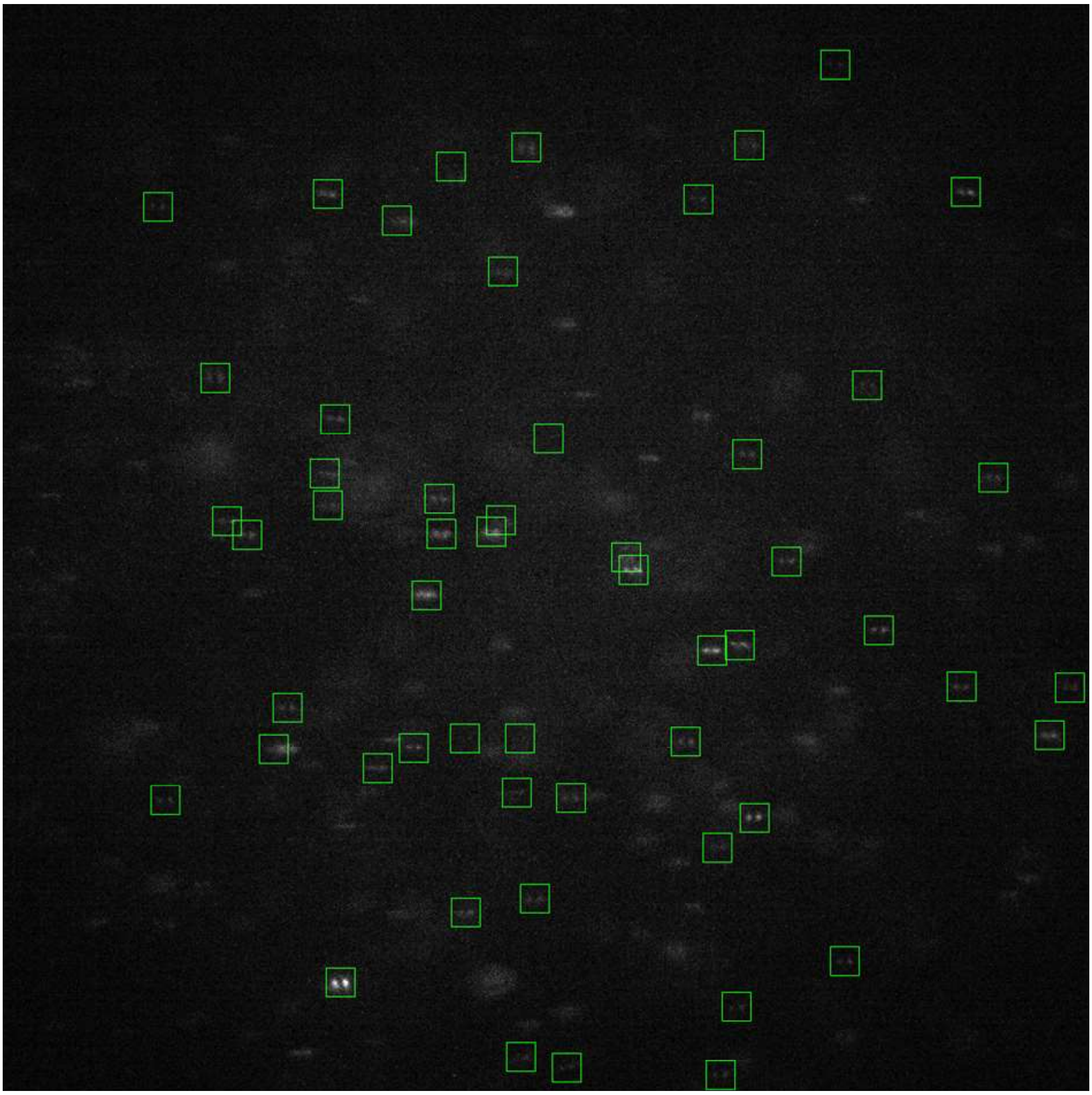

Figure S25. A representative MPS image from a set of 2 × 1000 MPS images, which were recorded for UCNP-Er-Streptavidin in acetate buffer diluted to 8×10^{-12} mol L^{-1}. The green rectangles mark the localization of double spots of Er^{3+} emission spectra (full spectra 430 nm – 875 nm, image size 1024 px × 1024 px; 111 µm × 111 µm).

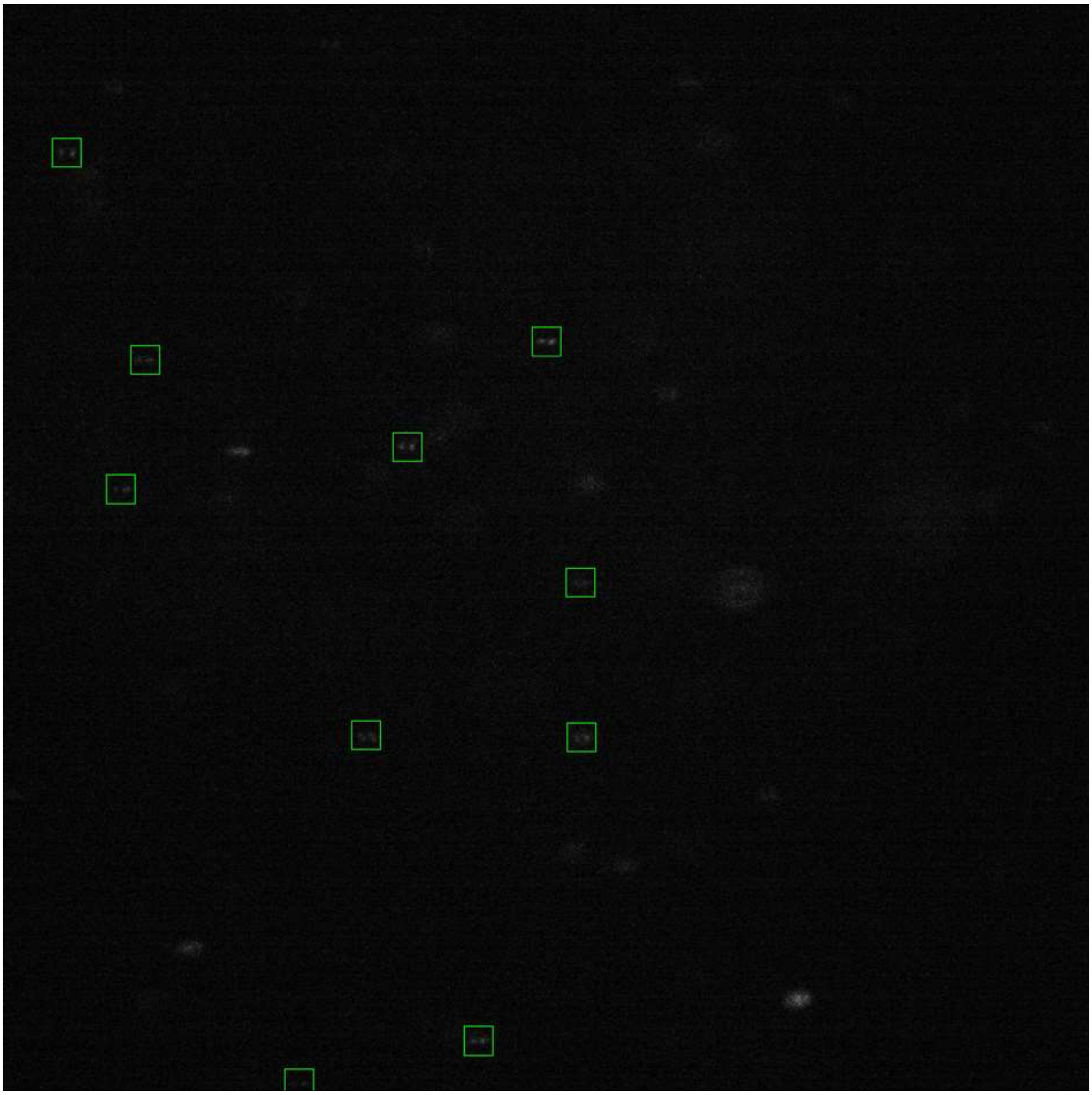

Figure S26. A representative MPS image from a set of 2 × 1000 MPS images, which were recorded for UCNP-Er-Streptavidin in acetate buffer diluted to 1.6 × 10^{-12} mol L^{-1}. The green rectangles mark the localization of double spots of Er^{3+} emission spectra (full spectra 430 nm – 875 nm, image size 1024 px × 1024 px; 111 µm × 111 µm).

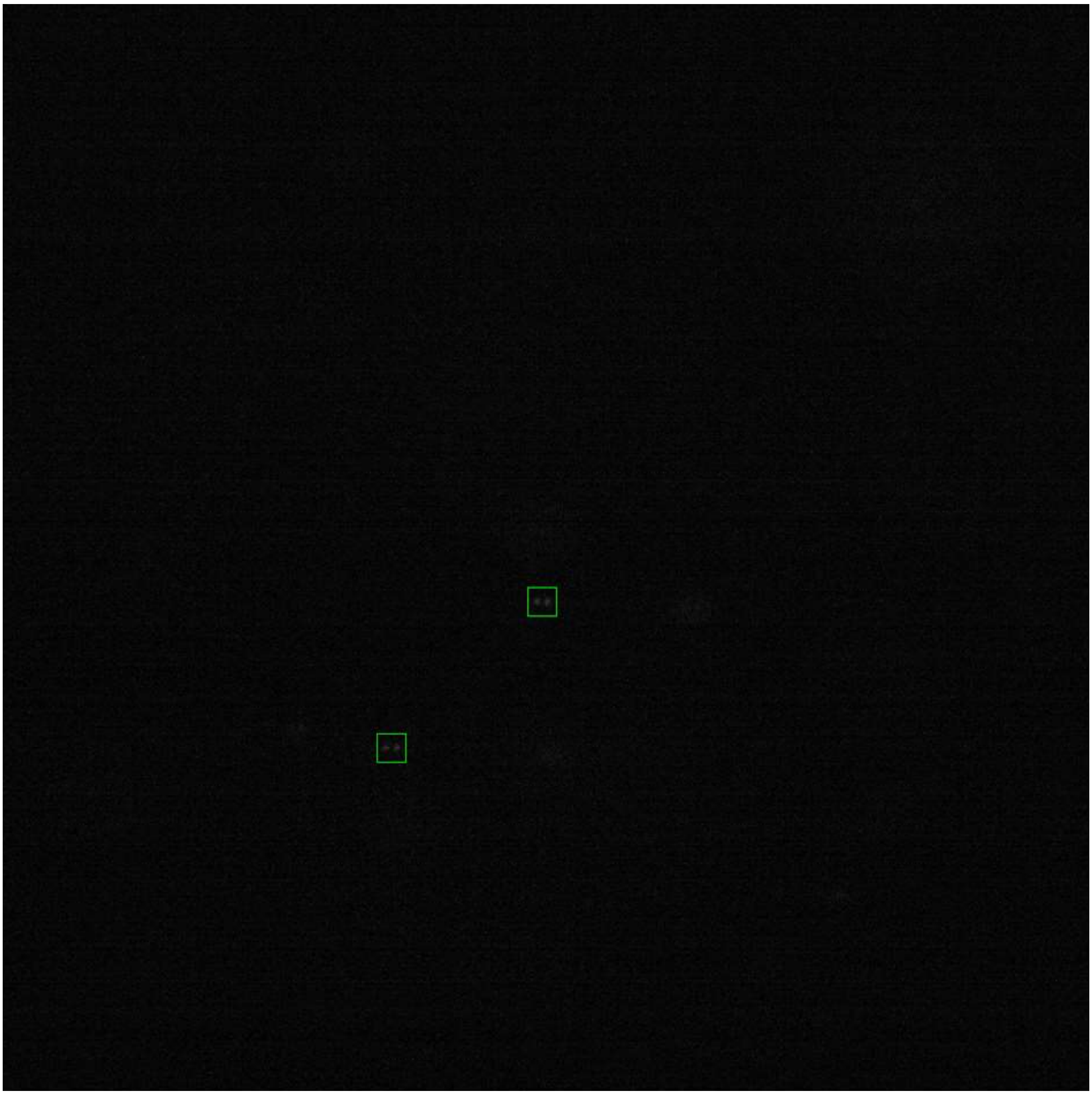

Figure S27. A representative MPS image from a set of 2 × 1000 MPS images, which were recorded for UCNP-Er-Streptavidin in acetate buffer diluted to 3.2×10^{-13} mol L^{-1}. The green rectangles mark the localization of double spots of Er^{3+} emission spectra (full spectra 430 nm – 875 nm, image size 1024 px × 1024 px; 111 µm × 111 µm).

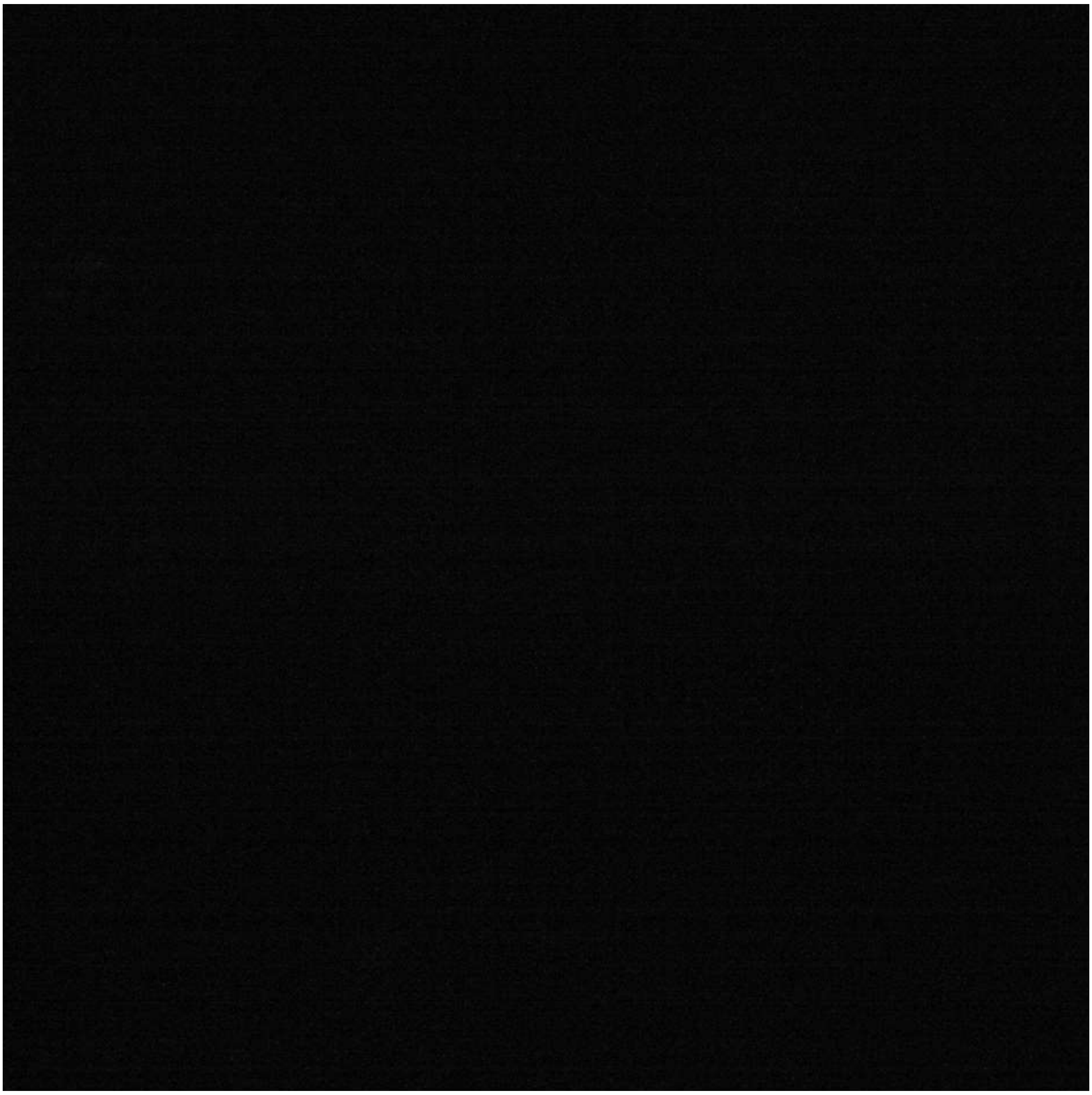

Figure S28. A representative MPS image from a set of 2 × 1000 MPS images, which were recorded for UCNP-Er-Streptavidin in acetate buffer diluted to 6.4 × 10^{-14} mol L^{-1}. No double spots of Er^{3+} emission spectra were localized (full spectra 430 nm – 875 nm, image size 1024 px × 1024 px; 111 µm × 111 µm).

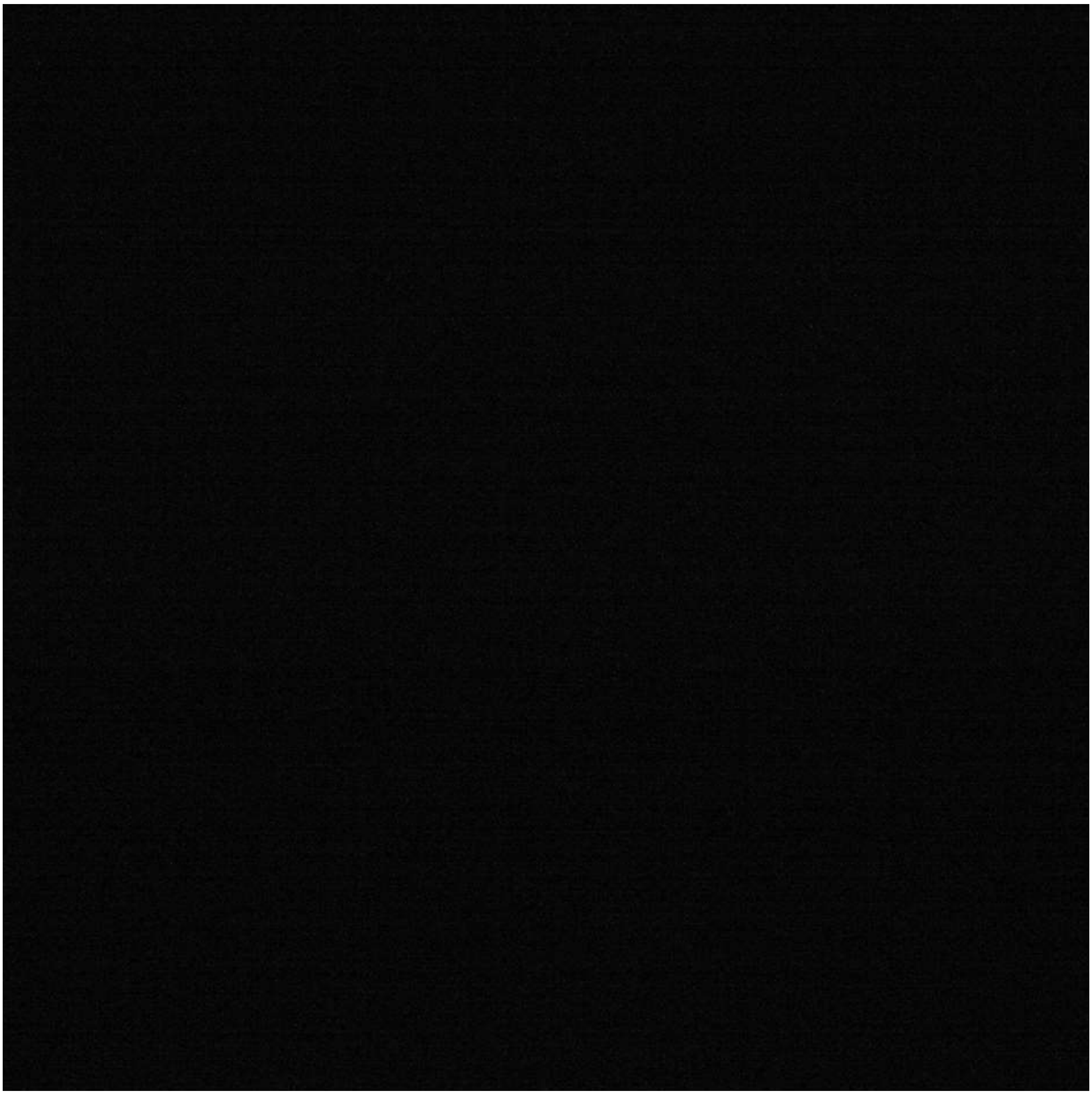

Figure S29. A representative MPS image from a set of 2 × 1000 MPS images, which were recorded for UCNP-Er-Streptavidin in acetate buffer diluted to 1.3 × 10^{-14} mol L^{-1}. No double spots of Er^{3+} emission spectra were localized (full spectra 430 nm – 875 nm, image size 1024 px × 1024 px; 111 µm × 111 µm).

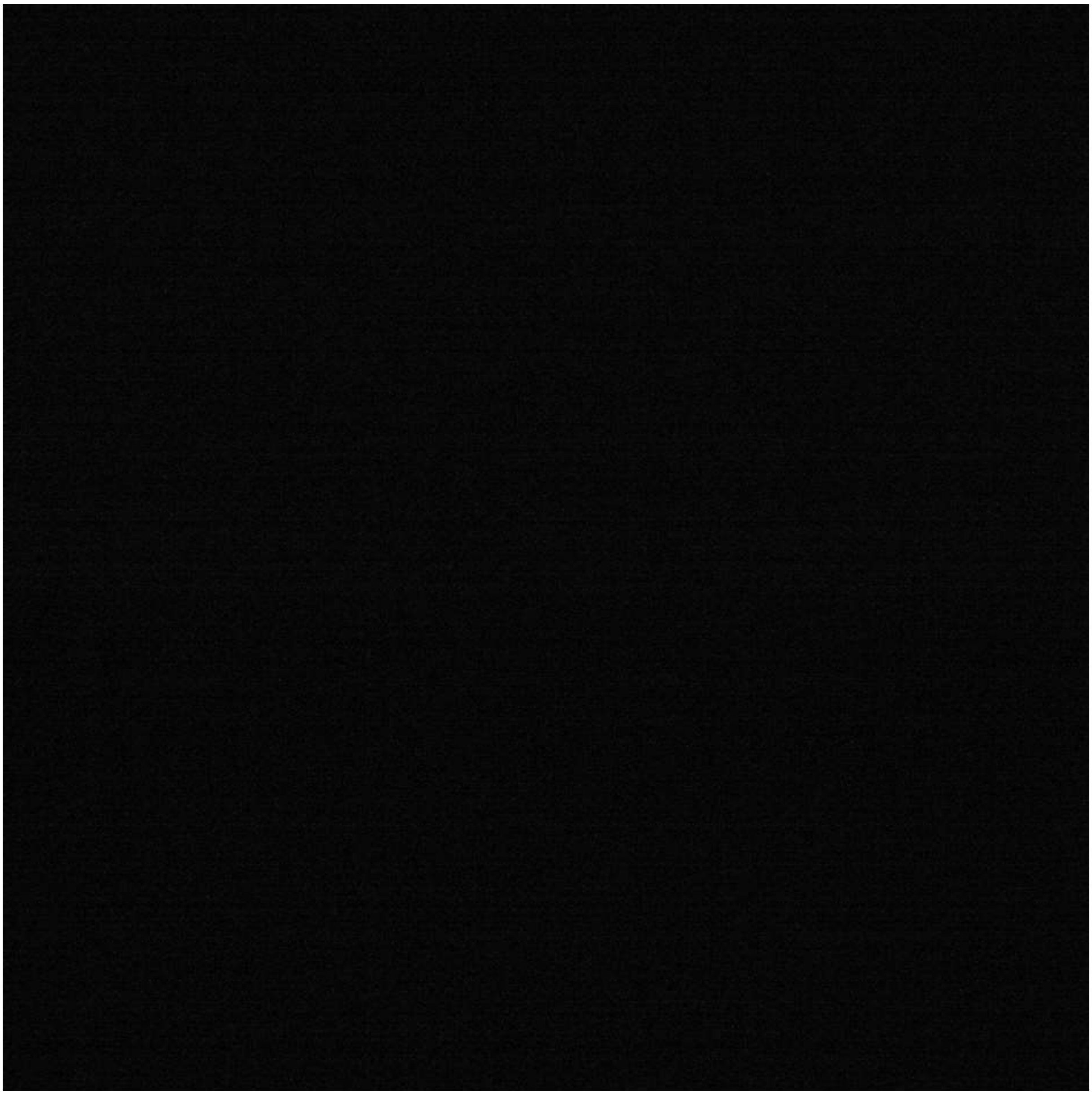

Figure S30. A representative MPS image from a set of 2 × 1000 MPS images, which were recorded for UCNP-Er-Streptavidin in acetate buffer diluted to 2.6×10^{-15} mol L^{-1}. No double spots of Er^{3+} emission spectra were localized (full spectra 430 nm – 875 nm, image size 1024 px × 1024 px; 111 µm × 111 µm).

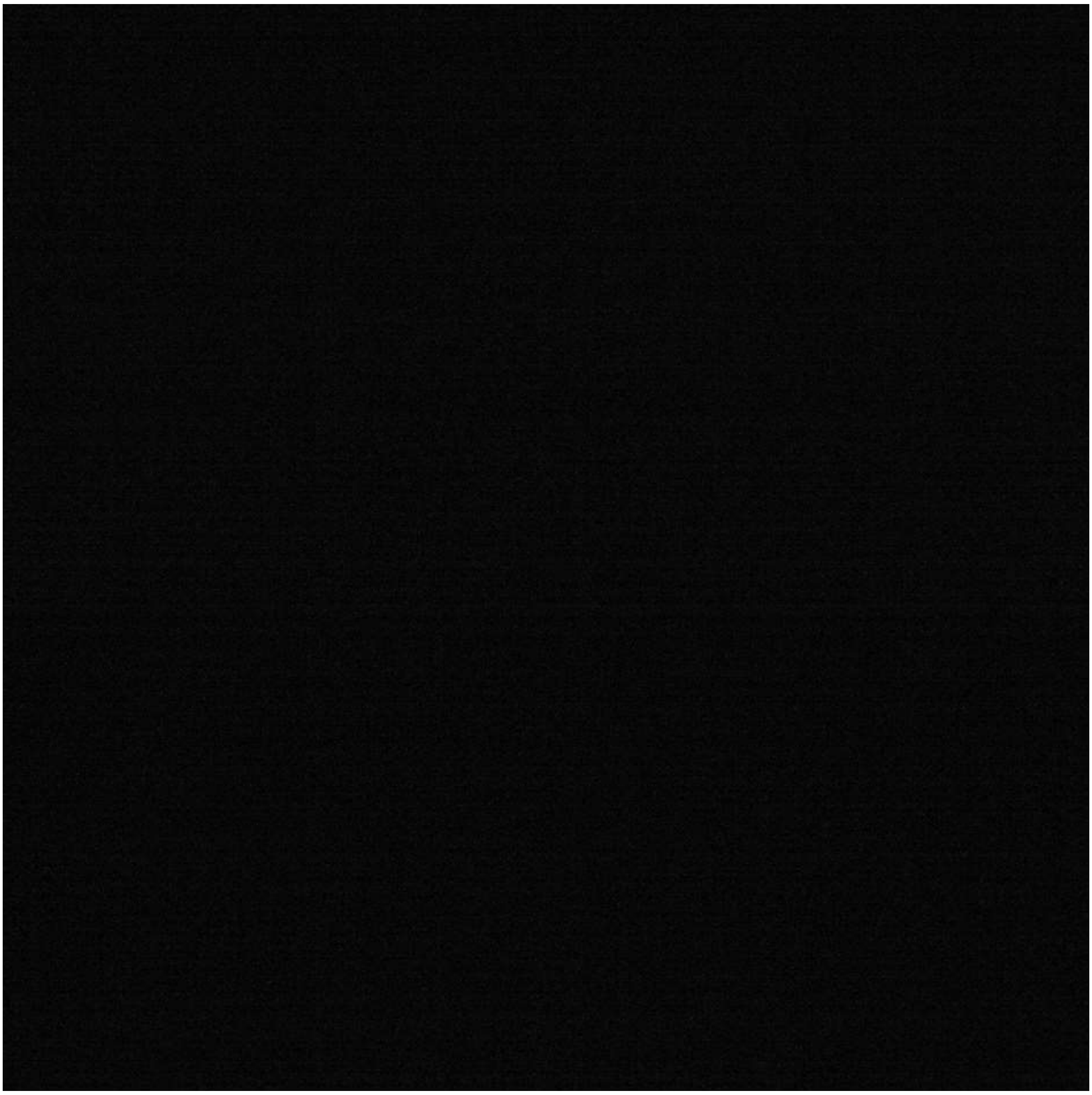

Figure S31. A representative MPS image from a set of 2 × 1000 MPS images, which were recorded for a blank experiment (acetate buffer). No double spots of Er^{3+} emission spectra were localized (full spectra 430 nm – 875 nm, image size 1024 px × 1024 px; 111 μm × 111 μm).

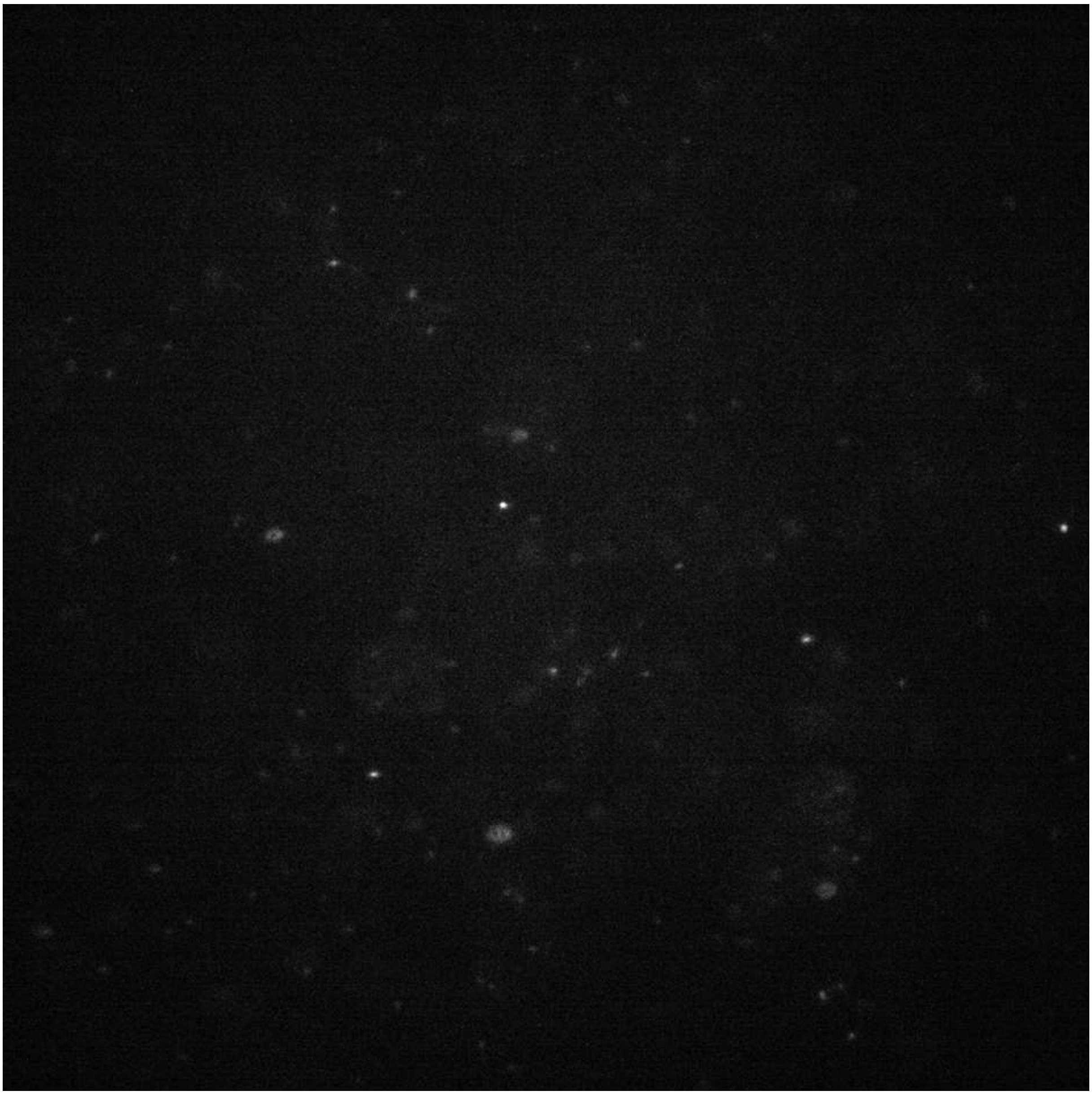

Figure S32. A representative MPS image from a set of 2 × 1000 MPS images, which were recorded for 10^{-4} mol L^{-1} biotin concentration. No double spots were detected (image size 1024 px × 1024 px; 111 µm × 111 µm).

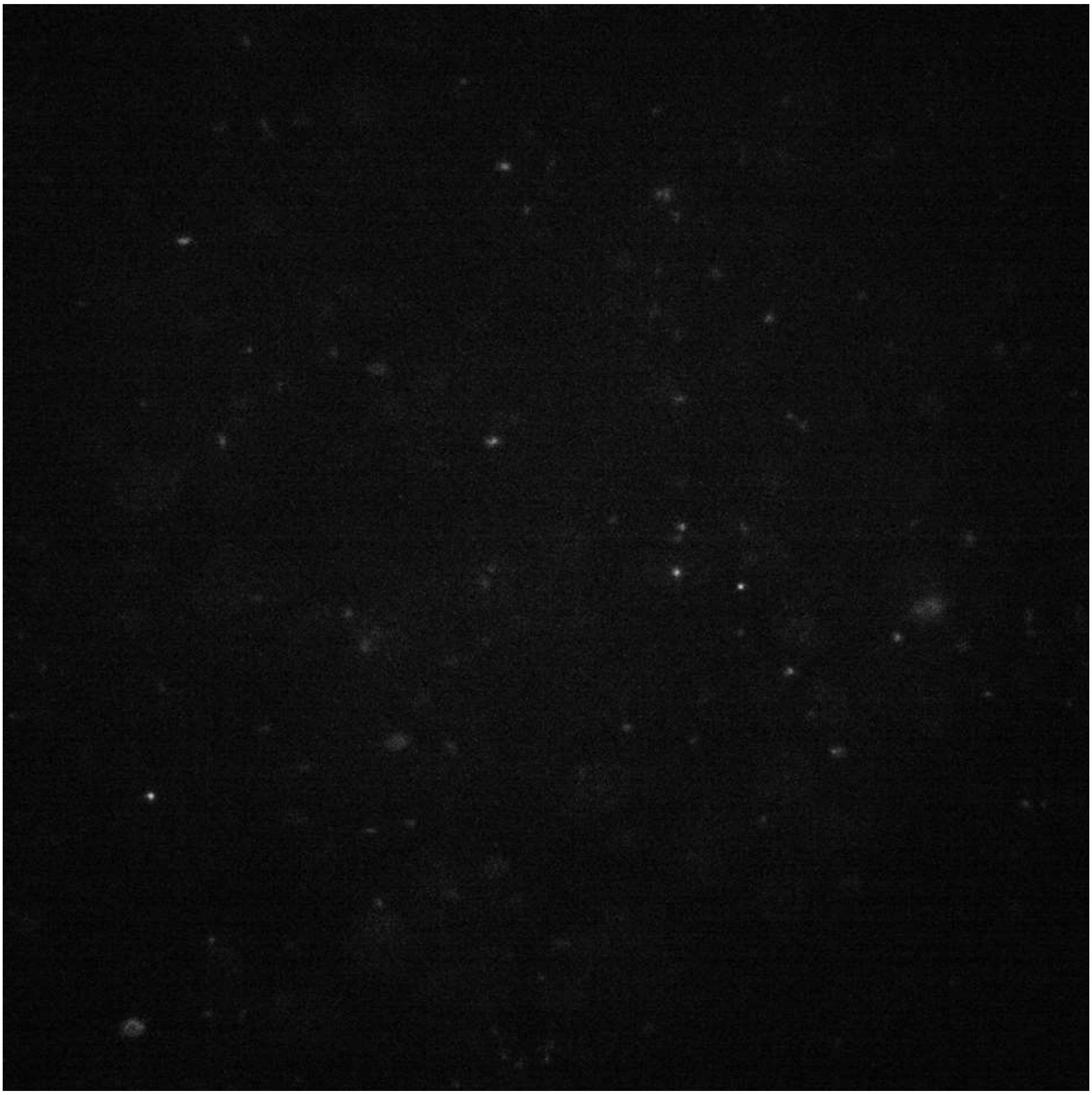

Figure S33. A representative MPS image from a set of 2 × 1000 MPS images, which were recorded for 10^{-5} mol L^{-1} biotin concentration. No double spots were detected (image size 1024 px × 1024 px; 111 μm × 111 μm).

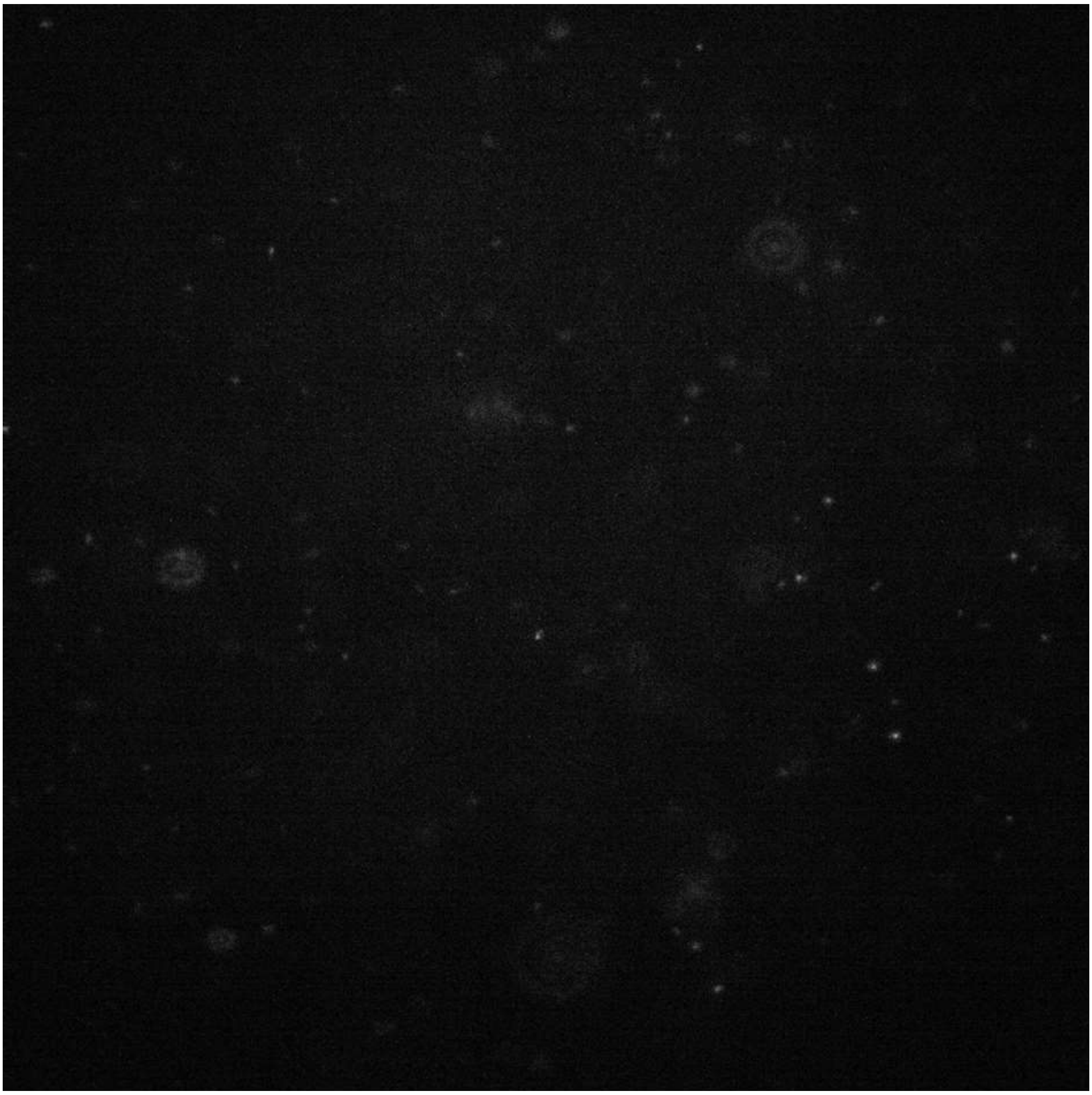

Figure S34. A representative MPS image from a set of 2 × 1000 MPS images, which were recorded for 10^{-6} mol L^{-1} biotin concentration. No double spots were detected (image size 1024 px × 1024 px; 111 µm × 111 µm).

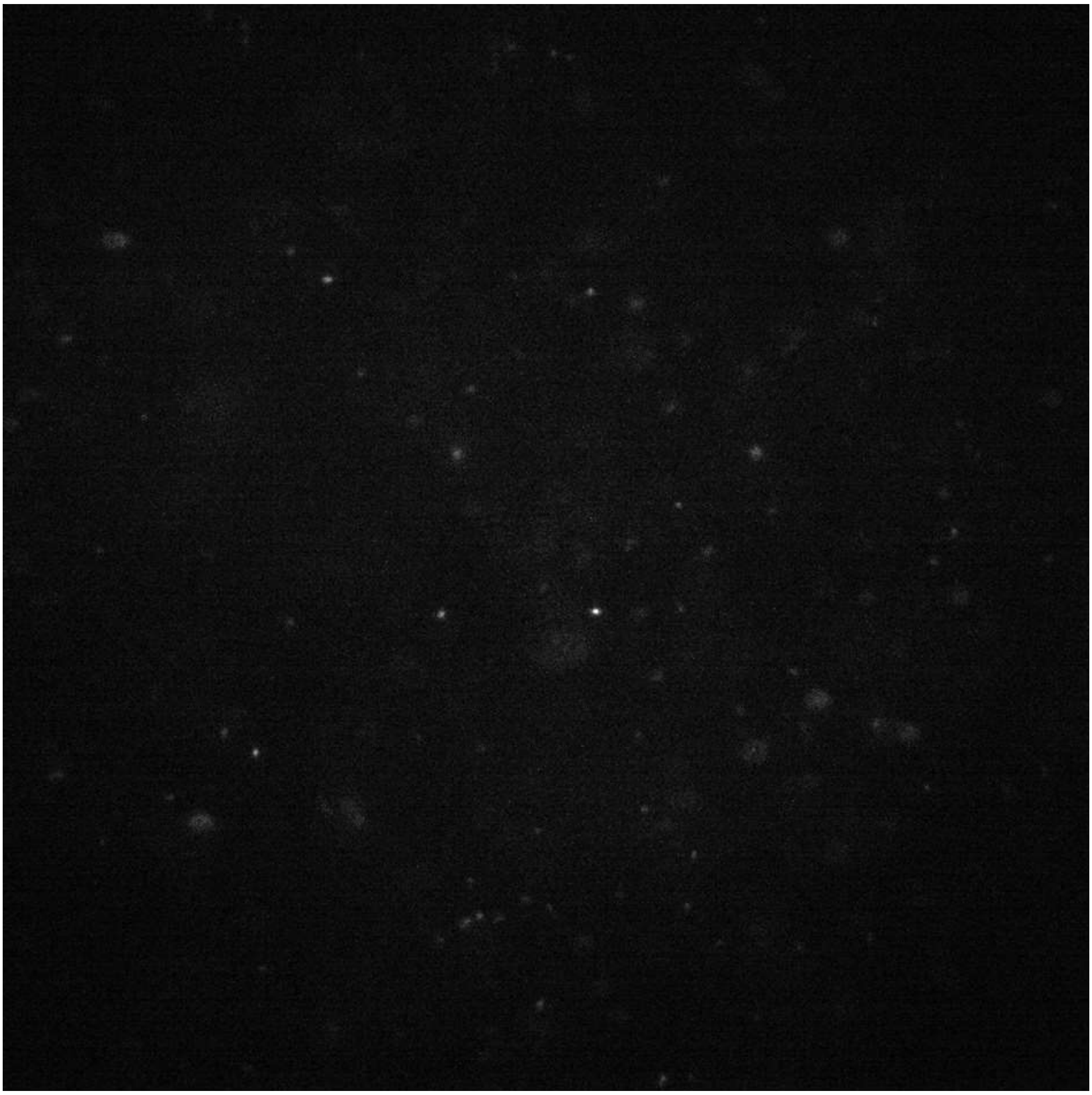

Figure S35. A representative MPS image from a set of 2 × 1000 MPS images, which were recorded for 10^{-7} mol L^{-1} biotin concentration. No double spots were detected (image size 1024 px × 1024 px; 111 μm × 111 μm).

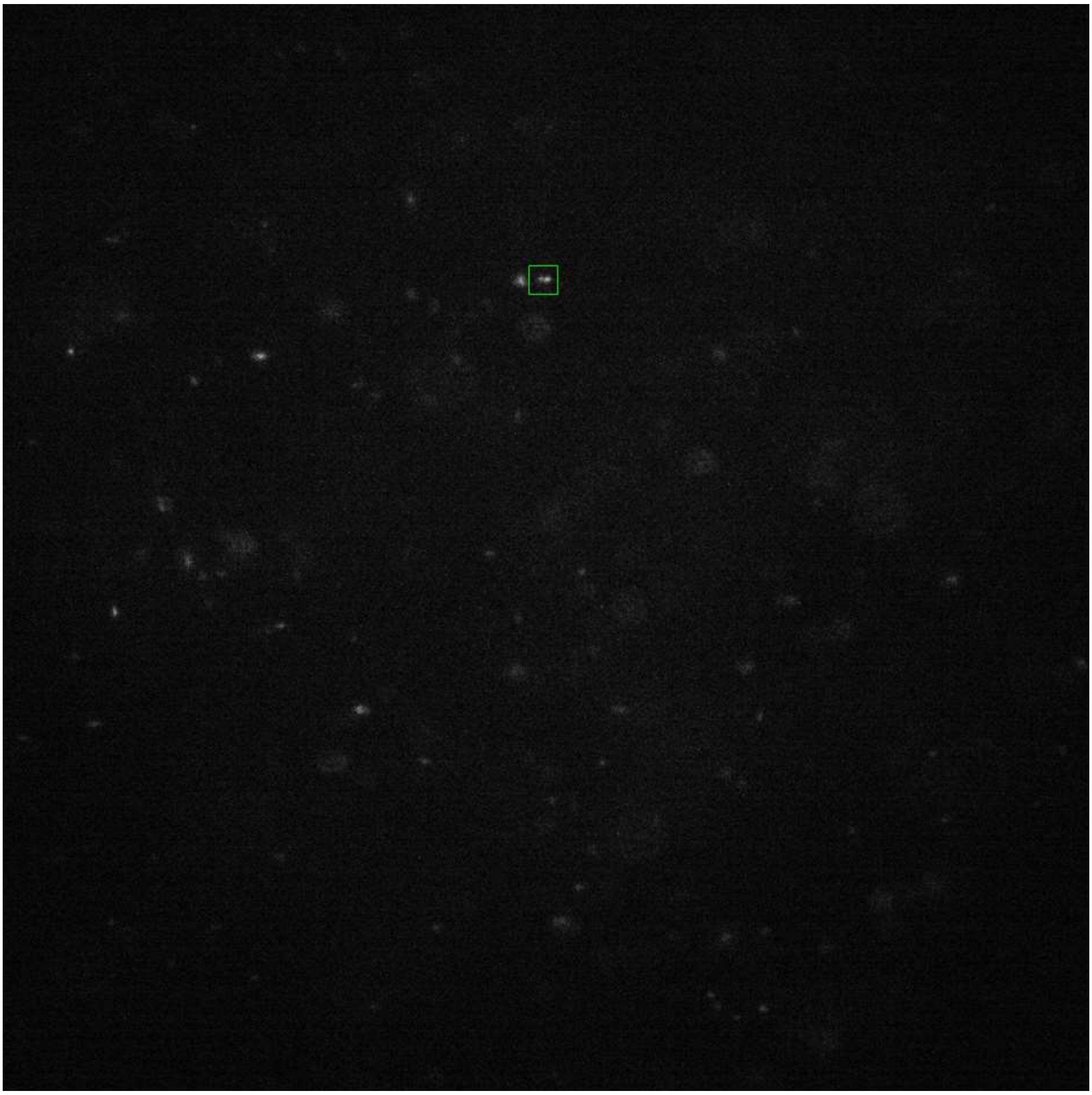

Figure S36. A representative MPS image from a set of 2 × 1000 MPS images, which were recorded for 10^{-8} mol L^{-1} biotin concentration. The green rectangle marks the localization of the double spot of Er^{3+} 660 nm and Tm^{3+} 802 nm emission peaks (spectra 600 nm – 875 nm, image size 1024 px × 1024 px; 111 μm × 111 μm).

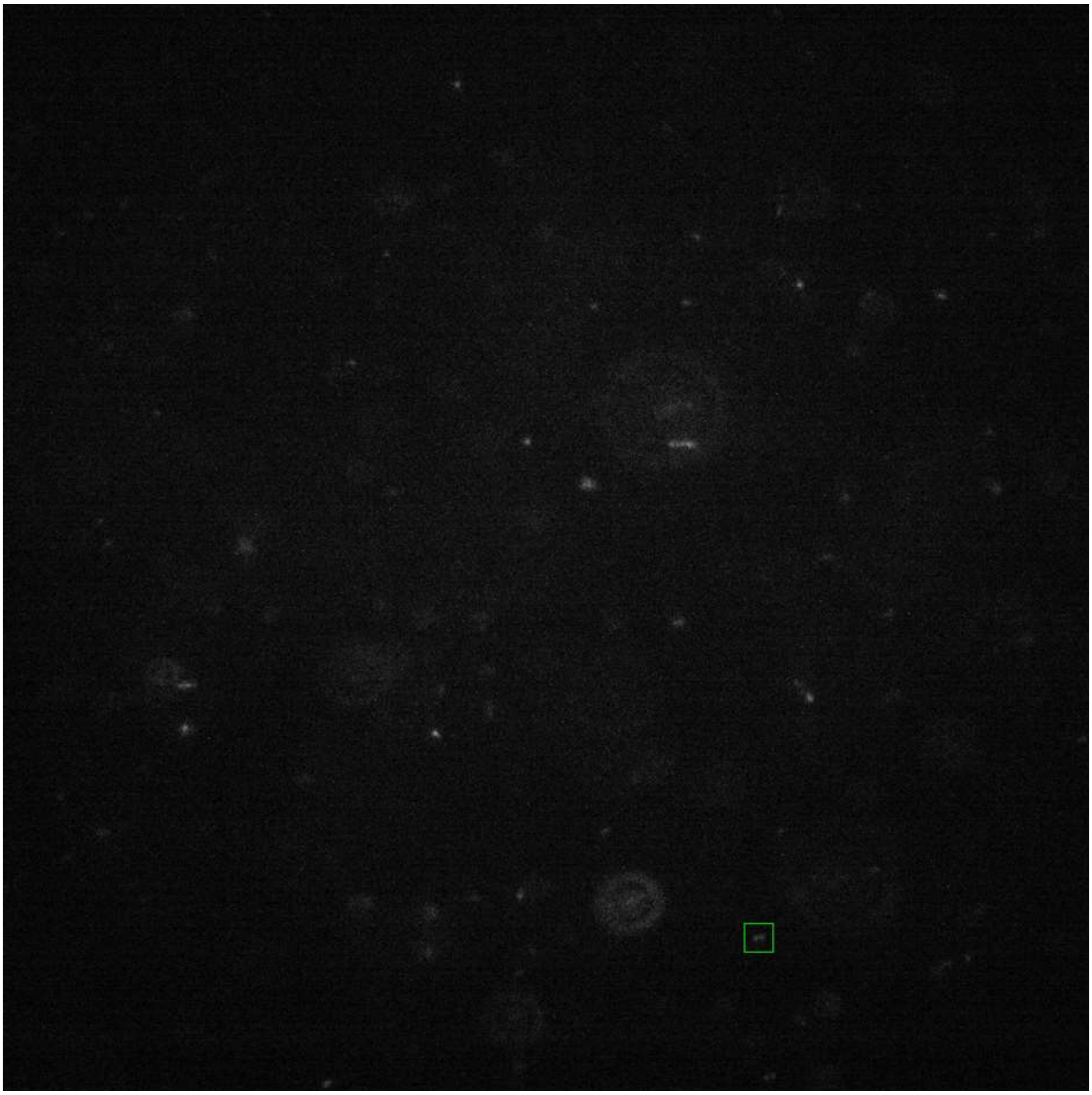

Figure S37. A representative MPS image from a set of 2 × 1000 MPS images, which were recorded for 10^{-9} mol L^{-1} biotin concentration. The green rectangle marks the localization of the double spot of Er^{3+} 660 nm and Tm^{3+} 802 nm emission peaks (spectra 600 nm – 875 nm, image size 1024 px × 1024 px; 111 µm × 111 µm).

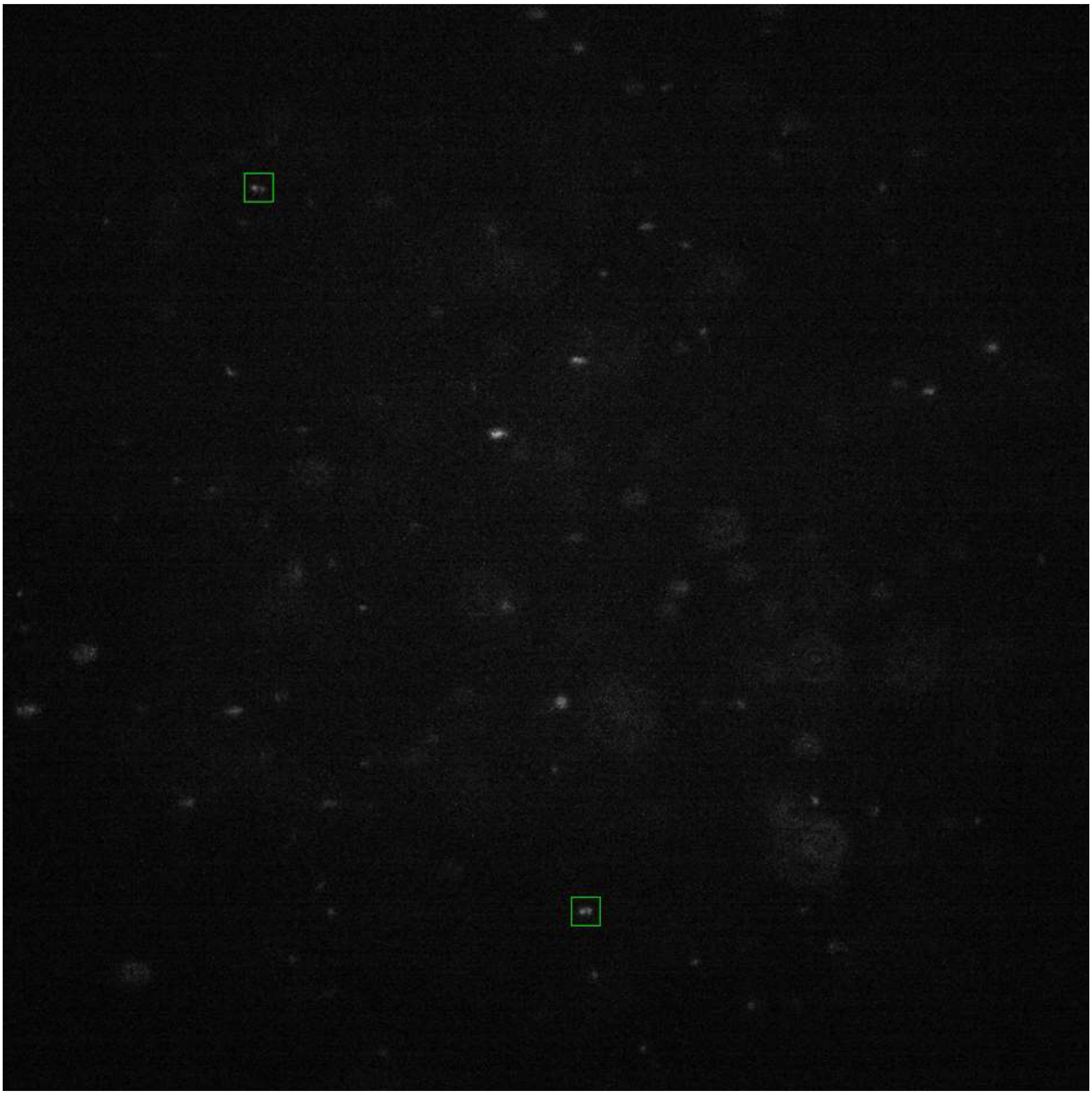

Figure S38. A representative MPS image from a set of 2 × 1000 MPS images, which were recorded for 10^{-10} mol L^{-1} biotin concentration. The green rectangles mark the localization of double spots of Er^{3+} 660 nm and Tm^{3+} 802 nm emission peaks (spectra 600 nm – 875 nm, image size 1024 px × 1024 px; 111 µm × 111 µm).

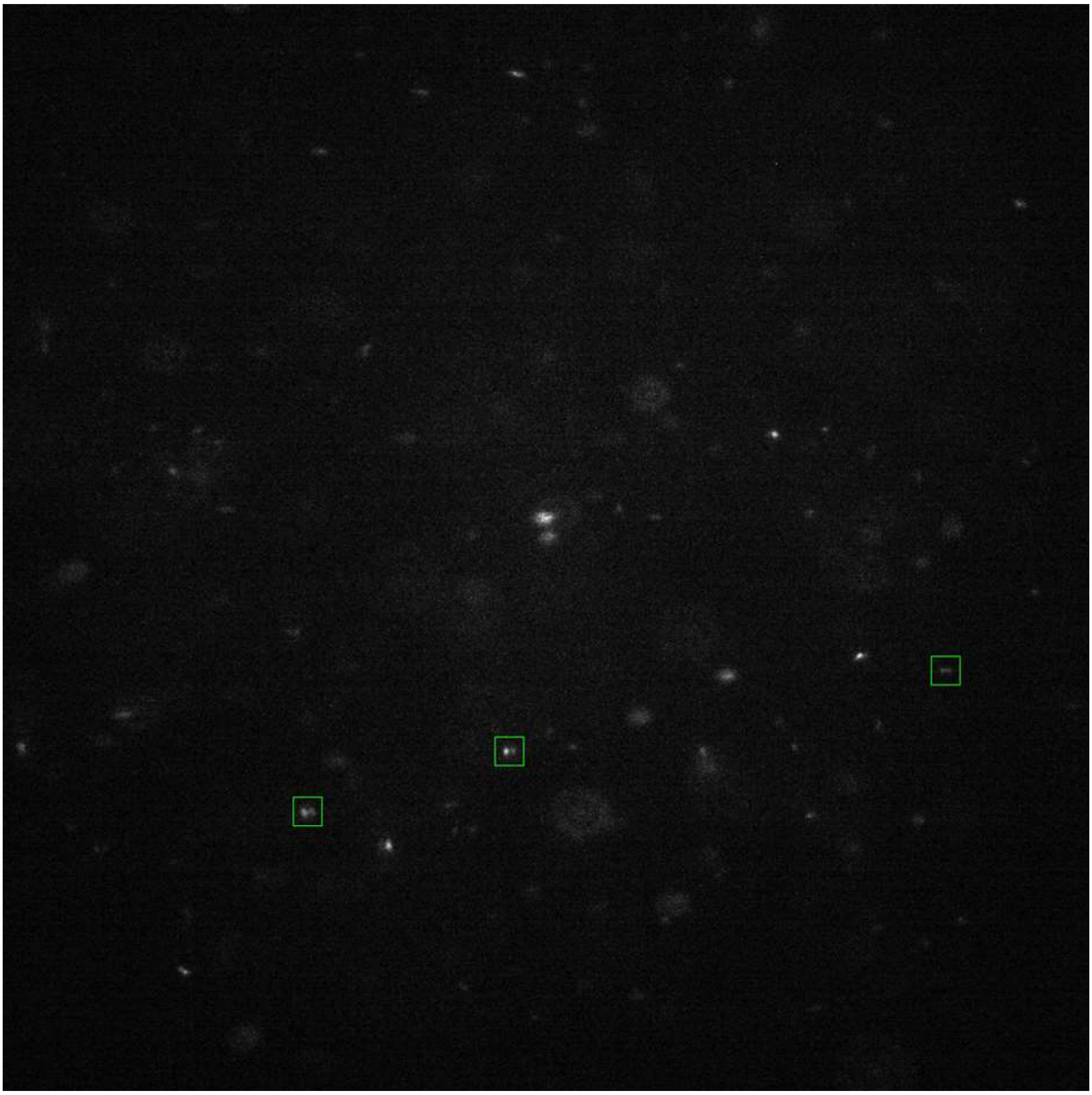

Figure S39. A representative MPS image from a set of 2 × 1000 MPS images, which were recorded for a blank experiment (no biotin). The green rectangles mark the localization of double spots of Er^{3+} 660 nm and Tm^{3+} 802 nm emission peaks (spectra 600 nm – 875 nm, image size 1024 px × 1024 px; 111 µm × 111 µm).

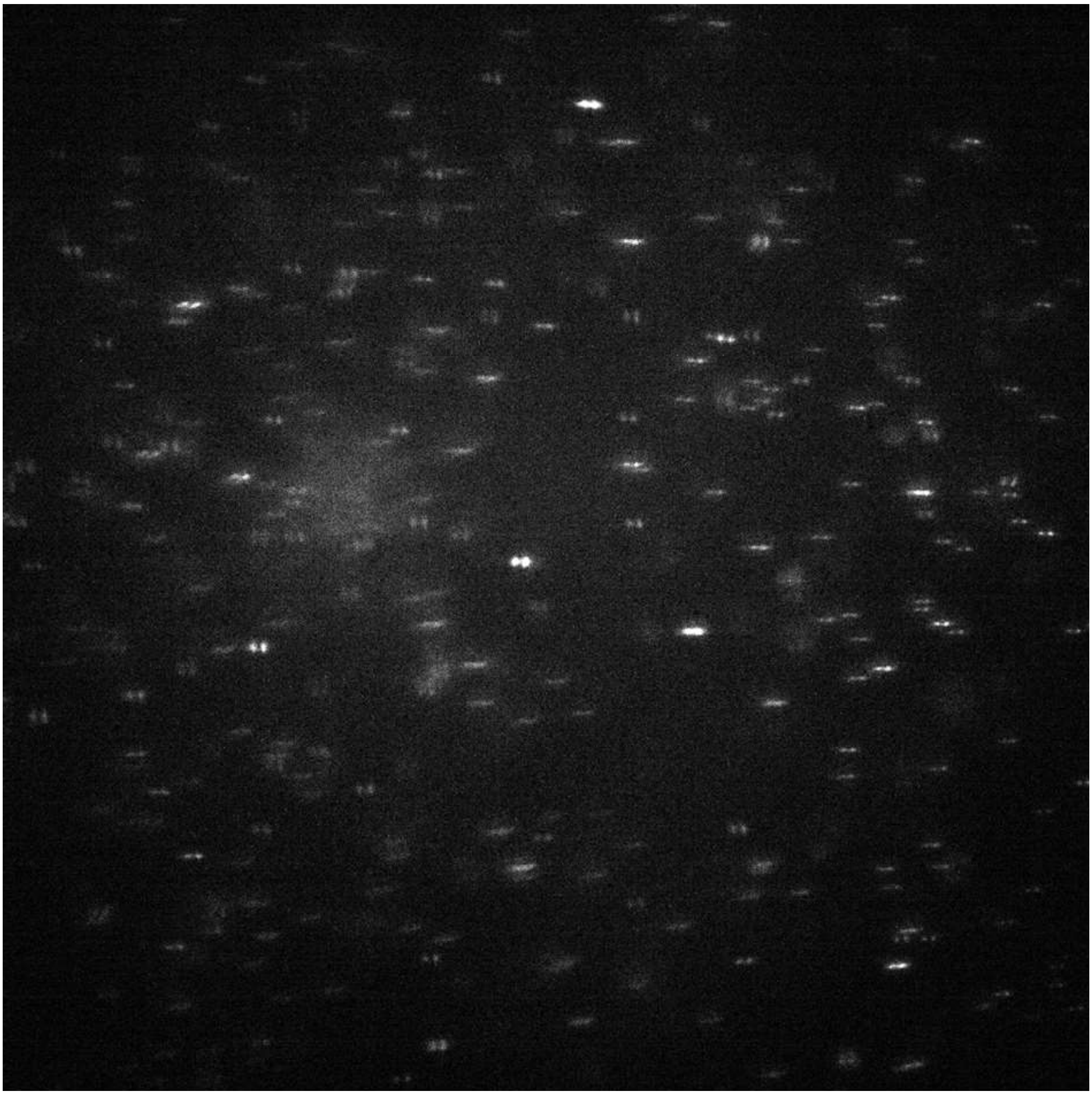

Figure S40. Full frame of Figure 6, depth 0 µm (image size 1024 px × 1024 px; 111 µm × 111 µm).

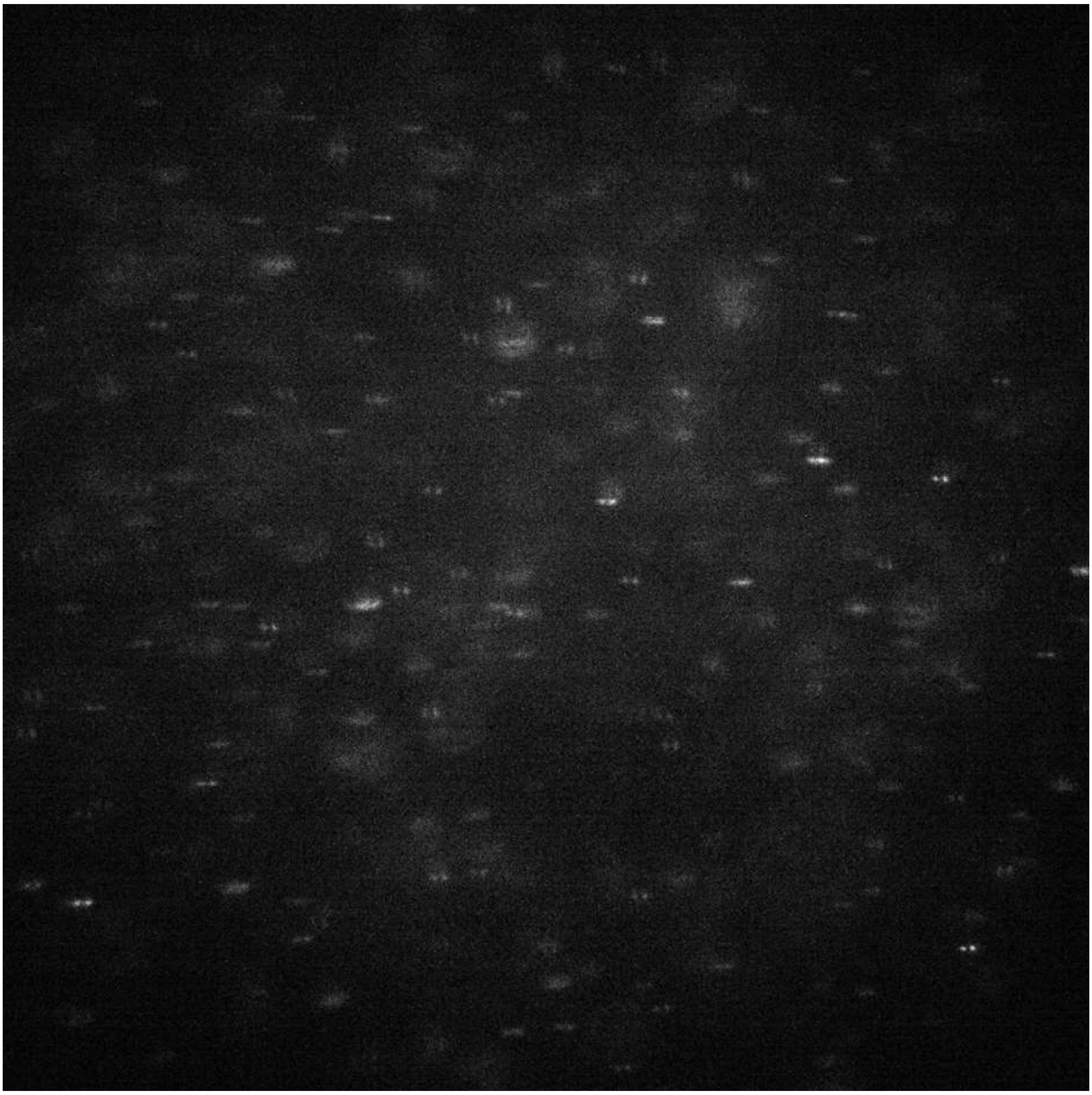

Figure S41. Full frame of Figure 6, depth 6 μm (image size 1024 px × 1024 px; 111 μm × 111 μm).

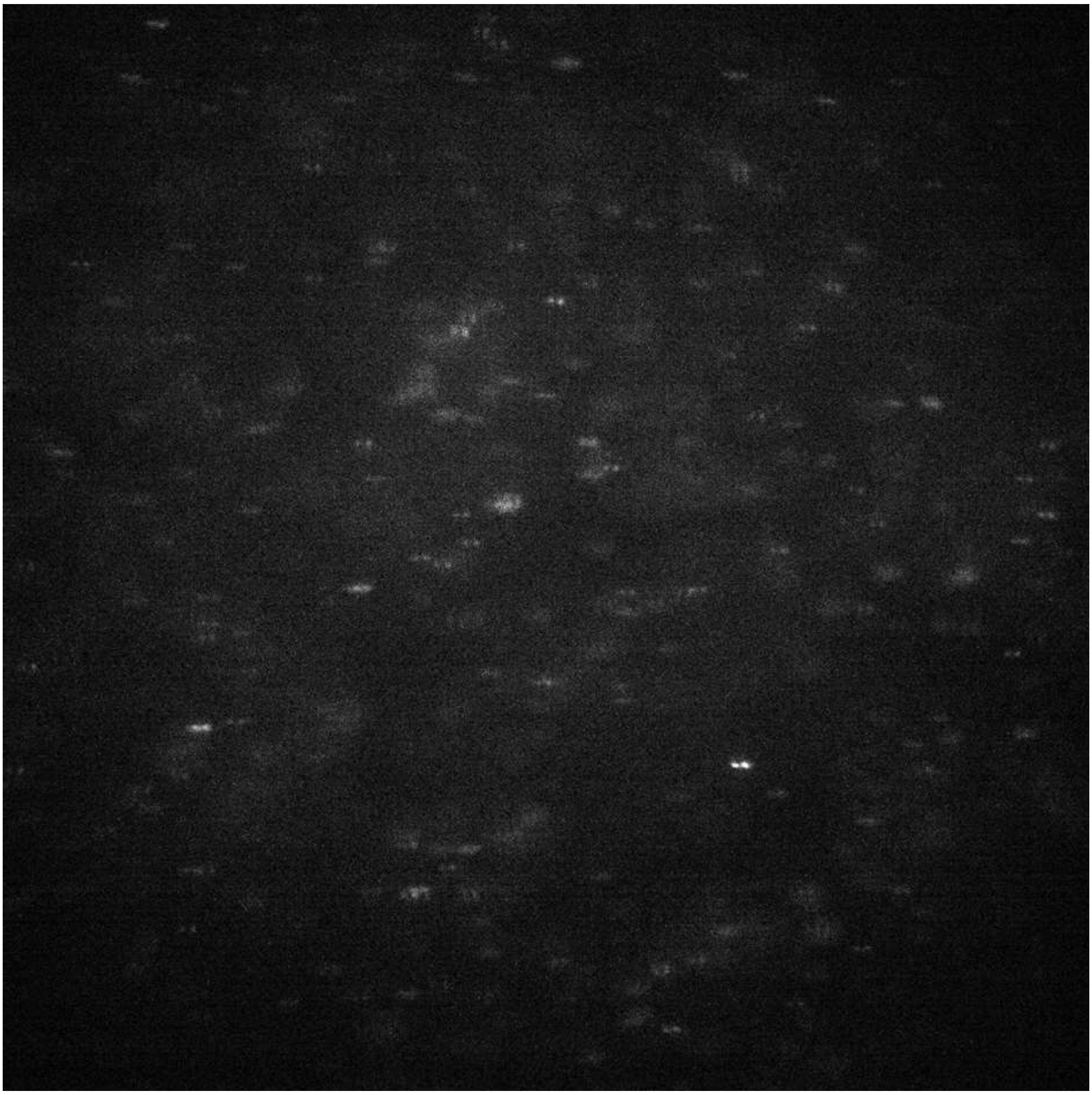

Figure S42. Full frame of Figure 6, depth 12 μm (image size 1024 px × 1024 px; 111 μm × 111 μm).

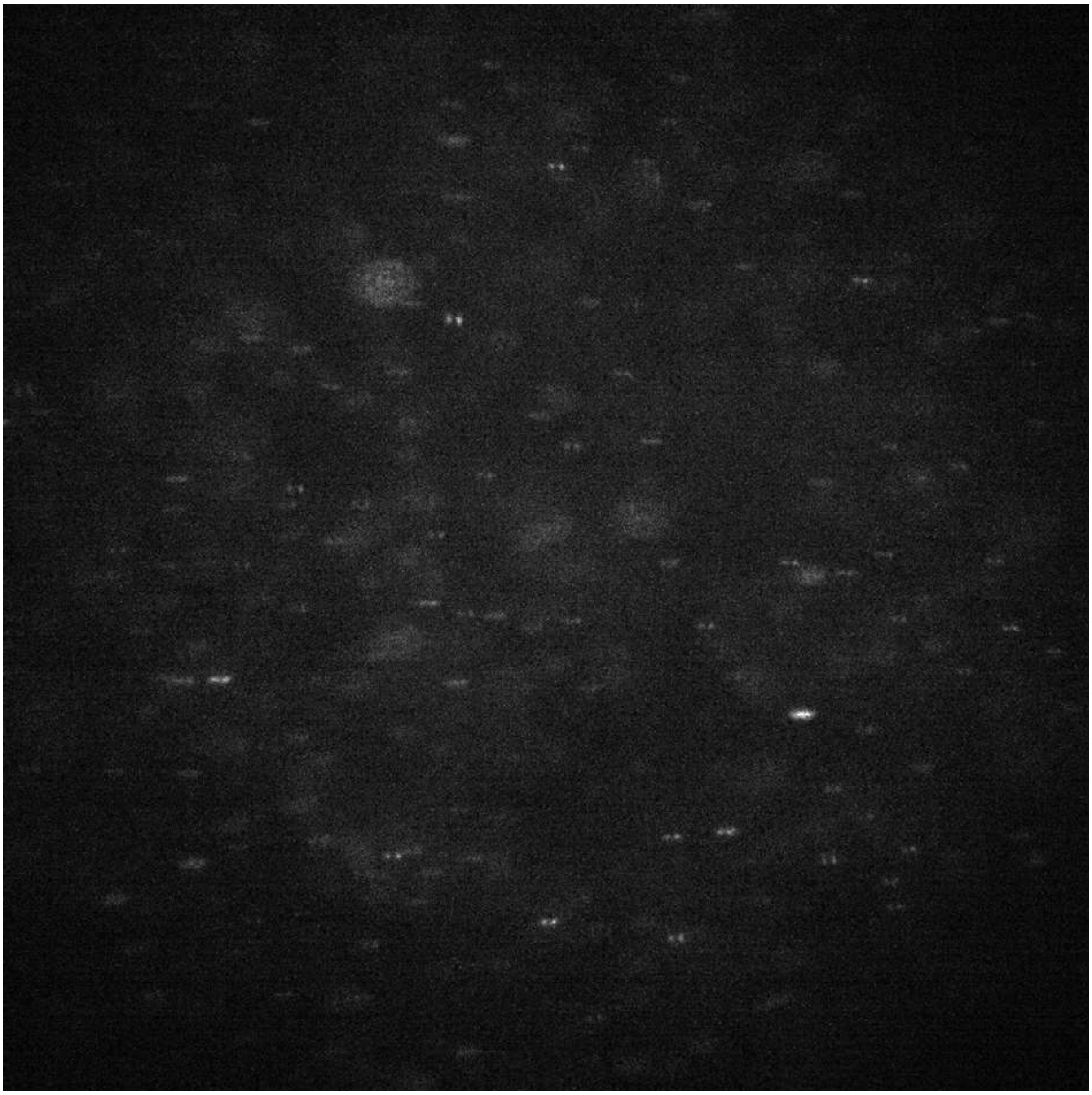

Figure S43. Full frame of Figure 6, depth 18 µm (image size 1024 px × 1024 px; 111 µm × 111 µm).

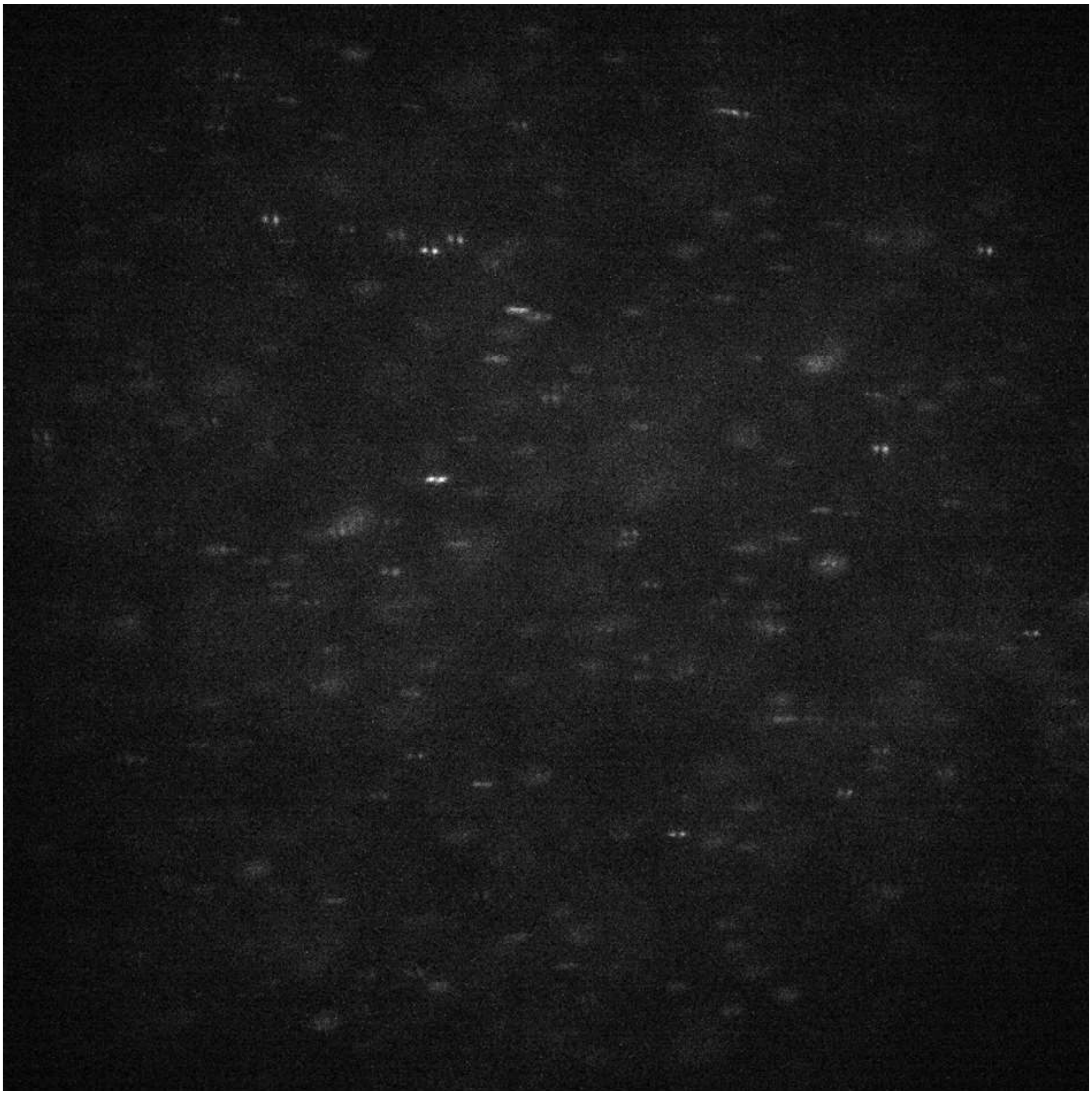

Figure S44. Full frame of Figure 6, depth 24 μm (image size 1024 px × 1024 px; 111 μm × 111 μm).

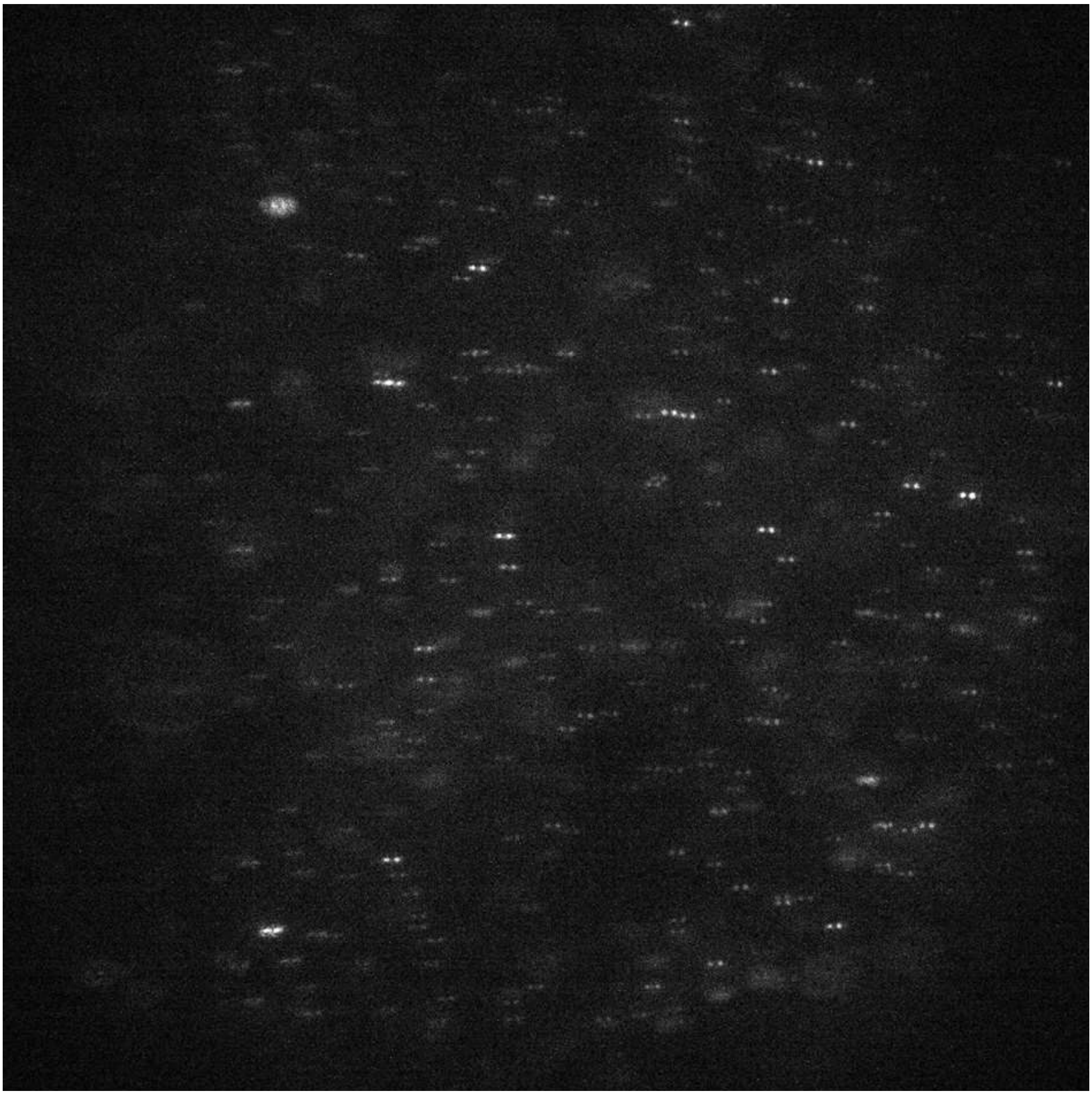

Figure S45. Full frame of Figure 6, depth 30 μm (image size 1024 px × 1024 px; 111 μm × 111 μm).

References

(1) Hermanson, G. *Bioconjugate Techniques*, Second Edition.; Academic Press, 2008.

(2) Hlaváček, A.; Farka, Z.; Mickert, M. J.; Kostiv, U.; Brandmeier, J. C.; Horák, D.; Skládal, P.; Foret, F.; Gorris, H. H. Bioconjugates of Photon-Upconversion Nanoparticles for Cancer Biomarker Detection and Imaging. *Nat. Protoc.* **2022**, *17* (4), 1028–1072. https://doi.org/10.1038/s41596-021-00670-7.

(3) Hlaváček, A.; Křivánková, J.; Brožková, H.; Weisová, J.; Pizúrová, N.; Foret, F. Absolute Counting Method with Multiplexing Capability for Estimating the Number Concentration of Nanoparticles Using Anisotropically Collapsed Gels. *Anal. Chem.* **2022**, *94* (41), 14340–14348. https://doi.org/10.1021/acs.analchem.2c02989.
